# In vitro synthesis of 32 translation-factor proteins from a single template reveals impaired ribosomal processivity

**DOI:** 10.1038/s41598-020-80827-8

**Published:** 2021-01-21

**Authors:** Anne Doerr, David Foschepoth, Anthony C. Forster, Christophe Danelon

**Affiliations:** 1 Department of Bionanoscience, Kavli Institute of Nanoscience, Delft University of Technology, Van der Maasweg 9, 2629HZ Delft, The Netherlands; 2 Department of Cell and Molecular Biology, Uppsala University, 751 24 Uppsala, Sweden

**Keywords:** Proteomics, Synthetic biology, Biosynthesis, Translation

## Abstract

The Protein synthesis Using Recombinant Elements (PURE) system enables transcription and translation of a DNA template from purified components. Therefore, the PURE system-catalyzed generation of RNAs and proteins constituting the PURE system itself represents a major challenge toward a self-replicating minimal cell. In this work, we show that all translation factors (except elongation factor Tu) and 20 aminoacyl-tRNA synthetases can be expressed in the PURE system from a single plasmid encoding 32 proteins in 30 cistrons. Cell-free synthesis of all 32 proteins is confirmed by quantitative mass spectrometry-based proteomic analysis using isotopically labeled amino acids. We find that a significant fraction of the gene products consists of proteins missing their C-terminal ends. The per-codon processivity loss that we measure lies between 1.3 × 10^–3^ and 13.2 × 10^–3^, depending on the expression conditions, the version of the PURE system, and the coding sequence. These values are 5 to 50 times higher than those measured in vivo in *E. coli*. With such an impaired processivity, a considerable fraction of the biosynthesis capacity of the PURE system is wasted, posing an unforeseen challenge toward the development of a self-regenerating PURE system.

## Introduction

**Figure 1:**
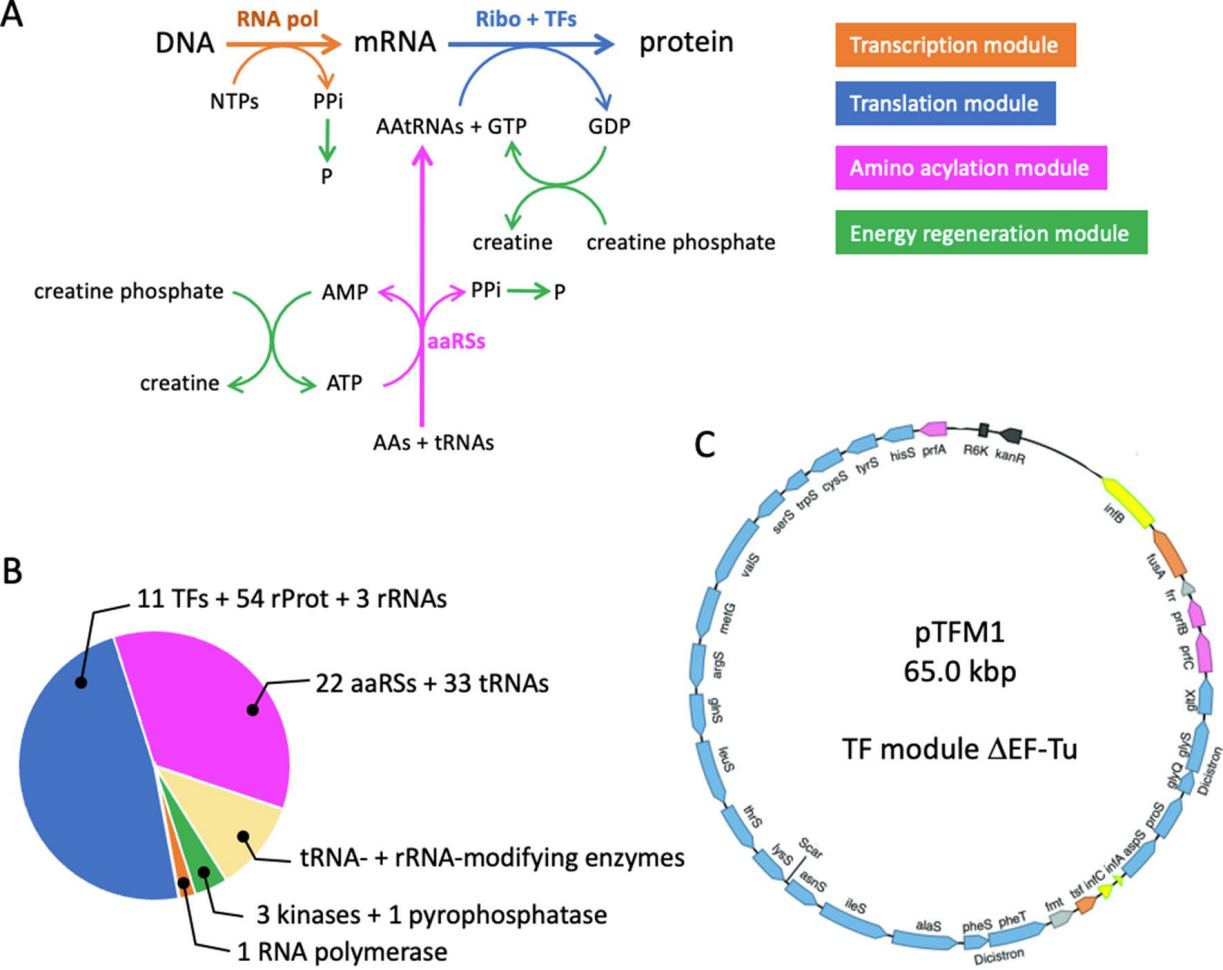
Regenerating the PURE system from genes. (**A**) Schematic of the basic biochemical reactions in the PURE system. The set of reactions is broken down in four main modules. The fuel molecule is creatine phosphate. Hydrolysis of pyrophosphate (PPi) into phosphate (P) is catalyzed by a pyrophosphatase. (**B**) Macromolecules constituting the PURE system, plus the tRNA- and rRNA-modifying enzymes that will eventually have to be synthesized in vitro for complete PURE system biogenesis. Note that 22 proteins constitute the 20 aaRSs. (**C**) Schematic map of the pTFM1 plasmid encoding all aminoacyl-tRNA synthetases (aaRSs) and translation factors (TFs), except EF-Tu. Adapted from Ref.^13^. Other abbreviations: *AAs* amino acids, *AAtRNAs* aminoacyl-tRNAs, *aaRSs* aminoacyl-tRNA synthetases, *RNA pol* RNA polymerase, *Ribo* ribosome, *rProt* ribosomal protein.

The creation of a man-made cellular system capable of autonomous replication is a grand challenge in synthetic biology^1–4^. It will be recognized as a milestone toward the bottom up construction of a minimal cell, and may shed light on the elementary constituents and processes that led to the emergence of early cells. Several cellular models that respond to the basic criteria of self-replication have been proposed and their in vitro construction has been experimentally challenged. The putative ‘RNA cell’ relies on two catalytic RNA molecules, called ribozymes, encapsulated in a vesicle^1^. Despite the apparent simplicity of this scenario, ribozymes that are able to self-replicate or catalyze the formation of membrane constituents from precursors do not exist yet. Alternatively, a ‘ribosome cell’ based on the extant biology might be more amenable to practical realization, although it is composed of many more components than an RNA cell. Translation of genetic information into proteins by the ribosome is universal to all living organisms, including to reduced bacterial cells. A major achievement within the conceptual framework of a ‘ribosome cell’ was the reconstitution of the *E. coli* translation machinery from purified factors^5,6^, a technology known as the PURE (Protein synthesis Using Recombinant Elements) system. Essential components of the PURE system are the T7 RNA polymerase for transcription, the *E. coli* ribosome, tRNAs and 31 translation factors, including the 20 aminoacyl-tRNA-ligases (aaRSs), as well as the translation initiation, elongation and release factors (Fig. 1A,B). Hence, regenerating the PURE system components from a minimal genomic DNA represents a major challenge towards a self-reproducing ‘ribosome cell’.

Several constituents of the PURE system have already been produced in the PURE system itself starting from genes. Awai et al. showed that 19 of the 20 aaRS enzymes could be synthesized in a soluble and active form^7^. In this study, the 20 aaRSs were expressed individually in a PURE system that contained lowered input concentration of the aaRS that was expressed, so that the activity of the de novo synthesized protein could be detected above the background level of activity stemming from the originally supplied aaRS. Attempts to reconstruct the *E. coli* ribosome were carried out in the PURE system by expressing the ribosomal proteins^8^. While all 54 proteins could be detected when produced separately, and all of the 21 proteins of the small subunit when co-expressed, only 29 of the 33 large ribosomal subunit proteins were detected in co-expression reactions. To differentiate newly synthesized components from the PURE system background, the reaction mixture was supplied with isotopically heavy arginine and lysine residues, and the translation products were detected by mass spectrometry similar to the well-established method of stable isotope labeling with amino acids in cell culture (SILAC)^9^. Hence, this strategy allows for quantification of the synthesized protein relative to the originally supplied protein in the PURE system. Efforts to synthesize the three ribosomal RNAs (rRNAs) are challenged by the numerous chemical modifications underwent by the 16S and 23S rRNAs to harbor their full activity spectrum^10^. To bypass the reconstitution of the enzymatic rRNA modification pathway in the PURE system, in vitro evolution has been applied to generate a 16S rRNA mutant that is active in the absence of post-transcriptional modifications^11^. Furthermore, 48 *E. coli* tRNAs have been synthesized in vitro from separate DNA templates using the T7 RNA polymerase, and most of them showed functionality in an *E. coli* cell-free translation system^12^. Actually, it has been proposed that only 33 *E. coli*-based tRNAs would be sufficient to decode all 20 amino acids^2^. The other PURE system elements that have to be regenerated as well are: the methionyl-tRNA-formyltransferase, T7 RNA polymerase, pyrophosphatase, and the enzymes of the energy recycling module, creatine kinase, myokinase and nucleoside-diphosphate kinase.

Herein, we show that all translation factors (TFs) (except elongation factor Tu, EF-Tu) and aaRSs can be expressed from a single plasmid in the PURE system. In the following, aaRSs will also be referred as TFs. We used the pTFM1 plasmid encoding 32 proteins in 30 cistrons: 20 aaRSs, three translation initiation factors, three release factors, ribosome recycling factor, two elongation factors, and the methionyl-tRNA-formyltransferase (Fig. 1C)^13^. All genes contain the same T7 promoter-lacO-RBS (RBS, ribosome binding site) block at the 5′ end of the coding sequence and T7 phi terminator at the 3′ end. Only the two aaRSs consisting of two subunits, namely the glycine tRNA-ligase and phenylalanine tRNA-ligase, are encoded as two-gene cistrons. We were able to detect the synthesis of all 32 proteins from a single plasmid. Moreover, we discovered that many truncated proteins were also generated, an issue that remains under-appreciated when interpreting the outcome of cell-free gene expression reactions.

## Results

### LC–MS detection of 32 proteins expressed in PURE system from a 30-cistron TF module plasmid

**Figure 2:**
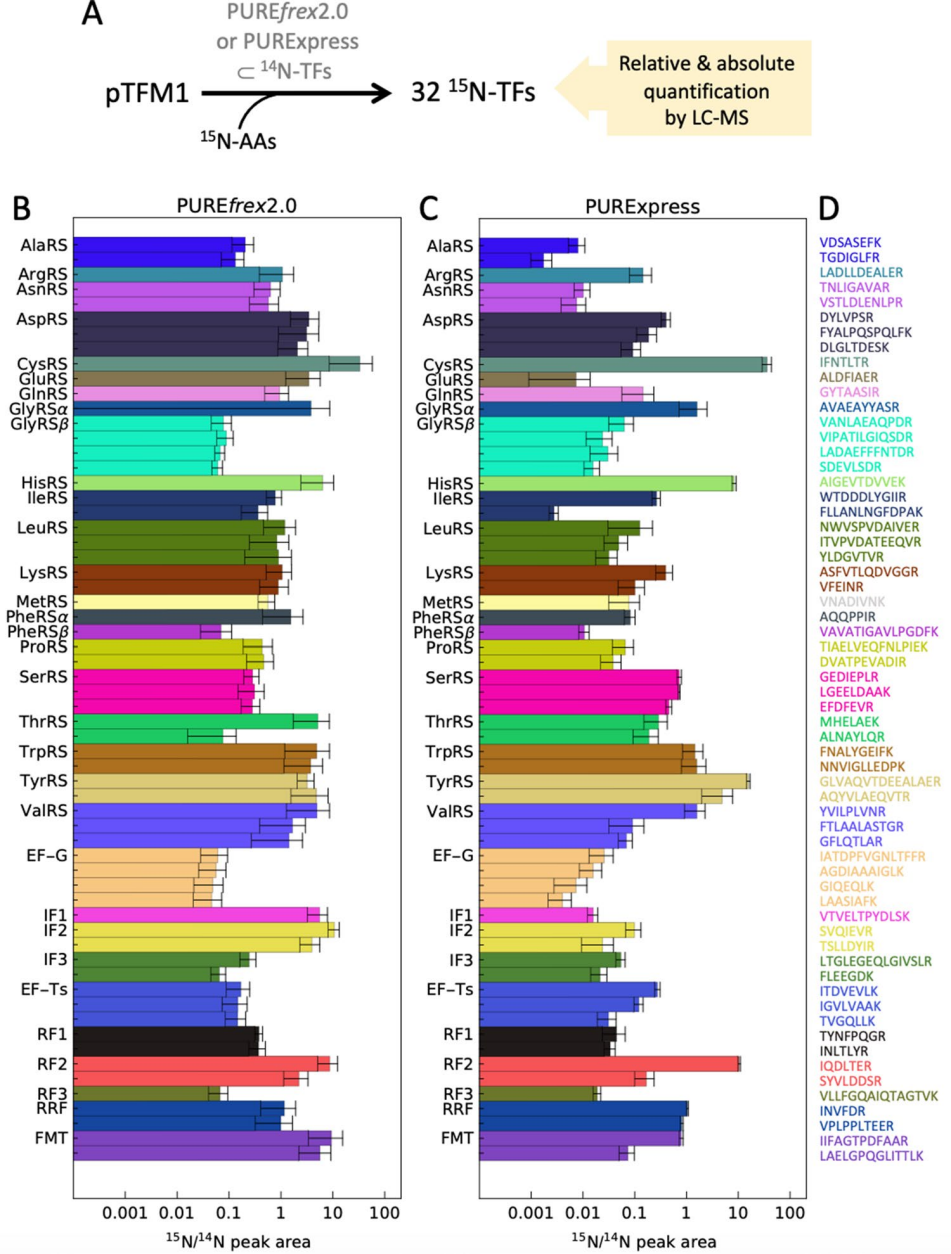
In vitro production of 32 translation factor proteins from a single plasmid. (**A**) Reaction scheme for pTFM1 expression in the PURE system. *AAs* amino acids, *TFs* translation factors, *FMT* methionyl-tRNA-formyltransferase, *IF* initiation factor, *EF* elongation factor, *RF* release factor, *RRF* ribosome recycling factor. (**B**,**C**) Relative expression levels of proteins expressed from pTFM1 with PURE*frex*2.0 (**B**) and PURExpress (**C**) after 5 h incubation at 37 °C. Ratios of ^15^N-labeled peptides (newly synthesized proteins) to ^14^N peptides (original proteins) are displayed for all measured peptides; for proteins with multiple measured peptides, the peptides are ordered from N- to C-terminus (top to bottom). Error bars denote ± 1 standard deviation from triplicate experiments. Results from a different batch of PURE*frex*2.0 are shown in Supplementary Fig. 2. (**D**) Specific peptides measured in (**B**) and (**C**).

To detect proteins expressed with the PURE system from pTFM1 we used heavy isotope labeling and liquid chromatography-coupled mass spectrometry (LC–MS)^8^. We employed two commercially available versions of the PURE system, PURE*frex*2.0 and PURExpress, in combination with a buffer containing ^15^N-labeled amino acids (Supplementary Table 1), so that all newly synthesized proteins contain ^15^N-amino acids and the concentration ratio of in situ expressed to originally supplied proteins can be determined by mass spectrometry (Fig. 2A). We assayed the detection efficiency of different candidate tryptic peptides by LC–MS/MS using Skyline^14^ and reaction monitoring guided by the *E. coli* MG1655 spectral library (The Global Proteome Machine, https://www.thegpm.org/). We finally selected a set of 64 peptides, each of the 32 proteins encoded by pTFM1 plus EF-Tu being covered by one to four peptides (Fig. 2D, Supplementary Tables 2 and 3) which could be measured in two LC–MS/MS runs with multiple reaction monitoring (MRM). We optimized trypsin reaction conditions to achieve complete digestion (Supplementary Fig. 1). As shown in Fig. 2B,C, all 32 proteins could be detected for both PURE*frex*2.0 and PURExpress. The ratios of the peak areas of the ^15^N-labeled peptides to ^14^N-labeled peptides is a measure of the protein expression levels relative to the amount of the respective protein originally present in the PURE system. We found that the relative protein expression levels varied between 2 and 3000% and were generally higher for PURE*frex*2.0 than for PURExpress.

### PURE system produces C-terminal truncated proteins over time

**Figure 3:**
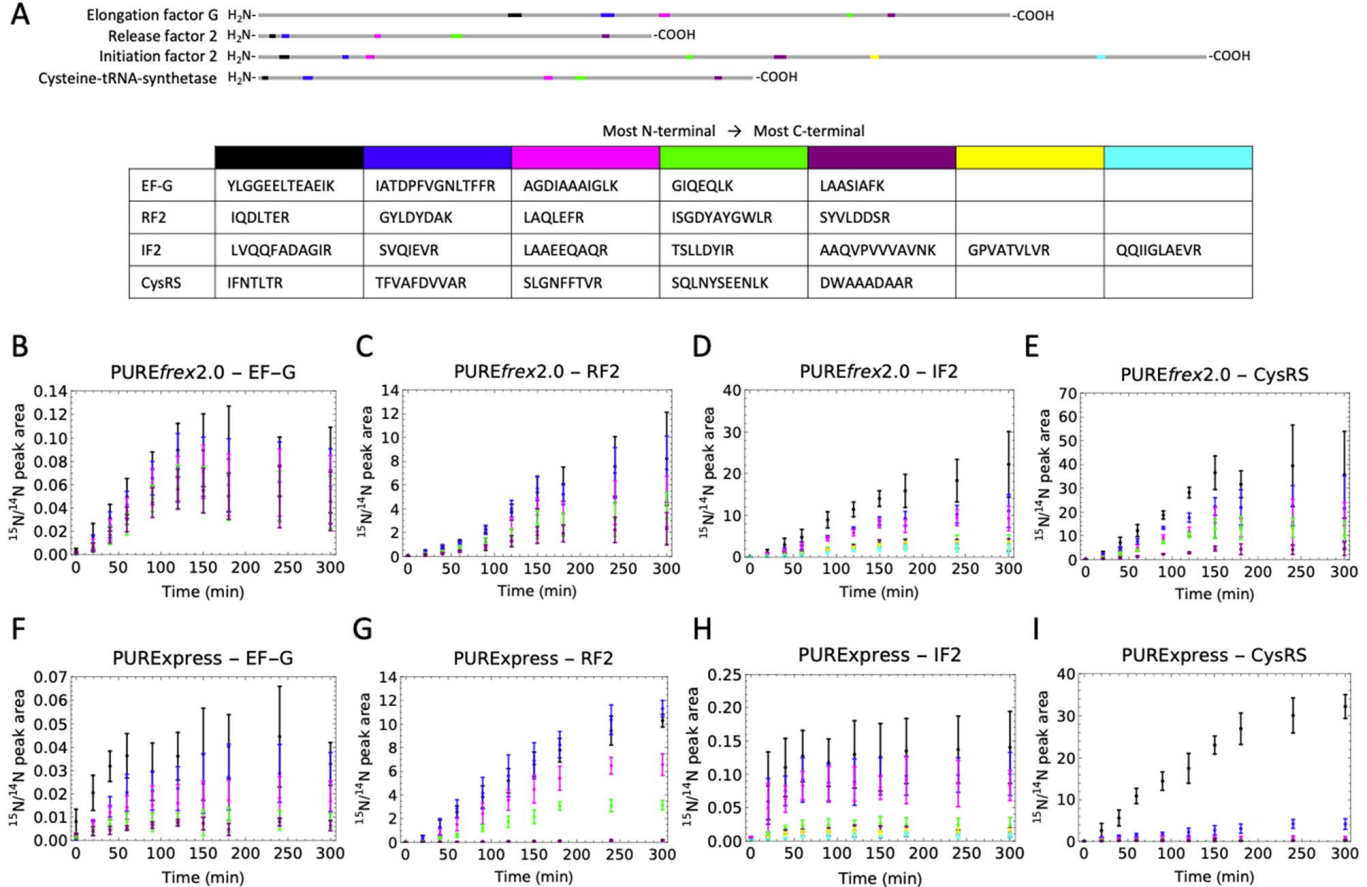
Time-course of ^15^N-to-^14^N ratio of selected peptides for four proteins encoded in pTFM1. (**A**) Schematic of the full-length proteins displaying the positions of selected proteolytic peptides. For each cell-free synthesized protein, the set of signature peptides is chosen to span the entire polypeptide chain from the N- to C-termini. Color coding from N- to C-terminal peptides: black, blue, pink, green, magenta, yellow, cyan. The set of specific peptides measured for all four proteins is also indicated. *EF-G* elongation factor G, *RF2* release factor 2, *IF2* initiation factor 2, *CysRS* cysteine-tRNA-ligase. (**B–I**) Relative expression levels of the indicated peptides for the four annotated proteins with PURE*frex*2.0 (**B–E**) and with PURExpress (**F–I**). Color coding of the peptides is similar as in (**A**). The five kinetics plots in (**B**) are displayed in separate graphs in Supplementary Fig. 3. All reactions were performed at 37 °C. Error bars denote ± 1 standard deviation from triplicate experiments.

**Figure 4:**
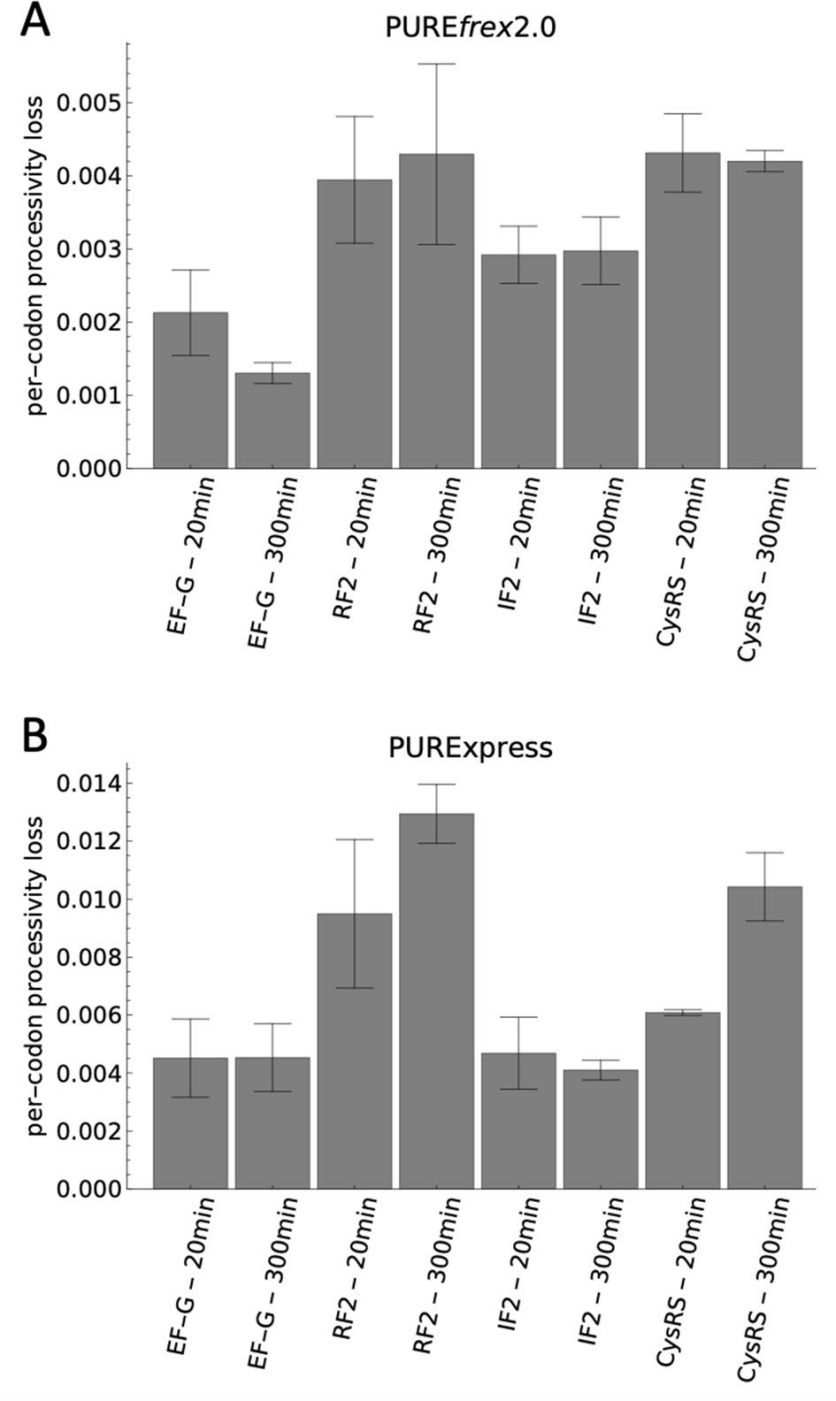
Quantification of processivity loss with pTFM1 as a template. Average per-codon processivity loss of the indicated proteins expressed in PURE*frex*2.0 (**A**) or in PURExpress (**B**) for 20 min or 300 min. Bar heights are slope values obtained from weighted fitting of data shown in Supplementary Fig. 4 and error bars are weighted fit uncertainties.

We noticed that for some proteins with multiple measured peptides the ^15^N-to-^14^N ratio tended to decrease from the more N-terminal peptides to the more C-terminal peptides, indicating synthesis of truncated products (Fig. 2B,C, Supplementary Fig. 2). This trend was noticeable for ~ 10 proteins (depending on the batch of the kit) out of 21 with PURE*frex*2.0 and for ~ 16 proteins out of 21 with PURExpress. To investigate this effect in more detail we selected another set of peptides (Supplementary Table 4) covering four of the proteins (EF-G, RF2, IF2 and CysRS) from N- to C-terminus (Fig. 3A), and measured the ^15^N-to-^14^N ratio for these peptides over time (Fig. 3B–I). We chose these proteins because they each represent a different class of translation factor and because we identified a sufficient number of unique peptides that span the entire sequence of the corresponding protein. For all four proteins, a continuous decrease of the ^15^N-to-^14^N ratio towards the more C-terminal peptides was observed for all measured time points in both versions of the PURE system (Fig. 3B–I, note that the EF-G result with PURE*frex*2.0 is more significant here than in Fig. 2B partly because of the inclusion of an additional peptide). This result confirms that the processivity of translation elongation is impaired for several proteins. Moreover, this behavior is not an artefact due to long expression times, when the system possibly runs out of nutrients or some of the protein machinery becomes inactive. Premature translation arrest also occurs at the start of the reaction, as indicated by the overabundance of N-terminal peptides within the first 40 min of expression (Fig. 3B–I). Therefore, this process does not result from detrimental effects that would become more prominent in the course of expression, like mRNA degradation. The average per-codon loss of processivity was calculated as the negative exponent of an exponential regression fit to the ^15^N-to-^14^N ratio as a function of the peptide position (Supplementary Figs. 4, 5). Values range between 1.3 × 10^–3^ and 4.5 × 10^–3^ for PURE*frex*2.0, and between 4.0 × 10^–3^ and 13.2 × 10^–3^ for PURExpress, with CysRS and RF2 having the highest per-codon loss values in both systems, followed by IF2 and EF-G (Fig. 4). No major differences were observed after 20 min and 300 min expression durations (Fig. 4). These values are an order of magnitude higher than the processivity error measured in *E. coli*^15,16^. Interestingly, the expression lifetime, defined here as the time at which production of the most N-terminal peptide ceases, is different for the four proteins, but also between the two different PURE system versions (Supplementary Fig. 6). For instance, the expression lifetime of IF2 is ~ 415 min in PURE*frex*2.0 and ~ 39 min in PURExpress, whereas the expression lifetime of CysRS is ~ 151 min in PURE*frex*2.0 and ~ 339 min in PURExpress. This result shows that both the coding sequence of the DNA template and the nature of the cell-free gene expression system influence the kinetics of protein synthesis.

**Figure 5:**
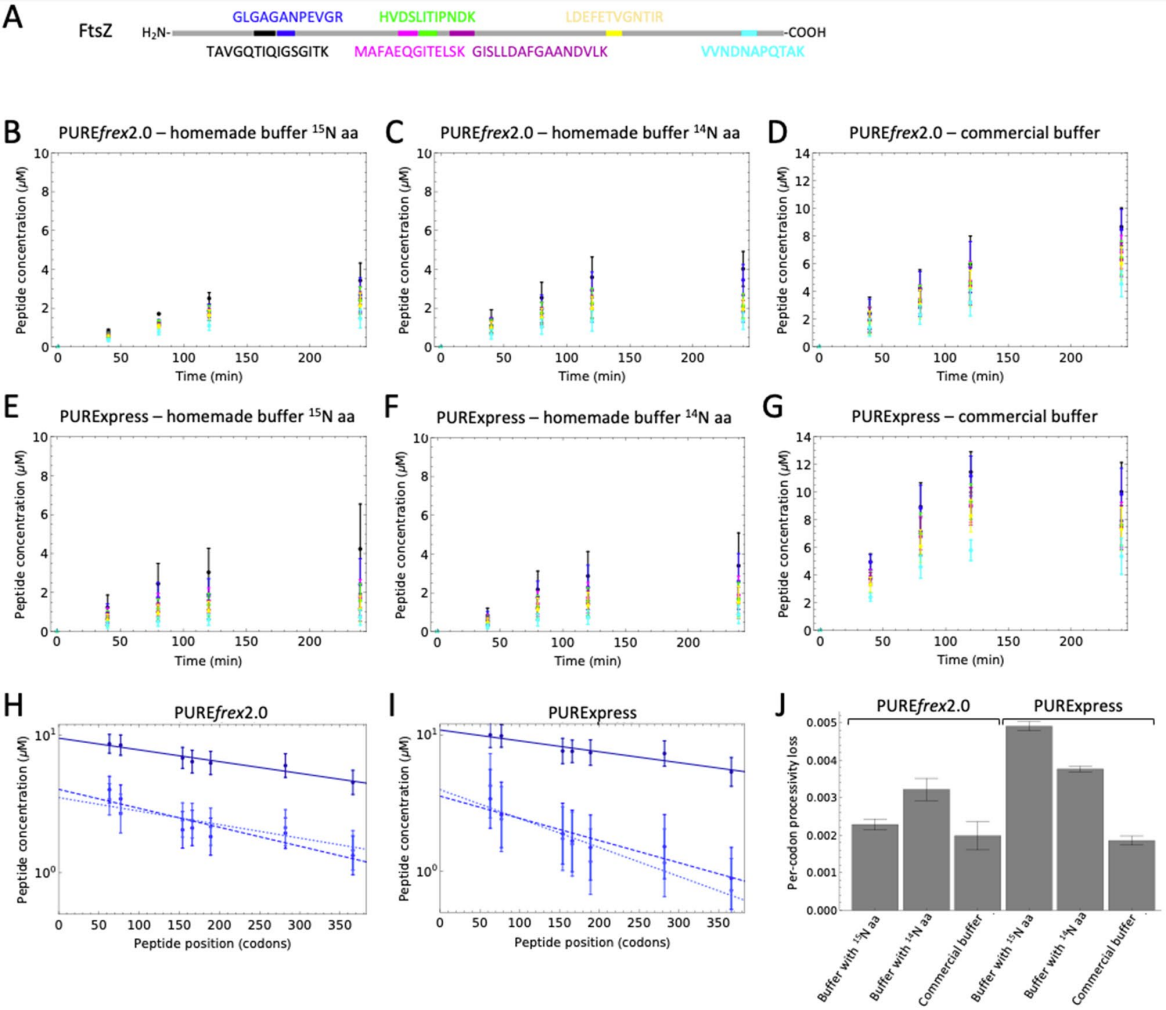
Impaired ribosome processivity occurs also in single-gene expression reactions. (**A**) Schematic of the full-length FtsZ protein displaying the positions of selected peptides. The specific peptides measured are appended. Color coding from N- to C-terminal: black, blue, pink, green, magenta, yellow, cyan. (**B**–**G**) Time-course of the concentration of the indicated peptides synthesized with PURE*frex*2.0 (**B–D**) and with PURExpress (**E**–**G**). All reactions were performed at 37 °C. Error bars denote ± 1 standard deviation from triplicate experiments. Color coding of the peptides is similar as in (**A**). (**H**,**I**) End-point (5 h) FtsZ peptide concentration as a function of peptide position for reactions with the commercial buffer (dark blue, solid lines), with the homemade buffer containing unlabeled amino acids (blue, dashed lines) or with ^15^N-labeled amino acids (light blue, dotted lines). Straight lines are exponential regression fits. (**J**) Average per-codon processivity loss derived from the slope computed in (**H**,**I**). Error bars are weighted fit uncertainties.

To rule out the possibility that processivity errors originate either from an artefact of the homemade buffer that we used instead of the commercially supplied buffers (in order to substitute the unlabeled amino acids with ^15^N-labeled ones), or from the co-expression of 32 proteins, we performed control experiments in which a single protein, the bacterial tubulin homolog FtsZ, was expressed in both PURE system versions either with the buffer provided with the commercial kits or with our homemade buffer. In the latter case, the amino acid mix consisted of either ^14^N-labeled amino acids (each in equimolar amounts), or the same ^15^N-labeled amino acid mix as used for pTFM1 expression. Purified FtsZ protein, either unlabeled or ^15^N-labeled, was used as an internal standard to quantify the absolute concentrations of the synthesized peptides. We selected a set of seven FtsZ peptides that could be monitored in a single MRM experiment (Fig. 5A, Supplementary Table 5). Expression of this single protein resulted also in a significant decrease in concentration from N-terminal to more C-terminal peptides for all conditions and time points (Fig. 5B–G). Yield of synthesized FtsZ peptides was significantly higher with the commercial buffers (the most C-terminal peptide reached a concentration of ~ 5 µM) than with the homemade ones, while yields between the unlabeled amino acid mix and the ^15^N-labeled one were negligible in both versions of PURE system. For PURE*frex*2.0, the processivity was similar for all tested buffers, while for PURExpress the processivity was lower in the homemade buffers compared to the commercial ones (Fig. 5H–J). This result suggests that, also in the case of expression from pTFM1, lower processivity in PURExpress might be an effect of the buffer rather than an intrinsically lower performance of PURExpress as compared to PURE*frex*2.0.

### Absolute quantification of translation factors expressed from pTFM1 in PURE system

**Figure 6:**
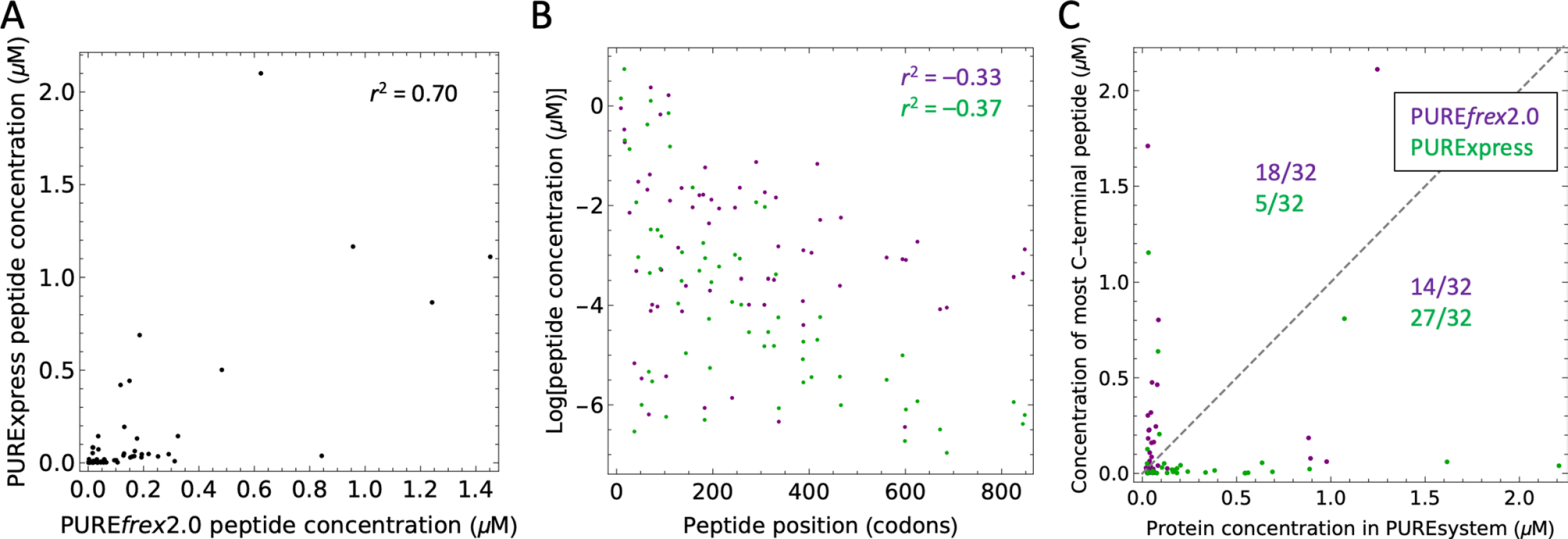
Correlation analyses of absolute peptide concentrations for pTFM1 expression. Correlations of absolute peptide expression levels between PURE*frex*2.0 and PURExpress (**A**), with the peptide position within the coding sequence (**B**), and with their initial amounts in the commercial kits (**C**). (**A**,**B**) Values of *r*^2^ denote Pearson correlation coefficients. (**B**,**C**) Data points for PURE*frex*2.0 and PURExpress are colored in magenta and in green, respectively. (**C**) The most C-terminal peptide for each protein was used for analysis. The appended dashed line depicts the slope equal to 1. PURE*frex*2.0 and PURExpress reactions were run in the homemade buffer containing ^15^N-labeled amino acids. Concentrations of synthesized proteins were derived from ^15^N-to-^14^N peak ratios, while the concentrations of PURE system components were determined in separate experiments using QconCAT as an internal standard. For each PURE system kit, the number of proteins whose concentration falls above ([protein]_expressed_ > [protein]_input_) or below ([protein]_expressed_ < [protein]_input_) the diagonal is indicated.

The ratios of ^15^N-to-^14^N peak intensities do not allow for a comparison between expression levels in reactions with PURE*frex*2.0 and PURExpress, nor between different proteins within the same reactions. With the aim to provide absolute quantification of PURE system TFs, we designed a QconCAT (Quantification conCATemer), an artificial protein generated by concatenation of proteolytic peptides used as reference standards for quantification of the corresponding TF peptides. Our QconCAT is composed of one to four peptides from all proteins encoded on pTFM1, as well as two peptides from EF-Tu and two quantification peptides (Supplementary Figs. 7, 8). Although the QconCAT protein expressed well in lysogeny broth (LB) medium, we were unable to purify the protein from *E. coli* cells grown in isotope-labeled medium. Therefore, the DNA sequence was split into two halves and both halves were recloned into a pRSET-B vector harboring an N-terminal His-tag. After testing the purified, isotope-labeled QconCAT halves against the purified, unlabeled full-length QconCAT, we determined the concentrations of all corresponding proteins in PURE*frex*2.0 and PURExpress. For proteins with multiple peptides, the concentration differences were within the error margin. From these measurements, we could calculate the absolute concentrations of the peptides expressed from pTFM1 in both PURE systems. Concentration values span a few orders of magnitude, with most peptides having a concentration below 0.2 µM and only a few reaching the micromolar range (Fig. 6). Absolute expression levels are significantly correlated between the two systems with a correlation coefficient of 0.70 (Fig. 6A). This correlation can in part be explained by the negative correlation of the peptide concentration with respect to its position within the coding sequence due to processivity errors (Fig. 6B). Moreover, significant disparity in expression levels between the different proteins was observed even when comparing peptides located at roughly the same position in the primary sequence. We then assessed the correlation of the measured peptide concentration against predictions from three different mRNA design tools: RBS calculator, RBS designer and UTR designer^17–19^, as well as an empirical 3-codon score^20^. The peptides belonging to the beta subunits of the glycine- and phenylalanine-tRNA ligases were excluded from the analysis because these proteins are expressed as second protein of an operon with the corresponding alpha subunits, which is expected to influence the expression level. No significant correlations were found for any of the tested predictive tools (Supplementary Fig. 9).

Finally, we compared the absolute concentration of all 32 synthesized proteins with respect to their original concentrations in PURE*frex*2.0 and PURExpress (Fig. 6C). The most C-terminal peptide for each translation factor was used as it best estimates the amount of full-length protein. Under these expression conditions, PURE*frex*2.0 is able to produce more proteins than initially contained in the commercial kit for > 50% of the TFs, versus ~ 15% for PURExpress. For proprietary reasons, we cannot specify which protein corresponds to which data point in Fig. 6C, as it would conflict with the policy of New England Biolabs and GeneFrontier Corporation to not reverse engineer their products. Whereas processivity loss is similar for both PURE systems in their respective commercial buffers, translation elongation is particularly affected in the homemade buffer with ^15^N-labeled amino acids for PURExpress compared to PURE*frex*2.0 (Fig. 5J). Therefore, we expect that expression of pTFM1 in optimal reactions would lead to doubled (or more) concentrations for a higher fraction of TFs, and this effect would be more pronounced for PURExpress than for PURE*frex*2.0.

## Discussion

Co-expression of 32 different proteins from a single 30-cistron plasmid was realized in the PURE system. Because the gene products are constituents of the PURE system itself, this work contributes to ongoing efforts to regenerate a minimal protein synthesis machinery from a DNA template^7,8,11^.

Detection of C-terminal truncated translation products reveals hampered ribosomal processivity in the PURE system. This process seems to be a general bottleneck as it affects the yield of synthesized full-length protein for many of the 33 genes expressed using two different PURE system variants, in single-gene as well as in 30-cistron expression reactions. The per-codon processivity loss is 5–50 times higher than that measured in *E. coli*^15,16,21,22^. Production of truncated products with the PURE system, but also with cell lysates, has been reported before, in particular with the expression of eukaryotic proteins^23–26^. Here, we show that processivity of translation elongation is significantly hampered with native *E. coli* sequences under various cell-free gene expression conditions.

Processivity issues were not mentioned in two recent studies, where ribosomal proteins^8^ or translation factors^27^ expressed in PURE system were also analyzed by LC–MS. In the latter study, TFs were expressed from three different plasmids in PURExpress^27^. We imported the MaxQuant ouput MS data from Ref.^27^ and plotted them as displayed in Supplementary Figs. 4, 5 to verify the occurrence of processivity errors (Supplementary Data file). A clear trend showing a decreased abundance of C-terminal peptides was observed for at least 18 out of the 32 proteins (Supplementary Data file). This finding emphasizes the need for systematic investigations of all gene products for unbiased monitoring and quantification of expressed proteins. Comparing the ratios of ^15^N-to-^14^N peak intensities for the different TFs obtained in the study of Libicher et al.^27^ and ours, we found a rather low correlation with Pearson correlation coefficients ~ 0.5 (Supplementary Fig. 10A,B). Besides, no correlation was found when comparing the protein concentrations quantitated in our study and in Fig. 2F of Shepherd et al.^13^ (Supplementary Fig. 10C,D). These differences may originate from the different DNA templates (single plasmid vs three plasmids in Refs.^13,27^), PURE compositions (PURE*frex*2.0 vs modified PURExpress in Ref.^27^), expression conditions (in vitro vs in vivo in Ref.^13^) or quantification methods.

Possible causes for the observed processivity errors of translation elongation include ribosome stalling or destabilization, peptidyl-tRNA drop-off and premature termination. Supplementing the PURE system with ribosome rescue factors^28–33^, peptidyl-tRNA-hydrolase^23,34^, EF-G^35–38^, methylated RF1 and RF2^39^, or the ribosomal protein bL31^40,41^ might therefore enhance processivity. Degradation of mRNA by nuclease contaminants is not substantial in the PURE system compared to cell extracts^34,42,43^. Given that proteins missing their C-terminal ends are already detected at short incubation times (Figs. 3, 4), stalling of translating ribosomes on mRNA truncated at the 3′ end is unlikely the main cause of impaired processivity. Moreover, identical results were obtained when the murine RNase inhibitor was supplied to PURE system reactions (data not shown). Overall, a complex set of side reactions may impede translation elongation in the PURE system. The fact that PURExpress is more susceptible to processivity errors than PURE*frex*2.0 under the tested conditions indicates that optimization of the abundance and stoichiometric amount of the different components might help improve both the expression yield and translation processivity. In addition to optimizing the protein hardware of the PURE system, optimization of buffer components may also increase the system performance. This idea is supported by the observation that expression yield and processivity were improved in PURExpress when using the commercial buffer instead of the homemade one. In particular, magnesium ion and spermidine concentrations have a huge effect on many of the individual rates, in particular the EF-G-catalyzed translocation reaction, as well as peptidyl transfer. While translocation is faster at lower Mg^2+^ concentrations^44^, ternary complex binding and peptidyl transfer are however faster at higher Mg^2+^ concentrations, albeit coupled to a trade-off between rate and fidelity^45^. Systematic attempts to improve the PURE system by varying its composition have revealed complex interactions between different components^46–49^, further challenging the formulation of a high-fidelity, high-yielding gene expression system by rational design.

The biosynthesis capacity of the PURE system is one to two orders of magnitude lower than the yield required to reproduce the input proteins^50^. Such a suboptimal performance precludes the realization of a self-replicating PURE machinery. In fact, optimizing the PURE system composition and DNA sequence for better usage of resources and higher fidelity of translation would yield larger amounts of full-length products, without necessarily implying to increase the total mass of synthesized proteins. Furthermore, enhancing translation initiation would increase the fraction of translating ribosomes, hence the amount of output proteins^34^. Other important considerations include the proper folding of the polypeptide chains into functional proteins^51^, as well as the controlled co-expression of multiple proteins required for the reconstitution of complex biological functions. Although we could detect all 32 proteins encoded on pTFM1, controlling expression levels to yield functional feedback of the de novo synthesized translation factors and, hence, more sustainable expression, remains difficult. Absolute quantification of synthesized peptides has revealed that there exists no correlation of the expression levels with the tested predictive tools (Supplementary Fig. 9). Moreover, further investigations are needed to empirically correlate the amount of a large set of cell-free synthesized proteins with the initial coding sequence^20^. Nonetheless, we observed significantly lower yields for the GlyRS and PheRS beta subunits that were expressed from the second position of a cistron (the genes of their respective alpha subunits were in the first position) (Fig. 2B,C), as previously reported for similarly designed constructs with PURExpress^52^.

## Materials and methods

### DNA constructs

pTFM1 was amplified and purified as previously described^13^. pET11a-ftsZ-his6 was constructed as follows. Gene fragments were PCR amplified from chromosomal *E. coli* BL21 DNA with primers 5′-TTAACTTTAAGAAGGAGATATACATATGTTTGAACCAATGGAACTTACC-3′ and 5′-TCCTTTCGGGCTTTGTTAGCAGCCGGATCCTTAATCAGCTTGCTTACGCAG-3′. These primers contain overhangs for the pET11-a plasmid. Next, the PCR products were digested with DpnI (New England BioLabs Inc.) and assembled to a linearized pET11-a plasmid (equimolar concentrations) via Gibson Assembly for 1 h at 50 °C. The assembly products were transformed into *E. coli* TOP10 competent cells via heat shock, the cells were centrifuged and resuspended in 50 µL of fresh liquid LB media and incubated at 250 rpm for 1 h at 37 °C. Cultures were plated on solid LB medium with 0.05 ng µL^–1^ ampicillin and were grown overnight at 37 °C. Selected colonies were cultured in 1 mL of liquid LB medium with 0.05 µg µL^–1^ ampicillin at 250 rpm for 6 h at 37 °C. Plasmid purification was carried out using PureYield Plasmid Miniprep System (column method, Promega). Production of linear DNA constructs from the above purified plasmids was performed by PCR using primers 5′-TAATACGACTCACTATAGGGGAATTGTGAGCGGATAACAATTCCCCT-3′ and 5′-CAAAAAACCCCTCAAGACCCGTTTAGAGG-3′. PCR products were analyzed on a standard DNA agarose gel (1%; EtBr or SYBR safe).

### Protein purification

^15^N-labeled FtsZ was expressed from a pET11a-ftsZ-his6 vector in *E. coli* C41(DE3). Cells were grown to saturation in LB medium, diluted 1:100 into M9 medium with ^15^NH_4_Cl, grown over-night and again diluted 1:100 into fresh M9 medium containing ^15^NH_4_Cl. At OD_600_ = 0.5, cells were induced with 1 mM IPTG and harvested after 3 h at 37 °C. Unlabeled FtsZ was expressed in *E. coli* C41(DE3) cells in LB medium under the same induction conditions. FtsZ was purified as described previously^53^. Protein concentrations were determined by Bradford assay.

### QconCAT purification

QconCAT halves were expressed in BL21(DE3) cells in M9 medium with ^15^NH_4_Cl and ampicillin^54^. A pre-culture was diluted 1:100 to a 50-mL expression culture. Protein expression was induced at OD_600_ = 0.5 with 1 mM IPTG and cells were grown for 3 h at 37 °C. Cells were harvested by centrifugation and the pellet was dissolved in 1 mL B-PER. 10 µL of 10 mg mL^–1^ lysozyme and 10 µL of DNaseI (ThermoScientific, 1 U µL^–1^) were added and the sample was incubated for 10 min at room temperature. The lysate was centrifuged for 20 min at 16,000*g* and the pellet was resuspended in 2 mL of a 1:10 dilution of B-PER in MilliQ water. The sample was twice again centrifuged and the pellet was resuspended in 2 mL of 1:10 diluted B-PER and centrifuged again. The pellet was resuspended in 600 µL of 10 mM Tris–HCl pH 8.0, 6 M guanidinium chloride and incubated at room temperature for 30 min. After spinning down the unsolubilized protein content the supernatant was loaded onto an equilibrated mini NiNTA spin column and the flow-through was reloaded twice to maximize protein binding. The column was washed twice with 600 µL of 10 mM Tris–HCl pH 6.3, 8 M urea and the QconCAT was eluted with 3 × 200 µL of 10 mM Tris–HCl pH 4.5, 8 M urea, and 400 mM imidazole. The eluate was dialyzed overnight and for additional 4 h against 10 mM Tris–HCl pH 8.0 and 100 mM KCl using a 10-kDa cut-off slide-a-lyzer cassette (ThermoScientific). Purification of the full-length QconCAT was carried out following the same protocol except for expression in LB medium.

### tRNA deaminoacylation

50 µL of 15 mg mL^–1^ tRNA solution (Roche) was mixed with 300 µL of 1 M HCl, vortexed and incubated at room temperature for 15 min. A solution consisting of 300 µL of 1 M NaOH, 60 µL of 3 M sodium acetate, and 1.8 mL of ice-cold ethanol was added. After vortexing the solution was incubated at − 80 °C for 1 h and the tRNA pool was pelleted by centrifugation using a table-top centrifuge (5415R, Eppendorf) at maximum speed. The pellet was washed with ice-cold 75% ethanol, air-dried, and re-dissolved in MilliQ water.

### PURE system reactions

PURExpress was purchased from New England Biolabs and PURE*frex*2.0 from GeneFrontier Corporation (Japan). Enzyme and ribosome solutions (PURE*frex*2.0) or solution B (PURExpress) were mixed either with their respective commercial feeding solution (solution I for PURE*frex*2.0, solution A for PURExpress) according to the supplier’s recommendations or with an equimolar volume of a homemade buffer consisting of 20 mM HEPES–KOH pH 7.6, 180 mM potassium glutamate, 14 mM magnesium acetate, 2 mM DTT, 2 mM spermidine, 100 mM creatine phosphate, 0.1 mg mL^–1^ 10-formyl-tetrahydrofolate (prepared from 5-formyl-tetrahydrofolate according to the protocol described in Ref.^55^), 3 mM ATP, 3 mM GTP, 1 mM UTP, 1 mM CTP, 0.75 mg mL^–1^ deaminoacylated tRNA, 1.35 mg mL^–1^ amino acid mix (^15^N-labeled amino acid mix was from Cambridge Isotope Laboratories; ^14^N amino acid mix for control reactions contained equimolar amounts of all amino acids). Plasmid DNA was added to a final concentration of 5 ng µL^–1^.

### Trypsin digest

Enzymatic digestion of proteins was performed as previously described^54^. Per LC–MS injection, 1.5 µL of PURE system reaction was mixed with 3 µL of 100 mM Tris–HCl pH8.0, 0.3 µL of 20 mM CaCl_2_, and 0.97 µL MilliQ water. Samples were incubated at 90 °C for 10 min and after cooling to room temperature 0.22 µL of 1 mg mL^–1^ trypsin (trypsin-ultra, MS-grade, New England Biolabs) was added. Samples were then incubated at 37 °C overnight. After addition of 0.6 µL 10% trifluoroacetic acid samples were centrifuged in a table-top centrifuge (5415R, Eppendorf) for 10 min at maximum speed. The supernatant was transferred to a glass vial with small-volume insert for LC–MS/MS analysis. For absolute quantitative proteomic analysis three different concentrations of PURE*frex*2.0 and PURExpress samples were mixed with a fixed concentration of both QconCAT halves. Samples were digested with trypsin as described above and, before LC–MS/MS analysis, they were supplemented with 110 nM of ^13^C-Arg/Lys labeled SILs (Pepscan presto, Lelystad, The Netherlands) corresponding to the two quantification peptides on the QconCAT halves.

### Proteomic analysis

LC–MS/MS analysis was performed on a 6460 Triple Quad LCMS system (Agilent Technologies, USA) using Skyline software^14^. 5.5 µL of sample was injected per run to an ACQUITY UPLC Peptide CSH C18 Column (Waters Corporation, USA). The peptides were separated in a gradient of buffer A (25 mM formic acid in MilliQ water) and buffer B (50 mM formic acid in acetonitrile) at a flow rate of 500 µL per minute and at a column temperature of 40 °C. The column was equilibrated with 98% A. After injection, the gradient was changed linearly over 20 min to 70% buffer A, over the next 4 min to 60% buffer A, and over the next 30 s to 20% buffer A. This ratio was held for another 30 s and the column was finally flushed with 98% buffer A to equilibrate for the next run. Selected peptides were measured by multiple reaction monitoring (MRM). For reactions with expression of pTFM1 measurements were split over three LC–MS/MS runs (Supplementary Tables 2, 3, 4). For reactions including ^15^N-labeled amino acids, transitions for peptides containing ^15^N-amino acids were monitored, except for glutamate because of the excess of the light glutamate contained in the buffer.

### Kinetic model

Timeseries data were fitted to the equation $f\left(t\right)=a+b\times {t}^{c}/\left({t}^{c}+{d}^{c}\right)$*,* where *t* denotes time and $f\left(t\right)$ describes the peptide concentration at time *t*. The expression timespan is calculated from the fitted parameters as $2d/c+d$.

## Supplementary Information

Supplementary Information 1.

Supplementary Information 2.

## Data Availability

All data reported in the current study are available from the corresponding author upon reasonable request. This also includes the original .nd file (created in Mathematica version 11.3, Wolfram Research) used to generate the data displayed in the Supplementary Data file.
