# Supplementary material for: In vitro synthesis of 32 translation-factor proteins from a single template reveals impaired ribosomal processivity
Source: Sci Rep. 2021 Jan 21;11:1898. doi: 10.1038/s41598-020-80827-8 (PMC7820420; doi:10.1038/s41598-020-80827-8)
Supplement: Supplementary file 1 — Supplementary Information 1. [file 41598_2020_80827_MOESM1_ESM.pdf]

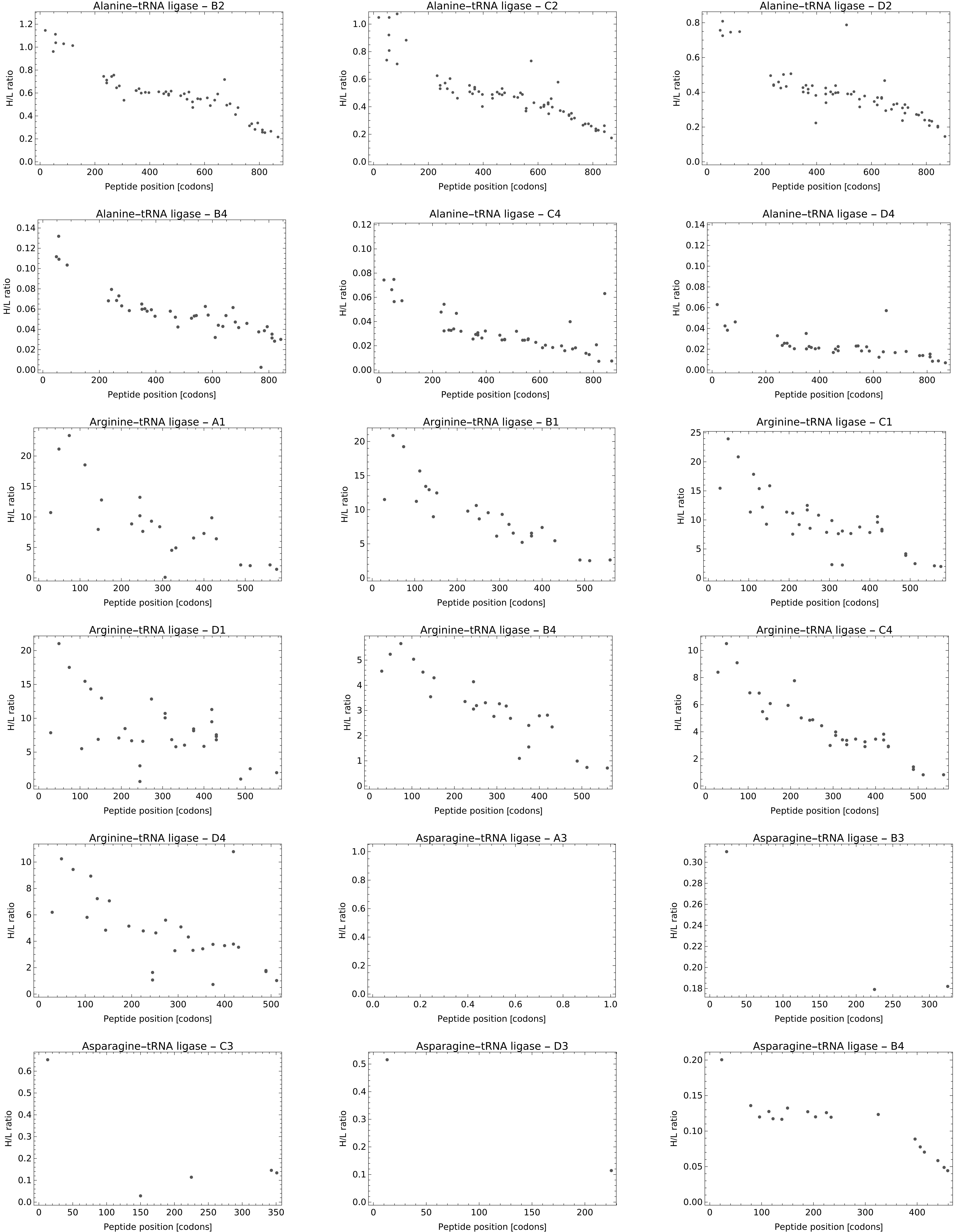
Alanine–tRNA ligase – B2
Alanine–tRNA ligase – C2
Alanine–tRNA ligase – D2
Alanine–tRNA ligase – B4
Alanine–tRNA ligase – C4
Alanine–tRNA ligase – D4
Arginine–tRNA ligase – A1
Arginine–tRNA ligase – B1
Arginine–tRNA ligase – C1
Arginine–tRNA ligase – D1
Arginine–tRNA ligase – B4
Arginine–tRNA ligase – C4
Arginine–tRNA ligase – D4
Asparagine–tRNA ligase – A3
Asparagine–tRNA ligase – B3
Asparagine–tRNA ligase – C3
Asparagine–tRNA ligase – D3
Asparagine–tRNA ligase – B4
H/L ratio
Peptide position [codons]

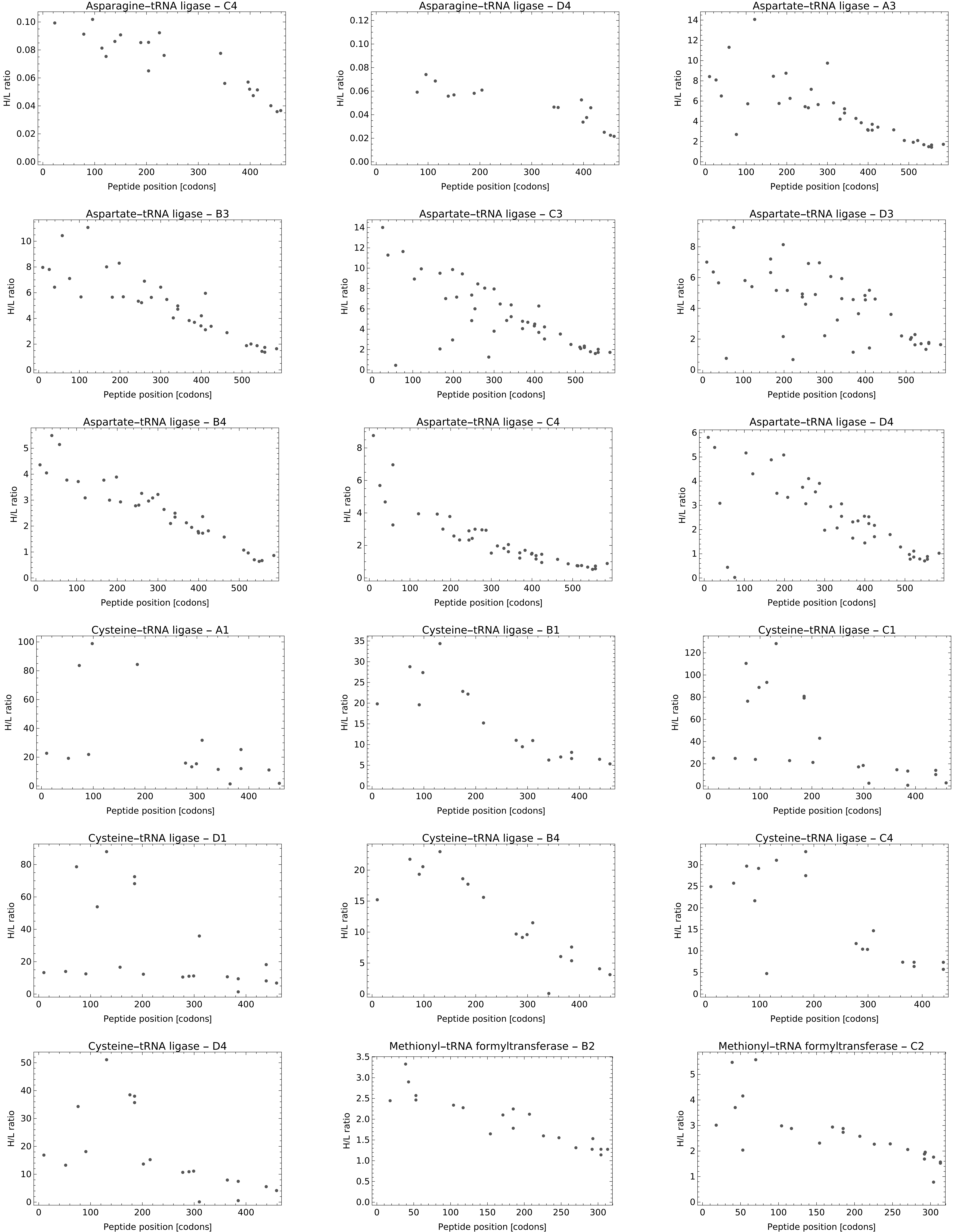
Asparagine–tRNA ligase – C4
Asparagine–tRNA ligase – D4
Aspartate–tRNA ligase – A3
Aspartate–tRNA ligase – B3
Aspartate–tRNA ligase – C3
Aspartate–tRNA ligase – D3
Aspartate–tRNA ligase – B4
Aspartate–tRNA ligase – C4
Aspartate–tRNA ligase – D4
Cysteine–tRNA ligase – A1
Cysteine–tRNA ligase – B1
Cysteine–tRNA ligase – C1
Cysteine–tRNA ligase – D1
Cysteine–tRNA ligase – B4
Cysteine–tRNA ligase – C4
Cysteine–tRNA ligase – D4
Methionyl–tRNA formyltransferase – B2
Methionyl–tRNA formyltransferase – C2
H/L ratio
Peptide position [codons]

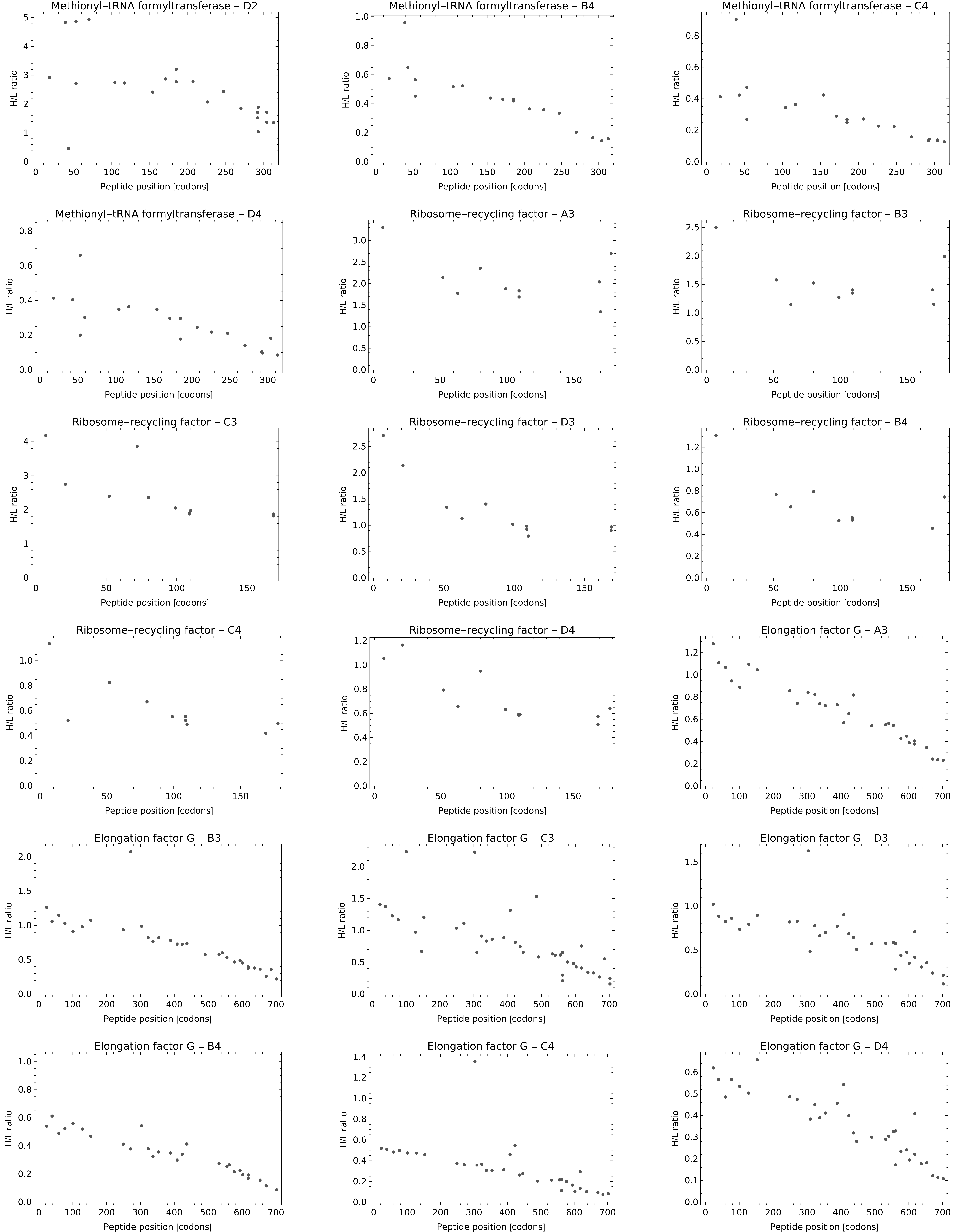
Methionyl–tRNA formyltransferase – D2
Methionyl–tRNA formyltransferase – B4
Methionyl–tRNA formyltransferase – C4
Methionyl–tRNA formyltransferase – D4
Ribosome–recycling factor – A3
Ribosome–recycling factor – B3
Ribosome–recycling factor – C3
Ribosome–recycling factor – D3
Ribosome–recycling factor – B4
Ribosome–recycling factor – C4
Ribosome–recycling factor – D4
Elongation factor G – A3
Elongation factor G – B3
Elongation factor G – C3
Elongation factor G – D3
Elongation factor G – B4
Elongation factor G – C4
Elongation factor G – D4
H/L ratio
Peptide position [codons]

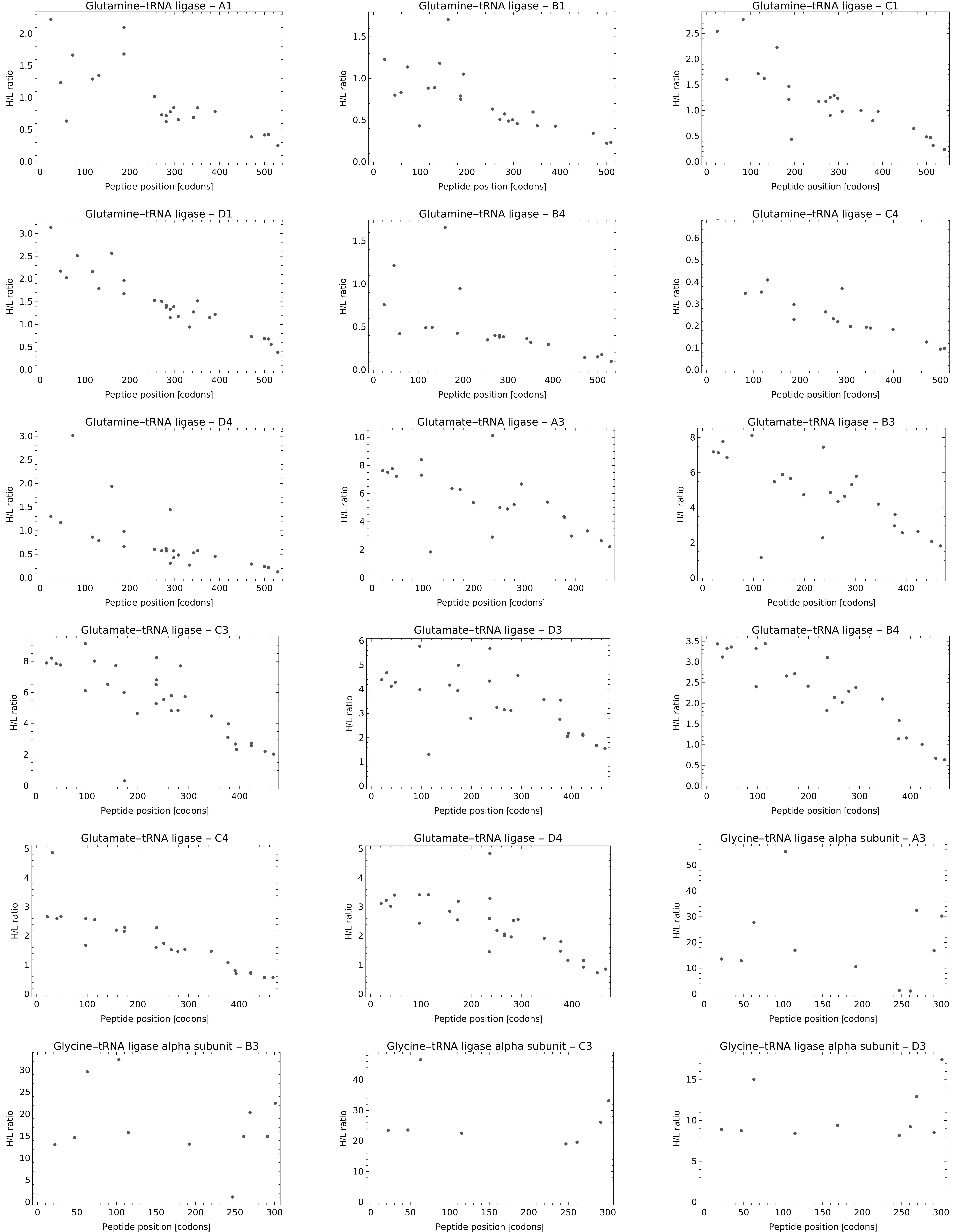
Glutamine–tRNA ligase – A1
Glutamine–tRNA ligase – B1
Glutamine–tRNA ligase – C1
Glutamine–tRNA ligase – D1
Glutamine–tRNA ligase – B4
Glutamine–tRNA ligase – C4
Glutamine–tRNA ligase – D4
Glutamate–tRNA ligase – A3
Glutamate–tRNA ligase – B3
Glutamate–tRNA ligase – C3
Glutamate–tRNA ligase – D3
Glutamate–tRNA ligase – B4
Glutamate–tRNA ligase – C4
Glutamate–tRNA ligase – D4
Glycine–tRNA ligase alpha subunit – A3
Glycine–tRNA ligase alpha subunit – B3
Glycine–tRNA ligase alpha subunit – C3
Glycine–tRNA ligase alpha subunit – D3
H/L ratio
Peptide position [codons]

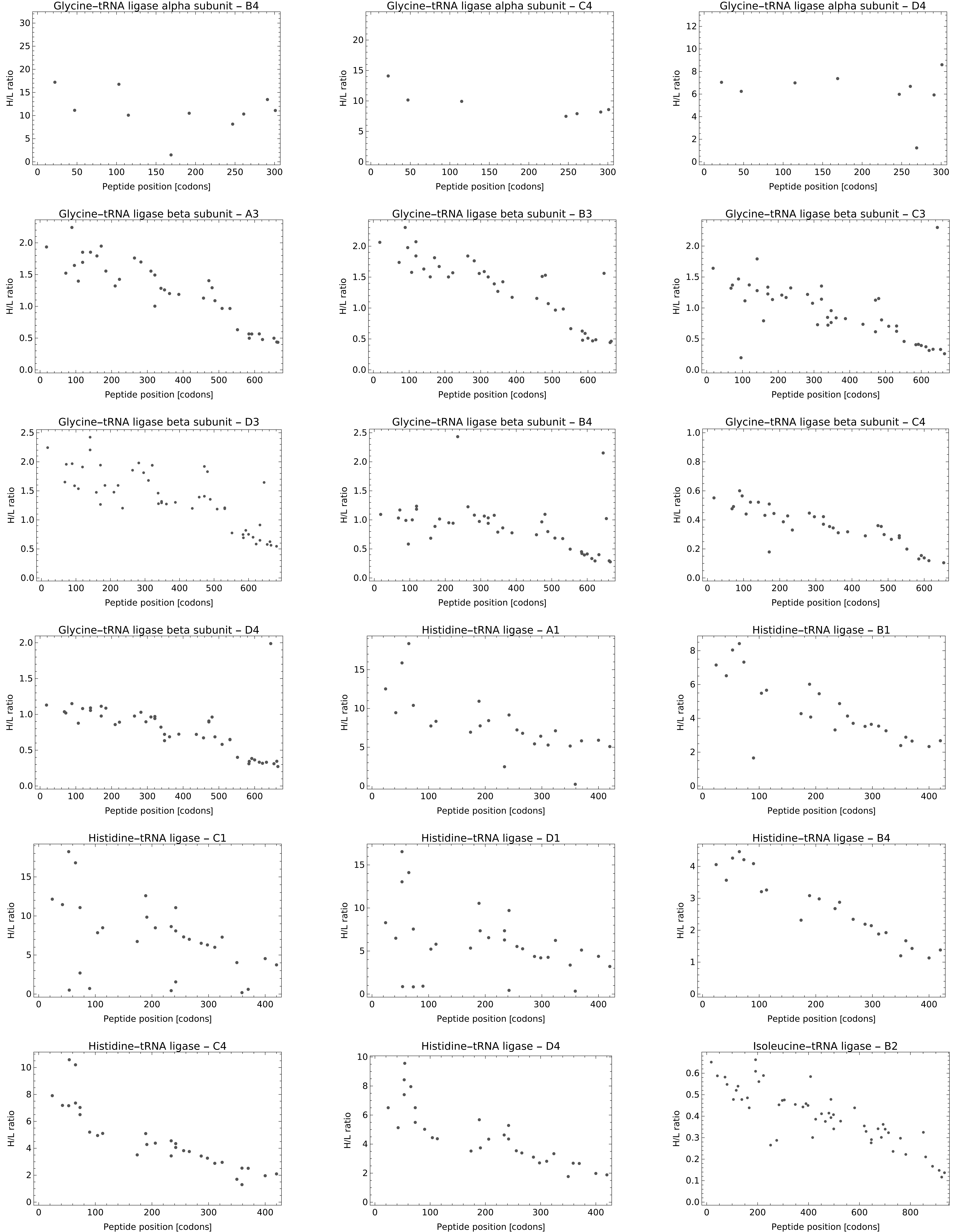
Glycine–tRNA ligase alpha subunit – B4
Glycine–tRNA ligase alpha subunit – C4
Glycine–tRNA ligase alpha subunit – D4
Glycine–tRNA ligase beta subunit – A3
Glycine–tRNA ligase beta subunit – B3
Glycine–tRNA ligase beta subunit – C3
Glycine–tRNA ligase beta subunit – D3
Glycine–tRNA ligase beta subunit – B4
Glycine–tRNA ligase beta subunit – C4
Glycine–tRNA ligase beta subunit – D4
Histidine–tRNA ligase – A1
Histidine–tRNA ligase – B1
Histidine–tRNA ligase – C1
Histidine–tRNA ligase – D1
Histidine–tRNA ligase – B4
Histidine–tRNA ligase – C4
Histidine–tRNA ligase – D4
Isoleucine–tRNA ligase – B2
H/L ratio
Peptide position [codons]

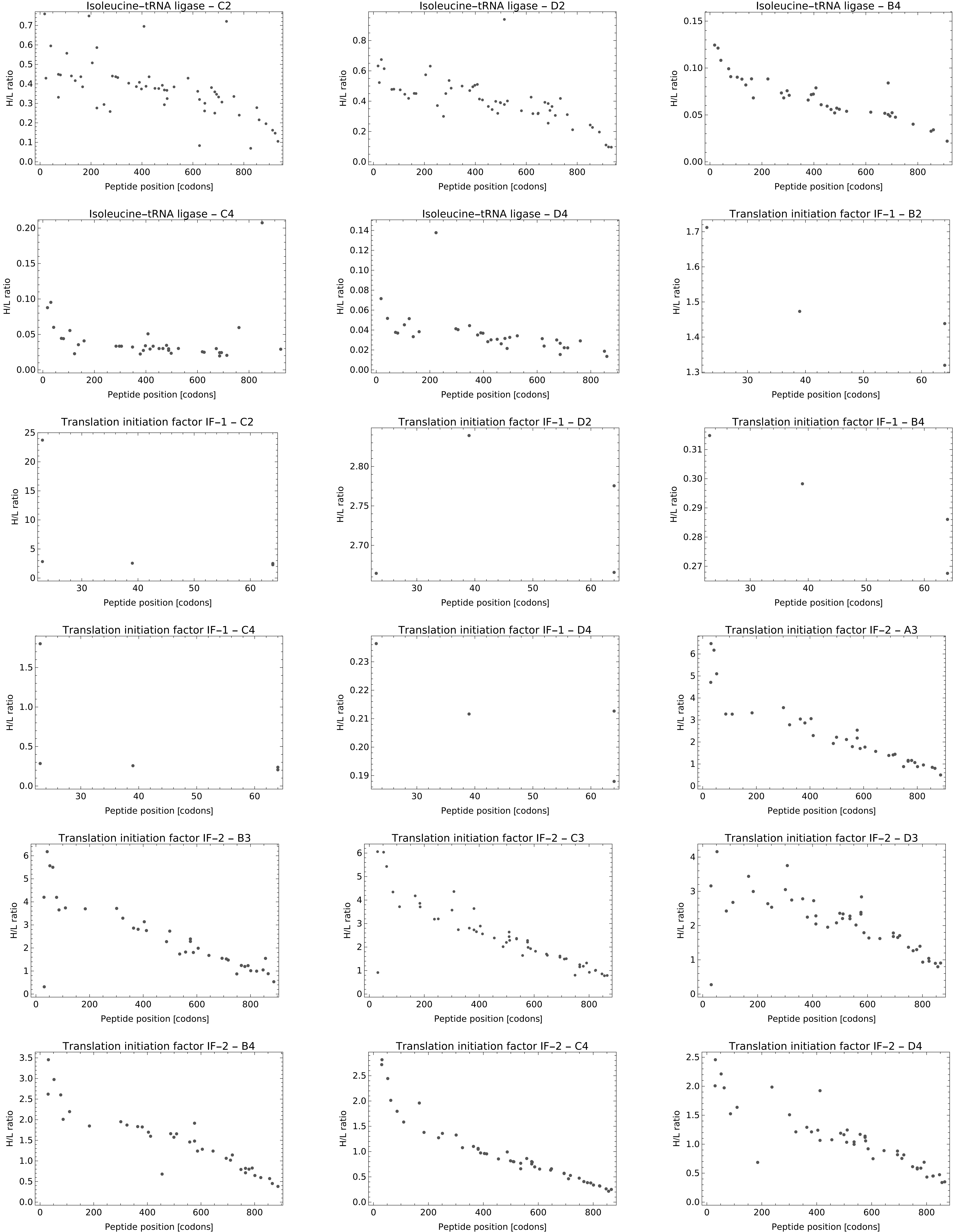
Isoleucine–tRNA ligase – C2
Isoleucine–tRNA ligase – D2
Isoleucine–tRNA ligase – B4
Isoleucine–tRNA ligase – C4
Isoleucine–tRNA ligase – D4
Translation initiation factor IF–1 – B2
Translation initiation factor IF–1 – C2
Translation initiation factor IF–1 – D2
Translation initiation factor IF–1 – B4
Translation initiation factor IF–1 – C4
Translation initiation factor IF–1 – D4
Translation initiation factor IF–2 – A3
Translation initiation factor IF–2 – B3
Translation initiation factor IF–2 – C3
Translation initiation factor IF–2 – D3
Translation initiation factor IF–2 – B4
Translation initiation factor IF–2 – C4
Translation initiation factor IF–2 – D4
H/L ratio
Peptide position [codons]

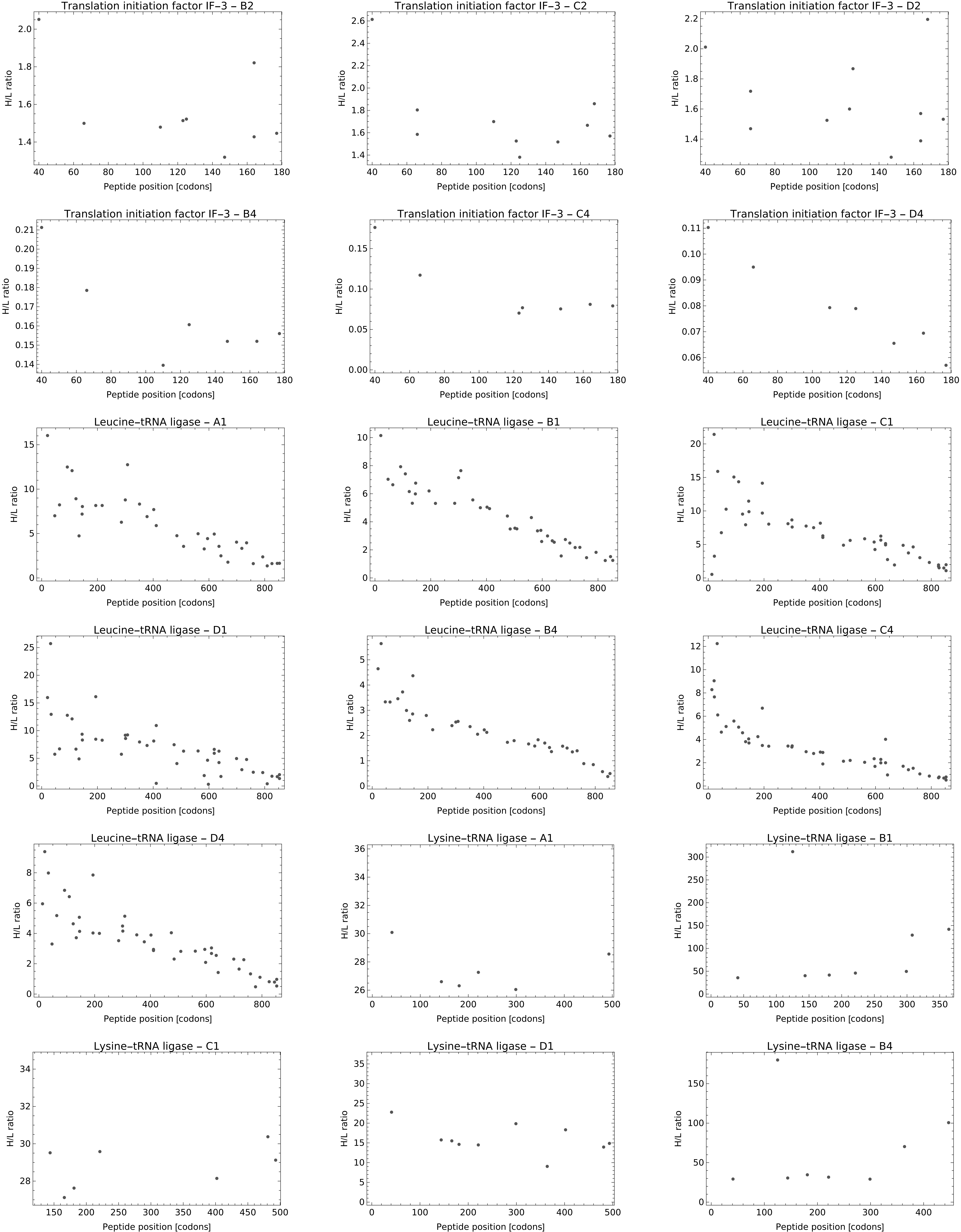
Translation initiation factor IF−3 – B2
Translation initiation factor IF−3 – C2
Translation initiation factor IF−3 – D2
Translation initiation factor IF−3 – B4
Translation initiation factor IF−3 – C4
Translation initiation factor IF−3 – D4
Leucine−tRNA ligase – A1
Leucine−tRNA ligase – B1
Leucine−tRNA ligase – C1
Leucine−tRNA ligase – D1
Leucine−tRNA ligase – B4
Leucine−tRNA ligase – C4
Leucine−tRNA ligase – D4
Lysine−tRNA ligase – A1
Lysine−tRNA ligase – B1
Lysine−tRNA ligase – C1
Lysine−tRNA ligase – D1
Lysine−tRNA ligase – B4
H/L ratio
Peptide position [codons]

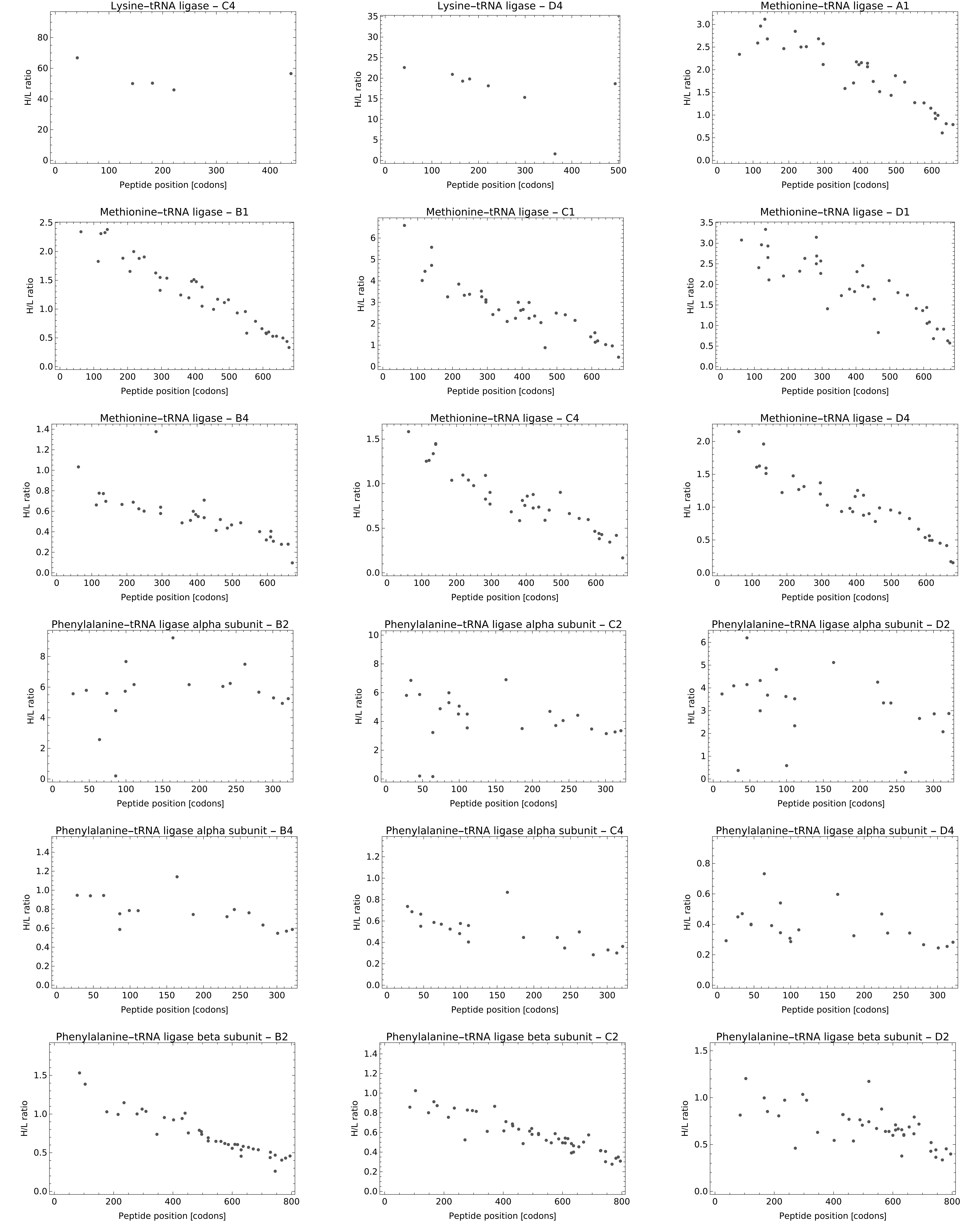
Lysine–tRNA ligase – C4
Lysine–tRNA ligase – D4
Methionine–tRNA ligase – A1
Methionine–tRNA ligase – B1
Methionine–tRNA ligase – C1
Methionine–tRNA ligase – D1
Methionine–tRNA ligase – B4
Methionine–tRNA ligase – C4
Methionine–tRNA ligase – D4
Phenylalanine–tRNA ligase alpha subunit – B2
Phenylalanine–tRNA ligase alpha subunit – C2
Phenylalanine–tRNA ligase alpha subunit – D2
Phenylalanine–tRNA ligase alpha subunit – B4
Phenylalanine–tRNA ligase alpha subunit – C4
Phenylalanine–tRNA ligase alpha subunit – D4
Phenylalanine–tRNA ligase beta subunit – B2
Phenylalanine–tRNA ligase beta subunit – C2
Phenylalanine–tRNA ligase beta subunit – D2
H/L ratio
Peptide position [codons]

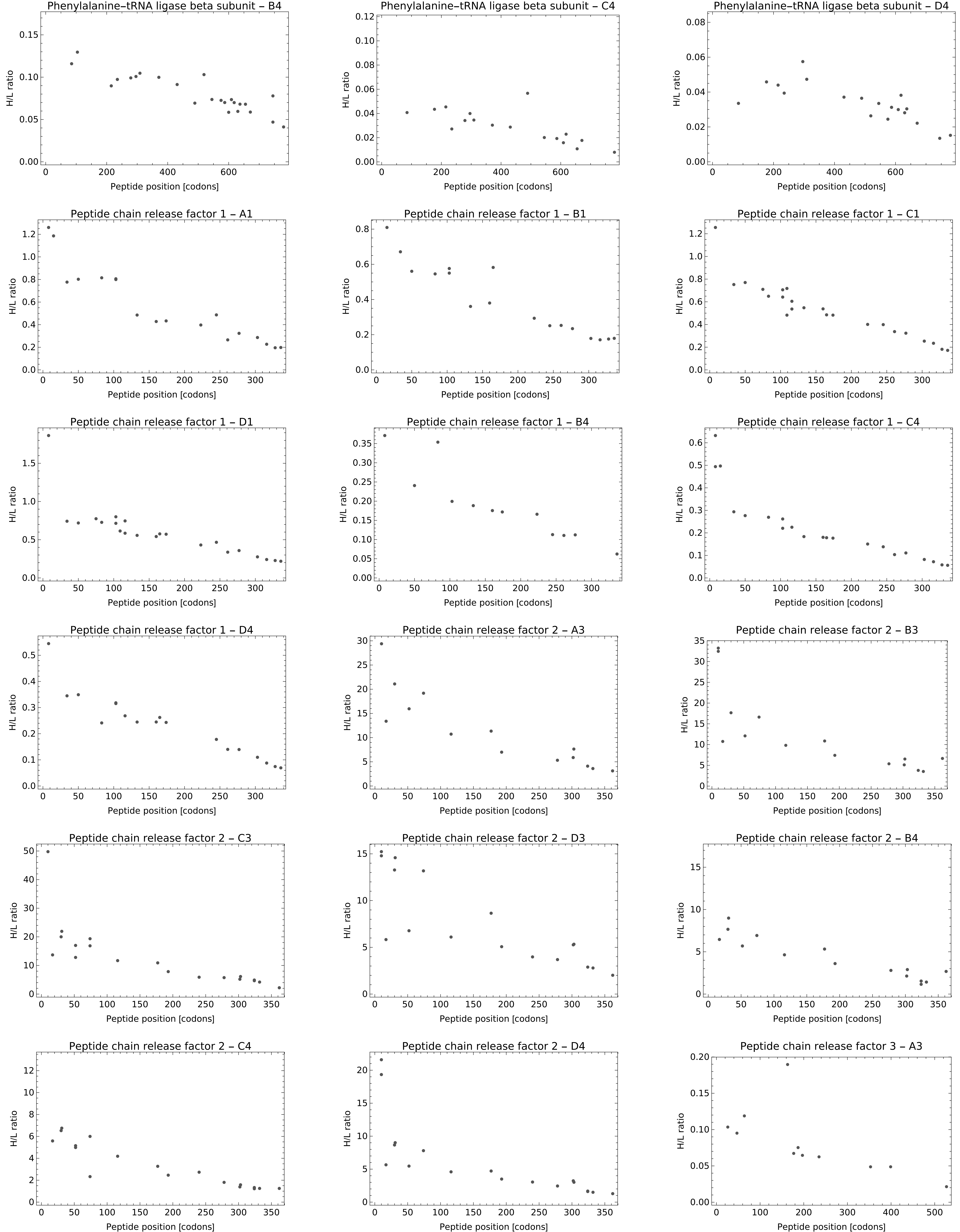
Phenylalanine–tRNA ligase beta subunit – B4
Phenylalanine–tRNA ligase beta subunit – C4
Phenylalanine–tRNA ligase beta subunit – D4
Peptide chain release factor 1 – A1
Peptide chain release factor 1 – B1
Peptide chain release factor 1 – C1
Peptide chain release factor 1 – D1
Peptide chain release factor 1 – B4
Peptide chain release factor 1 – C4
Peptide chain release factor 1 – D4
Peptide chain release factor 2 – A3
Peptide chain release factor 2 – B3
Peptide chain release factor 2 – C3
Peptide chain release factor 2 – D3
Peptide chain release factor 2 – B4
Peptide chain release factor 2 – C4
Peptide chain release factor 2 – D4
Peptide chain release factor 3 – A3
H/L ratio
Peptide position [codons]

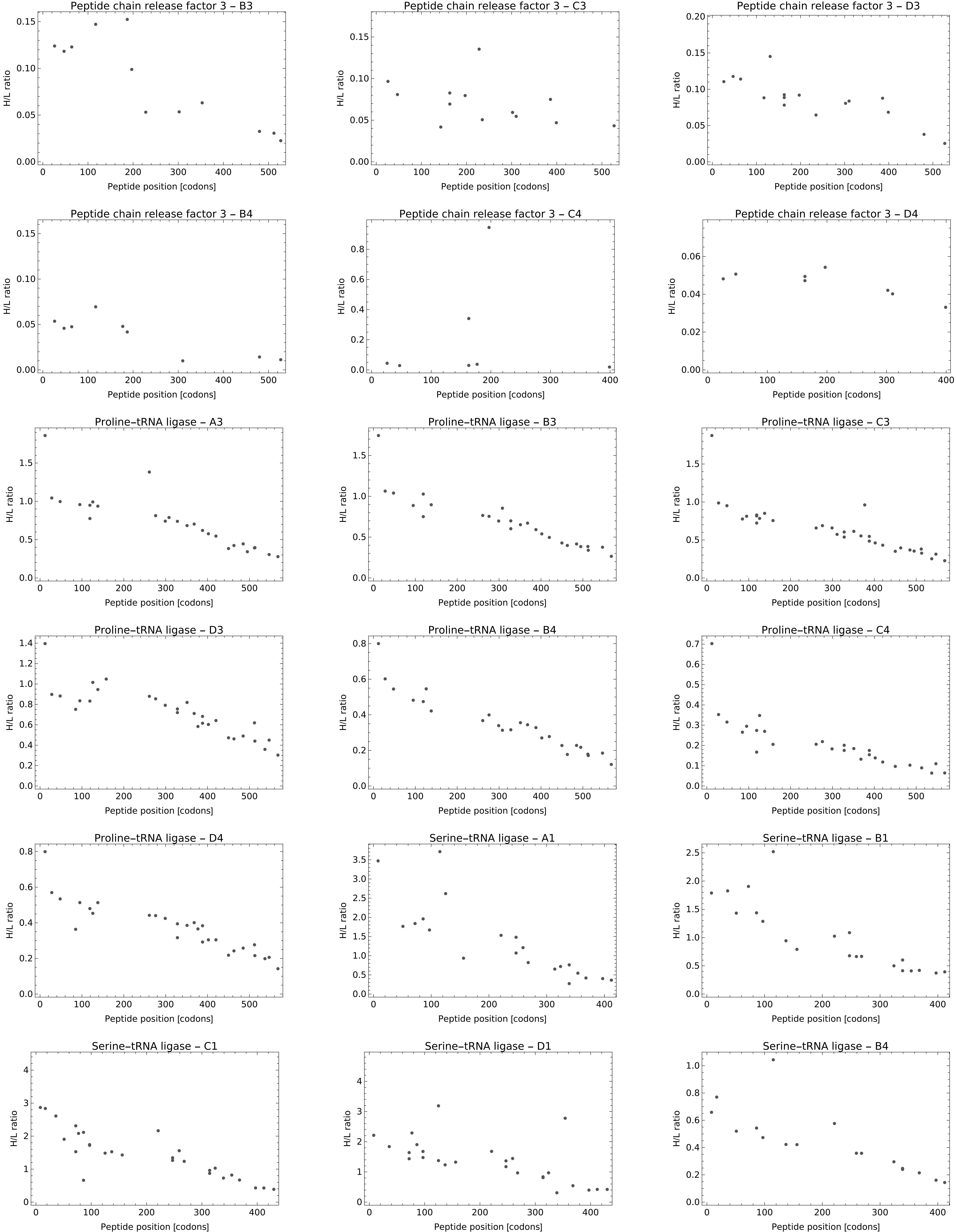
Peptide chain release factor 3 – B3
Peptide chain release factor 3 – C3
Peptide chain release factor 3 – D3
Peptide chain release factor 3 – B4
Peptide chain release factor 3 – C4
Peptide chain release factor 3 – D4
Proline–tRNA ligase – A3
Proline–tRNA ligase – B3
Proline–tRNA ligase – C3
Proline–tRNA ligase – D3
Proline–tRNA ligase – B4
Proline–tRNA ligase – C4
Proline–tRNA ligase – D4
Serine–tRNA ligase – A1
Serine–tRNA ligase – B1
Serine–tRNA ligase – C1
Serine–tRNA ligase – D1
Serine–tRNA ligase – B4
H/L ratio
Peptide position [codons]

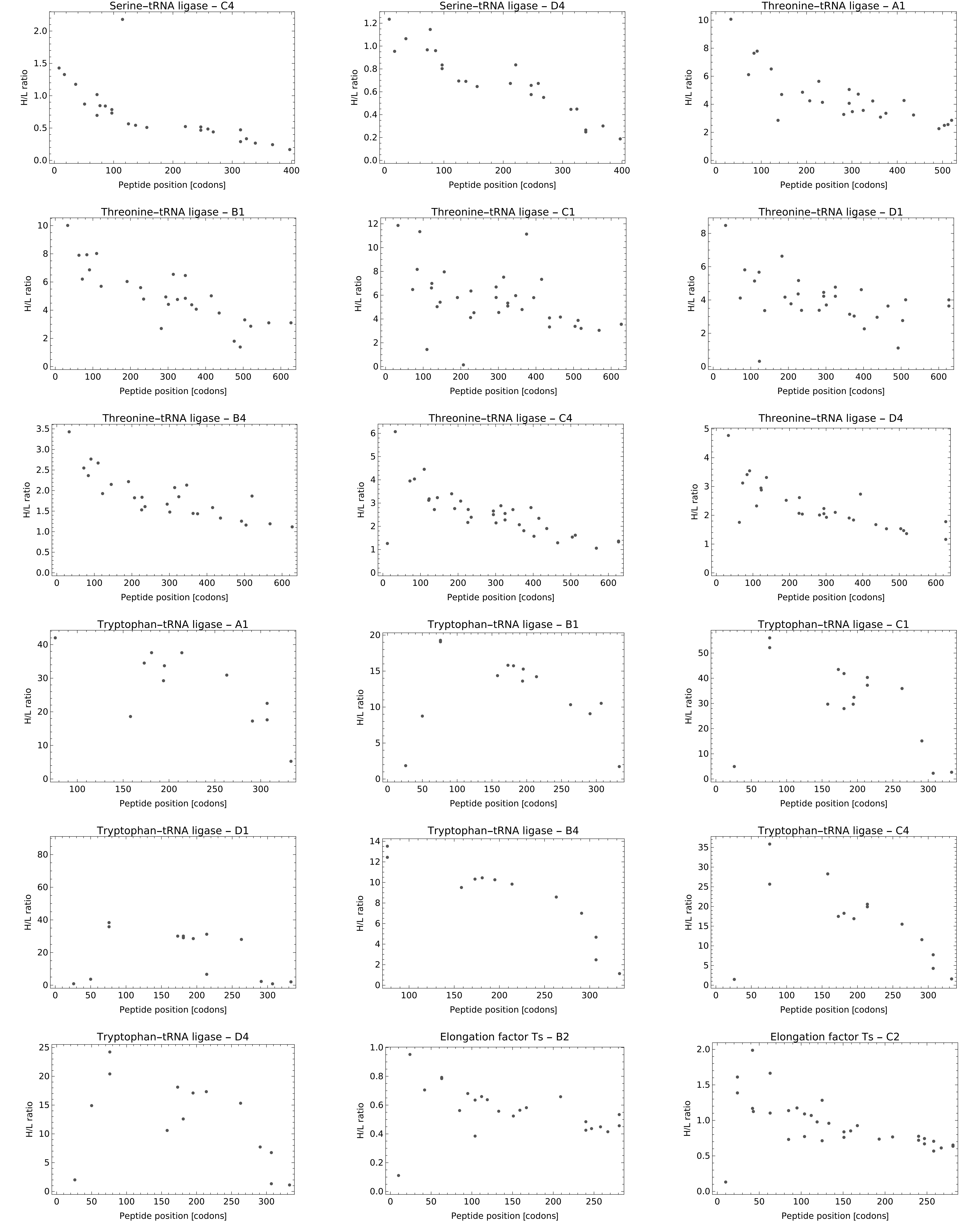
Serine–tRNA ligase – C4
Serine–tRNA ligase – D4
Threonine–tRNA ligase – A1
Threonine–tRNA ligase – B1
Threonine–tRNA ligase – C1
Threonine–tRNA ligase – D1
Threonine–tRNA ligase – B4
Threonine–tRNA ligase – C4
Threonine–tRNA ligase – D4
Tryptophan–tRNA ligase – A1
Tryptophan–tRNA ligase – B1
Tryptophan–tRNA ligase – C1
Tryptophan–tRNA ligase – D1
Tryptophan–tRNA ligase – B4
Tryptophan–tRNA ligase – C4
Tryptophan–tRNA ligase – D4
Elongation factor Ts – B2
Elongation factor Ts – C2
H/L ratio
Peptide position [codons]

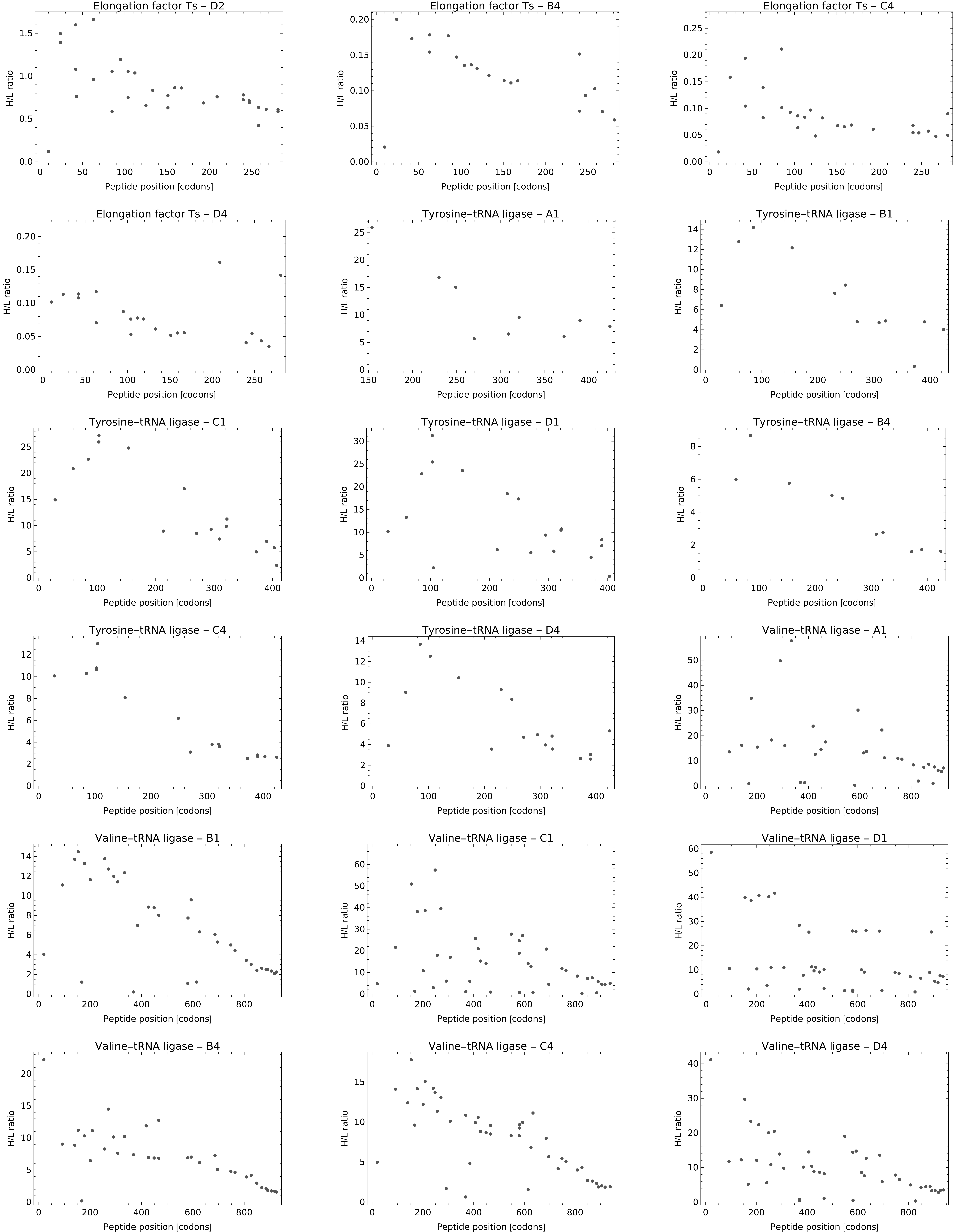

Elongation factor Ts – D2
Elongation factor Ts – B4
Elongation factor Ts – C4
Elongation factor Ts – D4
Tyrosine–tRNA ligase – A1
Tyrosine–tRNA ligase – B1
Tyrosine–tRNA ligase – C1
Tyrosine–tRNA ligase – D1
Tyrosine–tRNA ligase – B4
Tyrosine–tRNA ligase – C4
Tyrosine–tRNA ligase – D4
Valine–tRNA ligase – A1
Valine–tRNA ligase – B1
Valine–tRNA ligase – C1
Valine–tRNA ligase – D1
Valine–tRNA ligase – B4
Valine–tRNA ligase – C4
Valine–tRNA ligase – D4
H/L ratio
Peptide position [codons]
