# Supplementary material for: In vitro synthesis of 32 translation-factor proteins from a single template reveals impaired ribosomal processivity
Source: Sci Rep. 2021 Jan 21;11:1898. doi: 10.1038/s41598-020-80827-8 (PMC7820420; doi:10.1038/s41598-020-80827-8)
Supplement: Supplementary file 2 — Supplementary Information 2. [file 41598_2020_80827_MOESM2_ESM.pdf]

SUPPLEMENTARY INFORMATION

For

In vitro synthesis of 32 translation-factor proteins from a single template reveals impaired ribosomal processivity

Anne Doerr, David Foschepoth, Anthony C. Forster, Christophe Danelon

SUPPLEMENTARY METHODS

QconCAT sequences. Coding sequences are in bold, promoter and terminator sequences are underlined.

QconCAT-N-terminal:

TAATACGACTCACTATAGGGAGACCACAACGGTTTCCCTCTAGAAATAATTTTGTTTAACTTTAAGAAGGAGATATACAT**ATGCGGGGTTCTCATCATCATCATCATCATGGTATGGCTAGCATGACTGGTGGACAGCAAATGGGTCGGGATCTGTACGACGATGACGATAAGGACCTGCAGAGCATCGCGGATTACCCGGTGAAGGTTCTGCTGTTTGGTCAGGCGATTCAAACCGCGGGTACCGTGAAGGGCATCCAGGAGCAACTGAAAGCGCTGGACTTCATTGCGGAGCGTGAATTCGACTTTGAAGTGCGTGATGTTGCGACCCCGGAAGTGGCGGATATTCGTAACAACGTTATCGGCCTGCTGGAAGACCCGAAAACCAGCCTGCTGGATTACATCCGTAGCTATGTTCTGGACGATAGCCGTATCATTTTTGCGGGTACCCCGGACTTTGCGGCGCGTGCGGGTGATATTGCGGCGGCGATTGGTCTGAAGGACCTGGGTCTGACCGATGAGAGCAAAGTGACCGTTGAACTGACCCCGTATGACCTGAGCAAGGCGCAGCAACCGCCGATTCGTATCGGTGTGCTGGTTGCGGCGAAAGTGATTCCGGCGACCATTCTGGGCATCCAGAGCGACCGTGTTAACGCGGATATCGTGAACAAGGTGGCGAACCTGGCGGAGGCGCAACCGGACCGTGCGATTGGTGAGGTGACCGATGTGGTTGAAAAGACCGTTGGCCAGCTGCTGAAAACCATCGCGGAGCTGGTTGAGCAATTTAACCTGCCGATTGAAAAAACCACCCTGACCGCGGCGATCACCACCGTTCTGGCGAAGCTGGCGAACGCGGTGGGTATTGGTGCGGTTAAACTGGCGGACCTGCTGGATGAGGCGCTGGAACGTGCGCAGTACGTGCTGGCGGAGCAAGTTACCCGTCTGACCGGTCTGGAGGGCGAACAGCTGGGTATCGTGAGCCTGCGTTTTCTGCTGGCGAACCTGAACGGCTTCGACCCGGCGAAGTGA**GGCTGCTAACAAAGCCCGAAAGGAAGCTGAGTTGGCTGCTGCCACCGCTGAGCAATAACTAGCATAACCCCTTGGGGCCTCTAAACGGGTCTTGAGGGGTTTTTTG

QconCAT-C-terminal:

TAATACGACTCACTATAGGGAGACCACAACGGTTTCCCTCTAGAAATAATTTTGTTTAACTTTAAGAAGGAGATATACAT**ATGCGGGGTTCTCATCATCATCATCATCATGGTATGGCTAGCATGACTGGTGGACAGCAAATGGGTCGGGATCTGTACGACGATGACGATAAGATTTTTAACACCCTGACCCGTTTTACCCTGGCGGCGCTGGCGAGCACCGGTCGTGATTATCTGGTGCCGAGCCGTGTTTTCGAAATTAACCGTGTGGCGGTTGCGACCATTGGTGCGGTGCTGCCGGGTGACTTTAAGATCGCGACCGATCCGTTCGTTGGTAACCTGACCTTCTTTCGTTGGACCGACGATGACCTGTACGGTATCATTCGTTTTAACGCGCTGTATGGCGAGATTTTCAAACTGCTGGTTAGCGAGCTGAGCGGCGTTGAACCGAAGATCACCGACGTGGAAGTTCTGAAAGCGAGCTTTGTGACCCTGCAGGATGTTGGTGGCCGTATGCATGAGCTGGCGGAAAAAGCGGTGGCGGAGGCGTACTATGCGAGCCGTTTCCTGGAGGAAGGTGACAAGCTGGCGGAACTGGGTCCGCAAGGCCTGATTACCACCCTGAAAATCACCGTGCCGGTTGATGCGACCGAGGAACAGGTTCGTACCTACAACTTCCCGCAAGGTCGTACCTGGGGCGTGCCGATGAGCCTGTTCGTTCACAAATTTTATGCGCTGCCGCAGAGCCCGCAACTGTTTAAGCTGGCGGCGAGCATTGCGTTCAAAATCAACGTTTTTGACCGTACCGGTGATATTGGCCTGTTCCGTGGTTACACCGCGGCGAGCATCCGTGGCCTGGTGGCGCAAGTTACCGACGAGGAAGCGCTGGCGGAGCGTGGTATTACCATCAACACCAGCCACGTGGAATACGATACCCCGACCCGTACCAACCTGATTGGTGCGGTTGCGCGTGACACCATCGGCGATATCATTATCCTGCCGCGTTGCGACTATAAGGATGACGATAAATGA**TCCGGCTGCTAACAAAGCCCGAAAGGAAGCTGAGTTGGCTGCTGCCACCGCTGAGCAATAACTAGCATAACCCCTTGGGGCCTCTAAACGGGTCTTGAGGGGTTTTTTG

SUPPLEMENTARY TABLES

Table S1: Composition of the ^{15}N amino acid mix. Information is as provided by Cambridge Isotope Laboratory for product NLM-2161.

Amino acids	Fraction (%)
Alanine	5.88
Arginine	5.88
Asparagine	5
Aspartic acid	8.4
Cysteine	3
Glutamic acid	8.4
Glutamine	5
Glycine	5.04
Histidine	1.68
Isoleucine	3.36
Leucine	8.4
Lysine	11.76
Methionine	0.84
Phenylalanine	3.36
Proline	5.88
Serine	3.36
Threonine	4.2
Tryptophan	3
Tyrosine	3.36
Valine	4.2

Table S2: Transitions of the MS/MS measurements for the proteins expressed from pTFM1 (injection 1)

Protein	Compound name	Precursor ion *m/z*	Product ion *m/z*	Collision energy (eV)	Cell accelerator voltage (V)	Ret time (min)	Ion name
alanine tRNA ligase	TGDIGLFR.light	439.7402	720.4039	14.6	5	8.96	y6
alanine tRNA ligase	TGDIGLFR.light	439.7402	322.1874	14.6	5	8.96	y2
alanine tRNA ligase	TGDIGLFR.light	439.7402	274.1034	14.6	5	8.96	b3
alanine tRNA ligase	TGDIGLFR.heavy	445.2239	729.3772	14.6	5	8.96	y6
alanine tRNA ligase	TGDIGLFR.heavy	445.2239	327.1725	14.6	5	8.96	y2
alanine tRNA ligase	TGDIGLFR.heavy	445.2239	277.0945	14.6	5	8.96	b3
arginine tRNA ligase	LADLLDEALER.light	629.3379	1073.547	20.5	5	15.2	y9
arginine tRNA ligase	LADLLDEALER.light	629.3379	845.4363	20.5	5	15.2	y7
arginine tRNA ligase	LADLLDEALER.light	629.3379	732.3523	20.5	5	15.2	y6
arginine tRNA ligase	LADLLDEALER.light	629.3379	526.3235	20.5	5	15.2	b5
arginine tRNA ligase	LADLLDEALER.heavy	635.3201	1083.518	20.5	5	15.2	y9
arginine tRNA ligase	LADLLDEALER.heavy	635.3201	853.4126	20.5	5	15.2	y7
arginine tRNA ligase	LADLLDEALER.heavy	635.3201	739.3315	20.5	5	15.2	y6
arginine tRNA ligase	LADLLDEALER.heavy	635.3201	531.3087	20.5	5	15.2	b5
asparagine tRNA ligase	TNLIGAVAR.light	457.7745	473.2831	15.2	5	7.34	y5
asparagine tRNA ligase	TNLIGAVAR.light	457.7745	216.0979	15.2	5	7.34	b2
asparagine tRNA ligase	TNLIGAVAR.light	457.7745	329.1819	15.2	5	7.34	b3
asparagine tRNA ligase	TNLIGAVAR.heavy	464.2553	481.2593	15.2	5	7.34	y5
asparagine tRNA ligase	TNLIGAVAR.heavy	464.2553	219.089	15.2	5	7.34	b2
asparagine tRNA ligase	TNLIGAVAR.heavy	464.2553	333.1701	15.2	5	7.34	b3
asparagine tRNA ligase	VSTLDLENLPR.light	628.8459	856.4523	20.5	5	12.19	y7
asparagine tRNA ligase	VSTLDLENLPR.light	628.8459	272.1717	20.5	5	12.19	y2
asparagine tRNA ligase	VSTLDLENLPR.light	628.8459	187.1077	20.5	5	12.19	b2
asparagine tRNA ligase	VSTLDLENLPR.heavy	635.8251	866.4227	20.5	5	12.19	y7
asparagine tRNA ligase	VSTLDLENLPR.heavy	635.8251	277.1569	20.5	5	12.19	y2
asparagine tRNA ligase	VSTLDLENLPR.heavy	635.8251	189.1018	20.5	5	12.19	b2
glycine tRNA ligase_ beta subunit	VANLAEAQPDR.light	592.3069	786.3741	19.4	5	4.82	y7
glycine tRNA ligase_ beta subunit	VANLAEAQPDR.light	592.3069	715.3369	19.4	5	4.82	y6
glycine tRNA ligase_ beta subunit	VANLAEAQPDR.light	592.3069	387.1987	19.4	5	4.82	y3
glycine tRNA ligase_ beta subunit	VANLAEAQPDR.heavy	599.7847	796.3444	19.4	5	4.82	y7

Table S2 (continued)

Protein	Compound name	Precursor ion *m/z*	Product ion *m/z*	Collision energy (eV)	Cell accelerator voltage (V)	Ret time (min)	Ion name
glycine tRNA ligase_ beta subunit	VANLAEAQPDR.heavy	599.7847	724.3103	19.4	5	4.82	y6
glycine tRNA ligase_ beta subunit	VANLAEAQPDR.heavy	599.7847	393.1809	19.4	5	4.82	y3
glycine tRNA ligase_ beta subunit	VIPATILGIQSDR.light	691.9037	1170.648	22.4	5	13.18	y11
glycine tRNA ligase_ beta subunit	VIPATILGIQSDR.light	691.9037	788.4261	22.4	5	13.18	y7
glycine tRNA ligase_ beta subunit	VIPATILGIQSDR.light	691.9037	675.342	22.4	5	13.18	y6
glycine tRNA ligase_ beta subunit	VIPATILGIQSDR.heavy	700.3785	1185.603	22.4	5	13.18	y11
glycine tRNA ligase_ beta subunit	VIPATILGIQSDR.heavy	700.3785	799.3935	22.4	5	13.18	y7
glycine tRNA ligase_ beta subunit	VIPATILGIQSDR.heavy	700.3785	685.3124	22.4	5	13.18	y6
glycine tRNA ligase_ beta subunit	LADAEFFFNTDR.light	723.3384	946.4417	23.4	5	14.58	y7
glycine tRNA ligase_ beta subunit	LADAEFFFNTDR.light	723.3384	799.3733	23.4	5	14.58	y6
glycine tRNA ligase_ beta subunit	LADAEFFFNTDR.light	723.3384	652.3049	23.4	5	14.58	y5
glycine tRNA ligase_ beta subunit	LADAEFFFNTDR.light	723.3384	185.1285	23.4	5	14.58	b2
glycine tRNA ligase_ beta subunit	LADAEFFFNTDR.heavy	730.8162	957.4091	23.4	5	14.58	y7
glycine tRNA ligase_ beta subunit	LADAEFFFNTDR.heavy	730.8162	809.3437	23.4	5	14.58	y6
glycine tRNA ligase_ beta subunit	LADAEFFFNTDR.heavy	730.8162	661.2782	23.4	5	14.58	y5
glycine tRNA ligase_ beta subunit	LADAEFFFNTDR.heavy	730.8162	187.1225	23.4	5	14.58	b2
leucine tRNA ligase	NWVSPVDAIVER.light	692.8646	1084.6	22.5	5	14.46	y10
leucine tRNA ligase	NWVSPVDAIVER.light	692.8646	985.5313	22.5	5	14.46	y9
leucine tRNA ligase	NWVSPVDAIVER.light	692.8646	898.4993	22.5	5	14.46	y8
leucine tRNA ligase	NWVSPVDAIVER.light	692.8646	400.1979	22.5	5	14.46	b3
leucine tRNA ligase	NWVSPVDAIVER.heavy	700.8409	1096.564	22.5	5	14.46	y10
leucine tRNA ligase	NWVSPVDAIVER.heavy	700.8409	996.4987	22.5	5	14.46	y9
leucine tRNA ligase	NWVSPVDAIVER.heavy	700.8409	908.4696	22.5	5	14.46	y8
leucine tRNA ligase	NWVSPVDAIVER.heavy	700.8409	405.1831	22.5	5	14.46	b3
methionine tRNA ligase	VNADIVNK.light	436.7454	659.3723	14.5	5	2.8	y6
methionine tRNA ligase	VNADIVNK.light	436.7454	360.2241	14.5	5	2.8	y3
methionine tRNA ligase	VNADIVNK.light	436.7454	214.1186	14.5	5	2.8	b2
methionine tRNA ligase	VNADIVNK.heavy	442.2291	667.3485	14.5	5	2.8	y6
methionine tRNA ligase	VNADIVNK.heavy	442.2291	365.2093	14.5	5	2.8	y3

Table S2 (continued)

Protein	Compound name	Precursor ion *m/z*	Product ion *m/z*	Collision energy (eV)	Cell accelerator voltage (V)	Ret time (min)	Ion name
methionine tRNA ligase	VNADIVNK.heavy	442.2291	217.1097	14.5	5	2.8	b2
phenylalanine tRNA ligase_ alpha subunit	AQQPPIR.light	405.235	610.3671	13.6	5	1.73	y5
phenylalanine tRNA ligase_ alpha subunit	AQQPPIR.light	405.235	482.3085	13.6	5	1.73	y4
phenylalanine tRNA ligase_ alpha subunit	AQQPPIR.light	405.235	328.1615	13.6	5	1.73	b3
phenylalanine tRNA ligase_ alpha subunit	AQQPPIR.heavy	411.2173	619.3404	13.6	5	1.73	y5
phenylalanine tRNA ligase_ alpha subunit	AQQPPIR.heavy	411.2173	489.2878	13.6	5	1.73	y4
phenylalanine tRNA ligase_ alpha subunit	AQQPPIR.heavy	411.2173	333.1467	13.6	5	1.73	b3
phenylalanine tRNA ligase_ beta subunit	VAVATIGAVLPGDFK.light	729.4218	563.2824	23.6	5	15.59	y5
phenylalanine tRNA ligase_ beta subunit	VAVATIGAVLPGDFK.light	729.4218	294.1812	23.6	5	15.59	y2
phenylalanine tRNA ligase_ beta subunit	VAVATIGAVLPGDFK.light	729.4218	270.1812	23.6	5	15.59	b3
phenylalanine tRNA ligase_ beta subunit	VAVATIGAVLPGDFK.heavy	737.398	569.2646	23.6	5	15.59	y5
phenylalanine tRNA ligase_ beta subunit	VAVATIGAVLPGDFK.heavy	737.398	297.1723	23.6	5	15.59	y2
phenylalanine tRNA ligase_ beta subunit	VAVATIGAVLPGDFK.heavy	737.398	273.1723	23.6	5	15.59	b3
proline tRNA ligase	TIAELVEQFNLPIEK.light	872.48	486.2922	28	5	20.03	y4
proline tRNA ligase	TIAELVEQFNLPIEK.light	872.48	215.139	28	5	20.03	b2
proline tRNA ligase	TIAELVEQFNLPIEK.light	872.48	415.2187	28	5	20.03	b4
proline tRNA ligase	TIAELVEQFNLPIEK.heavy	879.9578	490.2804	28	5	20.03	y4
proline tRNA ligase	TIAELVEQFNLPIEK.heavy	879.9578	217.1331	28	5	20.03	b2
proline tRNA ligase	TIAELVEQFNLPIEK.heavy	879.9578	418.2098	28	5	20.03	b4
proline tRNA ligase	DVATPEVADIR.light	593.3091	799.4308	19.4	5	9.46	y7
proline tRNA ligase	DVATPEVADIR.light	593.3091	215.1026	19.4	5	9.46	b2
proline tRNA ligase	DVATPEVADIR.light	593.3091	387.1874	19.4	5	9.46	b4
proline tRNA ligase	DVATPEVADIR.heavy	599.7899	808.4042	19.4	5	9.46	y7
proline tRNA ligase	DVATPEVADIR.heavy	599.7899	217.0967	19.4	5	9.46	b2
proline tRNA ligase	DVATPEVADIR.heavy	599.7899	391.1756	19.4	5	9.46	b4
serine tRNA ligase	GEDIEPLR.light	464.7404	742.4094	15.4	5	5.89	y6
serine tRNA ligase	GEDIEPLR.light	464.7404	514.2984	15.4	5	5.89	y4
serine tRNA ligase	GEDIEPLR.light	464.7404	385.2558	15.4	5	5.89	y3
serine tRNA ligase	GEDIEPLR.heavy	469.227	750.3857	15.4	5	5.89	y6
serine tRNA ligase	GEDIEPLR.heavy	469.227	520.2806	15.4	5	5.89	y4
serine tRNA ligase	GEDIEPLR.heavy	469.227	391.238	15.4	5	5.89	y3
serine tRNA ligase	LGEELDAAK.light	473.248	832.4047	15.7	5	5.49	y8
serine tRNA ligase	LGEELDAAK.light	473.248	775.3832	15.7	5	5.49	y7

Table S2 (continued)

Protein	Compound name	Precursor ion *m/z*	Product ion *m/z*	Collision energy (eV)	Cell accelerator voltage (V)	Ret time (min)	Ion name
serine tRNA ligase	LGEELDAAK.light	473.248	646.3406	15.7	5	5.49	y6
serine tRNA ligase	LGEELDAAK.light	473.248	517.298	15.7	5	5.49	y5
serine tRNA ligase	LGEELDAAK.heavy	477.2362	839.3839	15.7	5	5.49	y8
serine tRNA ligase	LGEELDAAK.heavy	477.2362	781.3654	15.7	5	5.49	y7
serine tRNA ligase	LGEELDAAK.heavy	477.2362	652.3228	15.7	5	5.49	y6
serine tRNA ligase	LGEELDAAK.heavy	477.2362	523.2802	15.7	5	5.49	y5
serine tRNA ligase	EFDFEVR.light	471.2218	665.3253	15.6	5	10.32	y5
serine tRNA ligase	EFDFEVR.light	471.2218	550.2984	15.6	5	10.32	y4
serine tRNA ligase	EFDFEVR.light	471.2218	403.23	15.6	5	10.32	y3
serine tRNA ligase	EFDFEVR.light	471.2218	274.1874	15.6	5	10.32	y2
serine tRNA ligase	EFDFEVR.heavy	475.2099	672.3046	15.6	5	10.32	y5
serine tRNA ligase	EFDFEVR.heavy	475.2099	556.2806	15.6	5	10.32	y4
serine tRNA ligase	EFDFEVR.heavy	475.2099	408.2151	15.6	5	10.32	y3
serine tRNA ligase	EFDFEVR.heavy	475.2099	279.1725	15.6	5	10.32	y2
tryptophan tRNA ligase	FNALYGEIFK.light	601.3162	940.5138	19.6	5	14.59	y8
tryptophan tRNA ligase	FNALYGEIFK.light	601.3162	869.4767	19.6	5	14.59	y7
tryptophan tRNA ligase	FNALYGEIFK.light	601.3162	756.3927	19.6	5	14.59	y6
tryptophan tRNA ligase	FNALYGEIFK.light	601.3162	593.3293	19.6	5	14.59	y5
tryptophan tRNA ligase	FNALYGEIFK.heavy	606.7999	948.4901	19.6	5	14.59	y8
tryptophan tRNA ligase	FNALYGEIFK.heavy	606.7999	876.456	19.6	5	14.59	y7
tryptophan tRNA ligase	FNALYGEIFK.heavy	606.7999	762.3749	19.6	5	14.59	y6
tryptophan tRNA ligase	FNALYGEIFK.heavy	606.7999	598.3145	19.6	5	14.59	y5
valine tRNA ligase	YVILPLVNR.light	543.8371	824.5352	17.9	5	13.61	y7
valine tRNA ligase	YVILPLVNR.light	543.8371	711.4512	17.9	5	13.61	y6
valine tRNA ligase	YVILPLVNR.light	543.8371	598.3671	17.9	5	13.61	y5
valine tRNA ligase	YVILPLVNR.heavy	550.3179	835.5026	17.9	5	13.61	y7
valine tRNA ligase	YVILPLVNR.heavy	550.3179	721.4215	17.9	5	13.61	y6
valine tRNA ligase	YVILPLVNR.heavy	550.3179	607.3404	17.9	5	13.61	y5
valine tRNA ligase	FTLAALASTGR.light	554.3115	604.3413	18.2	5	12.13	y6
valine tRNA ligase	FTLAALASTGR.light	554.3115	491.2572	18.2	5	12.13	y5
valine tRNA ligase	FTLAALASTGR.light	554.3115	420.2201	18.2	5	12.13	y4
valine tRNA ligase	FTLAALASTGR.heavy	561.2907	613.3146	18.2	5	12.13	y6
valine tRNA ligase	FTLAALASTGR.heavy	561.2907	499.2335	18.2	5	12.13	y5
valine tRNA ligase	FTLAALASTGR.heavy	561.2907	427.1994	18.2	5	12.13	y4
EF-G	IATDPFVGNLTFFR.light	799.4223	1197.642	25.8	5	18.87	y10

Table S2 (continued)

Protein	Compound name	Precursor ion *m/z*	Product ion *m/z*	Collision energy (eV)	Cell accelerator voltage (V)	Ret time (min)	Ion name
EF-G	IATDPFVGNLTFFR.light	799.4223	854.4519	25.8	5	18.87	y7
EF-G	IATDPFVGNLTFFR.light	799.4223	185.1285	25.8	5	18.87	b2
EF-G	IATDPFVGNLTFFR.light	799.4223	401.2031	25.8	5	18.87	b4
EF-G	IATDPFVGNLTFFR.light	799.4223	401.2107	25.8	5	18.87	b8
EF-G	IATDPFVGNLTFFR.heavy	808.3956	1211.6	25.8	5	18.87	y10
EF-G	IATDPFVGNLTFFR.heavy	808.3956	865.4193	25.8	5	18.87	y7
EF-G	IATDPFVGNLTFFR.heavy	808.3956	187.1225	25.8	5	18.87	b2
EF-G	IATDPFVGNLTFFR.heavy	808.3956	405.1912	25.8	5	18.87	b4
EF-G	IATDPFVGNLTFFR.heavy	808.3956	405.1988	25.8	5	18.87	b8
EF-G	AGDIAAAIGLK.light	500.2953	643.4137	16.5	5	11.26	y7
EF-G	AGDIAAAIGLK.light	500.2953	572.3766	16.5	5	11.26	y6
EF-G	AGDIAAAIGLK.light	500.2953	129.0659	16.5	5	11.26	b2
EF-G	AGDIAAAIGLK.light	500.2953	244.0928	16.5	5	11.26	b3
EF-G	AGDIAAAIGLK.heavy	506.2775	651.39	16.5	5	11.26	y7
EF-G	AGDIAAAIGLK.heavy	506.2775	579.3559	16.5	5	11.26	y6
EF-G	AGDIAAAIGLK.heavy	506.2775	131.0599	16.5	5	11.26	b2
EF-G	AGDIAAAIGLK.heavy	506.2775	247.0839	16.5	5	11.26	b3
EF-G	GIQEQLK.light	408.2347	645.3566	13.7	5	3.46	y5
EF-G	GIQEQLK.light	408.2347	517.298	13.7	5	3.46	y4
EF-G	GIQEQLK.light	408.2347	171.1128	13.7	5	3.46	b2
EF-G	GIQEQLK.heavy	412.7214	652.3359	13.7	5	3.46	y5
EF-G	GIQEQLK.heavy	412.7214	522.2832	13.7	5	3.46	y4
EF-G	GIQEQLK.heavy	412.7214	173.1069	13.7	5	3.46	b2
EF-G	LAASIAFK.light	410.75	636.3715	13.7	5	8.05	y6
EF-G	LAASIAFK.light	410.75	565.3344	13.7	5	8.05	y5
EF-G	LAASIAFK.light	410.75	185.1285	13.7	5	8.05	b2
EF-G	LAASIAFK.heavy	415.2367	643.3508	13.7	5	8.05	y6
EF-G	LAASIAFK.heavy	415.2367	571.3166	13.7	5	8.05	y5
EF-G	LAASIAFK.heavy	415.2367	187.1225	13.7	5	8.05	b2
IF1	VTVELTPYDLSK.light	682.869	936.5037	22.2	5	12.51	y8
IF1	VTVELTPYDLSK.light	682.869	823.4196	22.2	5	12.51	y7
IF1	VTVELTPYDLSK.light	682.869	722.3719	22.2	5	12.51	y6
IF1	VTVELTPYDLSK.light	682.869	429.2344	22.2	5	12.51	b4
IF1	VTVELTPYDLSK.heavy	688.8512	945.477	22.2	5	12.51	y8
IF1	VTVELTPYDLSK.heavy	688.8512	831.3959	22.2	5	12.51	y7
IF1	VTVELTPYDLSK.heavy	688.8512	729.3512	22.2	5	12.51	y6
IF1	VTVELTPYDLSK.heavy	688.8512	432.2255	22.2	5	12.51	b4
IF2	SVQIEVR.light	415.7402	644.3726	13.9	5	4.82	y5
IF2	SVQIEVR.light	415.7402	516.314	13.9	5	4.82	y4

Table S2 (continued)

Protein	Compound name	Precursor ion *m/z*	Product ion *m/z*	Collision energy (eV)	Cell accelerator voltage (V)	Ret time (min)	Ion name
IF2	SVQIEVR.light	415.7402	403.23	13.9	5	4.82	y3
IF2	SVQIEVR.light	415.7402	187.1077	13.9	5	4.82	b2
IF2	SVQIEVR.heavy	420.7253	652.3489	13.9	5	4.82	y5
IF2	SVQIEVR.heavy	420.7253	522.2962	13.9	5	4.82	y4
IF2	SVQIEVR.heavy	420.7253	408.2151	13.9	5	4.82	y3
IF2	SVQIEVR.heavy	420.7253	189.1018	13.9	5	4.82	b2
IF2	TSLLDYIR.light	490.7742	679.3774	16.2	5	14.43	y5
IF2	TSLLDYIR.light	490.7742	189.087	16.2	5	14.43	b2
IF2	TSLLDYIR.light	490.7742	302.171	16.2	5	14.43	b3
IF2	TSLLDYIR.heavy	496.2579	687.3536	16.2	5	14.43	y5
IF2	TSLLDYIR.heavy	496.2579	191.0811	16.2	5	14.43	b2
IF2	TSLLDYIR.heavy	496.2579	305.1622	16.2	5	14.43	b3
IF3	FLEEGDK.light	419.2031	577.2464	14	5	3.48	y5
IF3	FLEEGDK.light	419.2031	448.2038	14	5	3.48	y4
IF3	FLEEGDK.light	419.2031	319.1612	14	5	3.48	y3
IF3	FLEEGDK.light	419.2031	261.1598	14	5	3.48	b2
IF3	FLEEGDK.heavy	422.1942	581.2345	14	5	3.48	y5
IF3	FLEEGDK.heavy	422.1942	452.1919	14	5	3.48	y4
IF3	FLEEGDK.heavy	422.1942	323.1494	14	5	3.48	y3
IF3	FLEEGDK.heavy	422.1942	263.1538	14	5	3.48	b2
EF-Ts	ITDVEVLK.light	458.7711	803.4509	15.2	5	7.46	y7
EF-Ts	ITDVEVLK.light	458.7711	702.4032	15.2	5	7.46	y6
EF-Ts	ITDVEVLK.light	458.7711	215.139	15.2	5	7.46	b2
EF-Ts	ITDVEVLK.light	458.7711	215.1208	15.2	5	7.46	b4
EF-Ts	ITDVEVLK.heavy	462.7593	810.4302	15.2	5	7.46	y7
EF-Ts	ITDVEVLK.heavy	462.7593	708.3854	15.2	5	7.46	y6
EF-Ts	ITDVEVLK.heavy	462.7593	217.1331	15.2	5	7.46	b2
EF-Ts	ITDVEVLK.heavy	462.7593	217.1149	15.2	5	7.46	b4
EF-Ts	IGVLVAAK.light	385.7604	657.4294	13	5	6.48	y7
EF-Ts	IGVLVAAK.light	385.7604	600.4079	13	5	6.48	y6
EF-Ts	IGVLVAAK.light	385.7604	501.3395	13	5	6.48	y5
EF-Ts	IGVLVAAK.light	385.7604	171.1128	13	5	6.48	b2
EF-Ts	IGVLVAAK.heavy	390.247	665.4057	13	5	6.48	y7
EF-Ts	IGVLVAAK.heavy	390.247	607.3872	13	5	6.48	y6
EF-Ts	IGVLVAAK.heavy	390.247	507.3217	13	5	6.48	y5
EF-Ts	IGVLVAAK.heavy	390.247	173.1069	13	5	6.48	b2
EF-Ts	TVGQLLK.light	379.7422	657.4294	12.8	5	4.93	y6
EF-Ts	TVGQLLK.light	379.7422	558.361	12.8	5	4.93	y5
EF-Ts	TVGQLLK.light	379.7422	201.1234	12.8	5	4.93	b2

Table S2 (continued)

Protein	Compound name	Precursor ion *m/z*	Product ion *m/z*	Collision energy (eV)	Cell accelerator voltage (V)	Ret time (min)	Ion name
EF-Ts	TVGQLLK.heavy	384.2288	665.4057	12.8	5	4.93	y6
EF-Ts	TVGQLLK.heavy	384.2288	565.3402	12.8	5	4.93	y5
EF-Ts	TVGQLLK.heavy	384.2288	203.1174	12.8	5	4.93	b2
RF1	INLTLYR.light	446.7662	665.3981	14.8	5	9.77	y5
RF1	INLTLYR.light	446.7662	552.314	14.8	5	9.77	y4
RF1	INLTLYR.light	446.7662	228.1343	14.8	5	9.77	b2
RF1	INLTLYR.heavy	452.2499	673.3744	14.8	5	9.77	y5
RF1	INLTLYR.heavy	452.2499	559.2933	14.8	5	9.77	y4
RF1	INLTLYR.heavy	452.2499	231.1254	14.8	5	9.77	b2
FMT	IIFAGTPDFAAR.light	639.8457	1052.516	20.8	5	13.1	y10
FMT	IIFAGTPDFAAR.light	639.8457	834.4104	20.8	5	13.1	y8
FMT	IIFAGTPDFAAR.light	639.8457	676.3413	20.8	5	13.1	y6
FMT	IIFAGTPDFAAR.light	639.8457	227.1754	20.8	5	13.1	b2
FMT	IIFAGTPDFAAR.heavy	647.3235	1065.477	20.8	5	13.1	y10
FMT	IIFAGTPDFAAR.heavy	647.3235	845.3778	20.8	5	13.1	y8
FMT	IIFAGTPDFAAR.heavy	647.3235	685.3146	20.8	5	13.1	y6
FMT	IIFAGTPDFAAR.heavy	647.3235	229.1695	20.8	5	13.1	b2

Abbreviation: Ret time, retention time.

Table S3: Transitions of the MS/MS measurements for the proteins expressed from pTFM1 (injection 2)

Protein	Compound name	Precursor ion *m/z*	Product ion *m/z*	Collision energy (eV)	Cell accelerator voltage (V)	Ret time (min)	Ion name
tryptophan tRNA ligase	NNVIGLLEDPK.light	606.3352	884.5088	19.8	5	15.04	y8
tryptophan tRNA ligase	NNVIGLLEDPK.light	606.3352	771.4247	19.8	5	15.04	y7
tryptophan tRNA ligase	NNVIGLLEDPK.light	606.3352	244.1656	19.8	5	15.04	y2
tryptophan tRNA ligase	NNVIGLLEDPK.light	606.3352	328.1615	19.8	5	15.04	b3
tryptophan tRNA ligase	NNVIGLLEDPK.heavy	612.8159	892.485	19.8	5	15.04	y8
tryptophan tRNA ligase	NNVIGLLEDPK.heavy	612.8159	778.4039	19.8	5	15.04	y7
tryptophan tRNA ligase	NNVIGLLEDPK.heavy	612.8159	247.1567	19.8	5	15.04	y2
tryptophan tRNA ligase	NNVIGLLEDPK.heavy	612.8159	333.1467	19.8	5	15.04	b3
EF-Tu	TTLTAAITTVLAK.light	652.3952	887.556	21.2	5	15.88	y9
EF-Tu	TTLTAAITTVLAK.light	652.3952	816.5189	21.2	5	15.88	y8
EF-Tu	TTLTAAITTVLAK.light	652.3952	745.4818	21.2	5	15.88	y7
EF-Tu	TTLTAAITTVLAK.light	652.3952	632.3978	21.2	5	15.88	y6
EF-Tu	TTLTAAITTVLAK.heavy	659.3745	897.5264	21.2	5	15.88	y9
EF-Tu	TTLTAAITTVLAK.heavy	659.3745	825.4922	21.2	5	15.88	y8
EF-Tu	TTLTAAITTVLAK.heavy	659.3745	753.4581	21.2	5	15.88	y7
EF-Tu	TTLTAAITTVLAK.heavy	659.3745	639.377	21.2	5	15.88	y6
IF3	LTGLEGEQLGIVSLR.light	792.9514	1071.616	25.6	5	15.4	y10
IF3	LTGLEGEQLGIVSLR.light	792.9514	757.4931	25.6	5	15.4	y7
IF3	LTGLEGEQLGIVSLR.light	792.9514	474.3035	25.6	5	15.4	y4
IF3	LTGLEGEQLGIVSLR.light	792.9514	375.235	25.6	5	15.4	y3
IF3	LTGLEGEQLGIVSLR.heavy	801.4262	1084.577	25.6	5	15.4	y10
IF3	LTGLEGEQLGIVSLR.heavy	801.4262	767.4634	25.6	5	15.4	y7
IF3	LTGLEGEQLGIVSLR.heavy	801.4262	481.2827	25.6	5	15.4	y4
IF3	LTGLEGEQLGIVSLR.heavy	801.4262	381.2173	25.6	5	15.4	y3
RF1	TYNFPQGR.light	491.7407	604.3202	16.2	5	5.22	y5
RF1	TYNFPQGR.light	491.7407	457.2518	16.2	5	5.22	y4
RF1	TYNFPQGR.light	491.7407	265.1183	16.2	5	5.22	b2
RF1	TYNFPQGR.light	491.7407	379.1612	16.2	5	5.22	b3
RF1	TYNFPQGR.heavy	498.2214	613.2935	16.2	5	5.22	y5
RF1	TYNFPQGR.heavy	498.2214	465.228	16.2	5	5.22	y4
RF1	TYNFPQGR.heavy	498.2214	267.1124	16.2	5	5.22	b2
RF1	TYNFPQGR.heavy	498.2214	383.1494	16.2	5	5.22	b3
RF2	SYVLDDSR.light	477.73	704.3573	15.8	5	4.97	y6
RF2	SYVLDDSR.light	477.73	605.2889	15.8	5	4.97	y5

Table S3 (continued)

Protein	Compound name	Precursor ion *m/z*	Product ion *m/z*	Collision energy (eV)	Cell accelerator voltage (V)	Ret time (min)	Ion name
RF2	SYVLDDSR.light	477.73	492.2049	15.8	5	4.97	y4
RF2	SYVLDDSR.light	477.73	251.1026	15.8	5	4.97	b2
RF2	SYVLDDSR.heavy	483.2137	713.3307	15.8	5	4.97	y6
RF2	SYVLDDSR.heavy	483.2137	613.2652	15.8	5	4.97	y5
RF2	SYVLDDSR.heavy	483.2137	499.1841	15.8	5	4.97	y4
RF2	SYVLDDSR.heavy	483.2137	253.0967	15.8	5	4.97	b2
RF3	VLLFGQAIQTAGTVK.light	773.4536	817.4778	25	5	14.73	y8
RF3	VLLFGQAIQTAGTVK.light	773.4536	475.2875	25	5	14.73	y5
RF3	VLLFGQAIQTAGTVK.light	773.4536	213.1598	25	5	14.73	b2
RF3	VLLFGQAIQTAGTVK.light	773.4536	326.2438	25	5	14.73	b3
RF3	VLLFGQAIQTAGTVK.heavy	782.4269	827.4481	25	5	14.73	y8
RF3	VLLFGQAIQTAGTVK.heavy	782.4269	481.2697	25	5	14.73	y5
RF3	VLLFGQAIQTAGTVK.heavy	782.4269	215.1538	25	5	14.73	b2
RF3	VLLFGQAIQTAGTVK.heavy	782.4269	329.2349	25	5	14.73	b3
RRF	INVFDR.light	382.2085	650.3257	12.8	5	6.46	y5
RRF	INVFDR.light	382.2085	536.2827	12.8	5	6.46	y4
RRF	INVFDR.light	382.2085	437.2143	12.8	5	6.46	y3
RRF	INVFDR.light	382.2085	228.1343	12.8	5	6.46	b2
RRF	INVFDR.heavy	387.1937	659.299	12.8	5	6.46	y5
RRF	INVFDR.heavy	387.1937	543.262	12.8	5	6.46	y4
RRF	INVFDR.heavy	387.1937	443.1965	12.8	5	6.46	y3
RRF	INVFDR.heavy	387.1937	231.1254	12.8	5	6.46	b2
FMT	LAELGPQGLITTLK.light	727.4349	1027.615	23.6	5	15.29	y10
FMT	LAELGPQGLITTLK.light	727.4349	970.5932	23.6	5	15.29	y9
FMT	LAELGPQGLITTLK.light	727.4349	314.171	23.6	5	15.29	b3
FMT	LAELGPQGLITTLK.heavy	734.9126	1039.579	23.6	5	15.29	y10
FMT	LAELGPQGLITTLK.heavy	734.9126	981.5605	23.6	5	15.29	y9
FMT	LAELGPQGLITTLK.heavy	734.9126	316.1651	23.6	5	15.29	b3
tyrosine tRNA ligase	AQYVLAEQVTR.light	639.3461	632.3362	20.8	5	8.34	y5
tyrosine tRNA ligase	AQYVLAEQVTR.light	639.3461	503.2936	20.8	5	8.34	y4
tyrosine tRNA ligase	AQYVLAEQVTR.light	639.3461	375.235	20.8	5	8.34	y3
tyrosine tRNA ligase	AQYVLAEQVTR.light	639.3461	276.1666	20.8	5	8.34	y2
tyrosine tRNA ligase	AQYVLAEQVTR.heavy	646.8238	640.3125	20.8	5	8.34	y5
tyrosine tRNA ligase	AQYVLAEQVTR.heavy	646.8238	511.2699	20.8	5	8.34	y4
tyrosine tRNA ligase	AQYVLAEQVTR.heavy	646.8238	381.2173	20.8	5	8.34	y3
tyrosine tRNA ligase	AQYVLAEQVTR.heavy	646.8238	281.1518	20.8	5	8.34	y2
threonine tRNA ligase	MHELAEK.light	429.2129	726.3781	14.3	5	1.12	y6
threonine tRNA ligase	MHELAEK.light	429.2129	363.6927	14.3	5	1.12	y6

Table S3 (continued)

Protein	Compound name	Precursor ion *m/z*	Product ion *m/z*	Collision energy (eV)	Cell accelerator voltage (V)	Ret time (min)	Ion name
threonine tRNA ligase	MHELAEK.light	429.2129	269.1067	14.3	5	1.12	b2
threonine tRNA ligase	MHELAEK.heavy	433.2011	733.3573	14.3	5	1.12	y6
threonine tRNA ligase	MHELAEK.heavy	433.2011	367.1823	14.3	5	1.12	y6
threonine tRNA ligase	MHELAEK.heavy	433.2011	273.0948	14.3	5	1.12	b2
threonine tRNA ligase	ALNAYLQR.light	474.7667	764.405	15.7	5	6.76	y6
threonine tRNA ligase	ALNAYLQR.light	474.7667	650.362	15.7	5	6.76	y5
threonine tRNA ligase	ALNAYLQR.light	474.7667	303.1775	15.7	5	6.76	y2
threonine tRNA ligase	ALNAYLQR.light	474.7667	370.2085	15.7	5	6.76	b4
threonine tRNA ligase	ALNAYLQR.heavy	481.2474	775.3723	15.7	5	6.76	y6
threonine tRNA ligase	ALNAYLQR.heavy	481.2474	659.3354	15.7	5	6.76	y5
threonine tRNA ligase	ALNAYLQR.heavy	481.2474	309.1597	15.7	5	6.76	y2
threonine tRNA ligase	ALNAYLQR.heavy	481.2474	375.1937	15.7	5	6.76	b4
isoleucine tRNA ligase	WTDDDLYGIIR.light	683.8355	1079.537	22.2	5	15.27	y9
isoleucine tRNA ligase	WTDDDLYGIIR.light	683.8355	458.3085	22.2	5	15.27	y4
isoleucine tRNA ligase	WTDDDLYGIIR.light	683.8355	288.203	22.2	5	15.27	y2
isoleucine tRNA ligase	WTDDDLYGIIR.light	683.8355	288.1343	22.2	5	15.27	b2
isoleucine tRNA ligase	WTDDDLYGIIR.heavy	691.3133	1091.501	22.2	5	15.27	y9
isoleucine tRNA ligase	WTDDDLYGIIR.heavy	691.3133	465.2878	22.2	5	15.27	y4
isoleucine tRNA ligase	WTDDDLYGIIR.heavy	691.3133	293.1882	22.2	5	15.27	y2
isoleucine tRNA ligase	WTDDDLYGIIR.heavy	691.3133	291.1254	22.2	5	15.27	b2
glycine tRNA ligase_ beta subunit	SDEVLSDR.light	460.7196	589.3304	15.3	5	3.05	y5
glycine tRNA ligase_ beta subunit	SDEVLSDR.light	460.7196	490.262	15.3	5	3.05	y4
glycine tRNA ligase_ beta subunit	SDEVLSDR.light	460.7196	377.1779	15.3	5	3.05	y3
glycine tRNA ligase_ beta subunit	SDEVLSDR.heavy	465.7048	597.3067	15.3	5	3.05	y5
glycine tRNA ligase_ beta subunit	SDEVLSDR.heavy	465.7048	497.2412	15.3	5	3.05	y4
glycine tRNA ligase_ beta subunit	SDEVLSDR.heavy	465.7048	383.1601	15.3	5	3.05	y3

Table S3 (continued)

Protein	Compound name	Precursor ion *m/z*	Product ion *m/z*	Collision energy (eV)	Cell accelerator voltage (V)	Ret time (min)	Ion name
glutamate tRNA ligase	ALDFIAER.light	467.7533	750.3781	15.5	5	9.61	y6
glutamate tRNA ligase	ALDFIAER.light	467.7533	488.2827	15.5	5	9.61	y4
glutamate tRNA ligase	ALDFIAER.light	467.7533	375.1987	15.5	5	9.61	y3
glutamate tRNA ligase	ALDFIAER.light	467.7533	185.1285	15.5	5	9.61	b2
glutamate tRNA ligase	ALDFIAER.heavy	472.7384	758.3544	15.5	5	9.61	y6
glutamate tRNA ligase	ALDFIAER.heavy	472.7384	494.2649	15.5	5	9.61	y4
glutamate tRNA ligase	ALDFIAER.heavy	472.7384	380.1838	15.5	5	9.61	y3
glutamate tRNA ligase	ALDFIAER.heavy	472.7384	187.1225	15.5	5	9.61	b2
aspartate tRNA ligase	FYALPQSPQLFK.light	719.8901	944.52	23.3	5	15.1	y8
aspartate tRNA ligase	FYALPQSPQLFK.light	719.8901	632.3766	23.3	5	15.1	y5
aspartate tRNA ligase	FYALPQSPQLFK.light	719.8901	382.1761	23.3	5	15.1	b3
aspartate tRNA ligase	FYALPQSPQLFK.light	719.8901	495.2602	23.3	5	15.1	b4
aspartate tRNA ligase	FYALPQSPQLFK.heavy	727.3679	955.4874	23.3	5	15.1	y8
aspartate tRNA ligase	FYALPQSPQLFK.heavy	727.3679	639.3559	23.3	5	15.1	y5
aspartate tRNA ligase	FYALPQSPQLFK.heavy	727.3679	385.1672	23.3	5	15.1	b3
aspartate tRNA ligase	FYALPQSPQLFK.heavy	727.3679	499.2483	23.3	5	15.1	b4
alanine tRNA ligase	VDSASEFK.light	441.7138	783.3519	14.7	5	2.84	y7
alanine tRNA ligase	VDSASEFK.light	441.7138	668.325	14.7	5	2.84	y6
alanine tRNA ligase	VDSASEFK.light	441.7138	294.1812	14.7	5	2.84	y2
alanine tRNA ligase	VDSASEFK.heavy	445.7019	790.3312	14.7	5	2.84	y7
alanine tRNA ligase	VDSASEFK.heavy	445.7019	674.3072	14.7	5	2.84	y6
alanine tRNA ligase	VDSASEFK.heavy	445.7019	297.1723	14.7	5	2.84	y2
histidine tRNA ligase	AIGEVTDVVEK.light	580.3139	975.4993	19	5	8.31	y9
histidine tRNA ligase	AIGEVTDVVEK.light	580.3139	789.4353	19	5	8.31	y7
histidine tRNA ligase	AIGEVTDVVEK.light	580.3139	690.3668	19	5	8.31	y6
histidine tRNA ligase	AIGEVTDVVEK.heavy	585.2991	983.4756	19	5	8.31	y9
histidine tRNA ligase	AIGEVTDVVEK.heavy	585.2991	796.4145	19	5	8.31	y7
histidine tRNA ligase	AIGEVTDVVEK.heavy	585.2991	696.3491	19	5	8.31	y6
RRF	VPLPPLTEER.light	575.827	841.4414	18.9	5	10.09	y7
RRF	VPLPPLTEER.light	575.827	421.2243	18.9	5	10.09	y7
RRF	VPLPPLTEER.light	575.827	197.1285	18.9	5	10.09	b2

Table S3 (continued)

Protein	Compound name	Precursor ion *m/z*	Product ion *m/z*	Collision energy (eV)	Cell accelerator voltage (V)	Ret time (min)	Ion name
RRF	VPLPPLTEER.light	575.827	310.2125	18.9	5	10.09	b3
RRF	VPLPPLTEER.heavy	581.3107	849.4177	18.9	5	10.09	y7
RRF	VPLPPLTEER.heavy	581.3107	425.2125	18.9	5	10.09	y7
RRF	VPLPPLTEER.heavy	581.3107	199.1225	18.9	5	10.09	b2
RRF	VPLPPLTEER.heavy	581.3107	313.2036	18.9	5	10.09	b3
isoleucine tRNA ligase	FLLANLNGFDPAK.light	710.3852	748.3624	23	5	16.53	y7
isoleucine tRNA ligase	FLLANLNGFDPAK.light	710.3852	315.2027	23	5	16.53	y3
isoleucine tRNA ligase	FLLANLNGFDPAK.light	710.3852	261.1598	23	5	16.53	b2
isoleucine tRNA ligase	FLLANLNGFDPAK.light	710.3852	374.2438	23	5	16.53	b3
isoleucine tRNA ligase	FLLANLNGFDPAK.heavy	718.3615	757.3357	23	5	16.53	y7
isoleucine tRNA ligase	FLLANLNGFDPAK.heavy	718.3615	319.1908	23	5	16.53	y3
isoleucine tRNA ligase	FLLANLNGFDPAK.heavy	718.3615	263.1538	23	5	16.53	b2
isoleucine tRNA ligase	FLLANLNGFDPAK.heavy	718.3615	377.2349	23	5	16.53	b3
glycine tRNA ligase_ alpha subunit	AVAEAYYASR.light	550.7722	930.4316	18.1	5	4.96	y8
glycine tRNA ligase_ alpha subunit	AVAEAYYASR.light	550.7722	659.3148	18.1	5	4.96	y5
glycine tRNA ligase_ alpha subunit	AVAEAYYASR.light	550.7722	171.1128	18.1	5	4.96	b2
glycine tRNA ligase_ alpha subunit	AVAEAYYASR.heavy	556.7544	940.4019	18.1	5	4.96	y8
glycine tRNA ligase_ alpha subunit	AVAEAYYASR.heavy	556.7544	667.291	18.1	5	4.96	y5
glycine tRNA ligase_ alpha subunit	AVAEAYYASR.heavy	556.7544	173.1069	18.1	5	4.96	b2
glutamine tRNA ligase	GYTAASIR.light	419.7245	618.357	14	5	3.7	y6
glutamine tRNA ligase	GYTAASIR.light	419.7245	517.3093	14	5	3.7	y5
glutamine tRNA ligase	GYTAASIR.light	419.7245	446.2722	14	5	3.7	y4
glutamine tRNA ligase	GYTAASIR.heavy	425.2082	627.3303	14	5	3.7	y6
glutamine tRNA ligase	GYTAASIR.heavy	425.2082	525.2856	14	5	3.7	y5
glutamine tRNA ligase	GYTAASIR.heavy	425.2082	453.2514	14	5	3.7	y4
tyrosine tRNA ligase	GLVAQVTDEEALAER.light	800.9125	1132.548	25.8	5	12.06	y10
tyrosine tRNA ligase	GLVAQVTDEEALAER.light	800.9125	1033.48	25.8	5	12.06	y9
tyrosine tRNA ligase	GLVAQVTDEEALAER.light	800.9125	488.2827	25.8	5	12.06	y4
tyrosine tRNA ligase	GLVAQVTDEEALAER.light	800.9125	375.1987	25.8	5	12.06	y3

Table S3 (continued)

Protein	Compound name	Precursor ion *m/z*	Product ion *m/z*	Collision energy (eV)	Cell accelerator voltage (V)	Ret time (min)	Ion name
tyrosine tRNA ligase	GLVAQVTDEEALAER.heavy	808.8888	1142.518	25.8	5	12.06	y10
tyrosine tRNA ligase	GLVAQVTDEEALAER.heavy	808.8888	1042.453	25.8	5	12.06	y9
tyrosine tRNA ligase	GLVAQVTDEEALAER.heavy	808.8888	494.2649	25.8	5	12.06	y4
tyrosine tRNA ligase	GLVAQVTDEEALAER.heavy	808.8888	380.1838	25.8	5	12.06	y3
lysine tRNA ligase	ASFVTLQDVGGR.light	625.3304	845.4476	20.4	5	10.71	y8
lysine tRNA ligase	ASFVTLQDVGGR.light	625.3304	744.3999	20.4	5	10.71	y7
lysine tRNA ligase	ASFVTLQDVGGR.light	625.3304	503.2572	20.4	5	10.71	y5
lysine tRNA ligase	ASFVTLQDVGGR.heavy	633.3067	857.412	20.4	5	10.71	y8
lysine tRNA ligase	ASFVTLQDVGGR.heavy	633.3067	755.3673	20.4	5	10.71	y7
lysine tRNA ligase	ASFVTLQDVGGR.heavy	633.3067	511.2335	20.4	5	10.71	y5
aspartate tRNA ligase	DLGLTDESK.light	489.2429	749.3676	16.2	5	5.96	y7
aspartate tRNA ligase	DLGLTDESK.light	489.2429	692.3461	16.2	5	5.96	y6
aspartate tRNA ligase	DLGLTDESK.light	489.2429	579.262	16.2	5	5.96	y5
aspartate tRNA ligase	DLGLTDESK.heavy	493.7296	756.3468	16.2	5	5.96	y7
aspartate tRNA ligase	DLGLTDESK.heavy	493.7296	698.3283	16.2	5	5.96	y6
aspartate tRNA ligase	DLGLTDESK.heavy	493.7296	584.2472	16.2	5	5.96	y5
aspartate tRNA ligase	DYLVPSR.light	425.2269	571.3562	14.2	5	6.52	y5
aspartate tRNA ligase	DYLVPSR.light	425.2269	458.2722	14.2	5	6.52	y4
aspartate tRNA ligase	DYLVPSR.light	425.2269	359.2037	14.2	5	6.52	y3
aspartate tRNA ligase	DYLVPSR.light	425.2269	279.0975	14.2	5	6.52	b2
aspartate tRNA ligase	DYLVPSR.heavy	430.2121	579.3325	14.2	5	6.52	y5
aspartate tRNA ligase	DYLVPSR.heavy	430.2121	465.2514	14.2	5	6.52	y4
aspartate tRNA ligase	DYLVPSR.heavy	430.2121	365.186	14.2	5	6.52	y3
aspartate tRNA ligase	DYLVPSR.heavy	430.2121	281.0916	14.2	5	6.52	b2
EF-Tu	GITINTSHVEYDTPTR.light	601.9672	752.3573	16.9	5	8.67	y6
EF-Tu	GITINTSHVEYDTPTR.light	601.9672	474.2671	16.9	5	8.67	y4
EF-Tu	GITINTSHVEYDTPTR.light	601.9672	710.3286	16.9	5	8.67	y12
EF-Tu	GITINTSHVEYDTPTR.light	601.9672	187.1133	16.9	5	8.67	y3
EF-Tu	GITINTSHVEYDTPTR.heavy	608.9465	761.3307	16.9	5	8.67	y6
EF-Tu	GITINTSHVEYDTPTR.heavy	608.9465	481.2463	16.9	5	8.67	y4
EF-Tu	GITINTSHVEYDTPTR.heavy	608.9465	718.8034	16.9	5	8.67	y12
EF-Tu	GITINTSHVEYDTPTR.heavy	608.9465	190.1044	16.9	5	8.67	y3

Table S3 (continued)

Protein	Compound name	Precursor ion *m/z*	Product ion *m/z*	Collision energy (eV)	Cell accelerator voltage (V)	Ret time (min)	Ion name
leucine tRNA ligase	ITVPVDATEEQVR.light	728.8857	1143.564	23.6	5	9.76	y10
leucine tRNA ligase	ITVPVDATEEQVR.light	728.8857	215.139	23.6	5	9.76	b2
leucine tRNA ligase	ITVPVDATEEQVR.light	728.8857	314.2074	23.6	5	9.76	b3
leucine tRNA ligase	ITVPVDATEEQVR.heavy	736.3635	1155.528	23.6	5	9.76	y10
leucine tRNA ligase	ITVPVDATEEQVR.heavy	736.3635	217.1331	23.6	5	9.76	b2
leucine tRNA ligase	ITVPVDATEEQVR.heavy	736.3635	317.1985	23.6	5	9.76	b3
leucine tRNA ligase	YLDGVTVR.light	461.7533	646.3519	15.3	5	7.09	y6
leucine tRNA ligase	YLDGVTVR.light	461.7533	531.3249	15.3	5	7.09	y5
leucine tRNA ligase	YLDGVTVR.light	461.7533	277.1547	15.3	5	7.09	b2
leucine tRNA ligase	YLDGVTVR.heavy	467.237	655.3252	15.3	5	7.09	y6
leucine tRNA ligase	YLDGVTVR.heavy	467.237	539.3012	15.3	5	7.09	y5
leucine tRNA ligase	YLDGVTVR.heavy	467.237	279.1487	15.3	5	7.09	b2
cysteine tRNA ligase	IFNTLTR.light	432.7505	751.4097	14.4	5	6.56	y6
cysteine tRNA ligase	IFNTLTR.light	432.7505	604.3413	14.4	5	6.56	y5
cysteine tRNA ligase	IFNTLTR.light	432.7505	490.2984	14.4	5	6.56	y4
cysteine tRNA ligase	IFNTLTR.light	432.7505	261.1598	14.4	5	6.56	b2
cysteine tRNA ligase	IFNTLTR.heavy	438.2342	761.3801	14.4	5	6.56	y6
cysteine tRNA ligase	IFNTLTR.heavy	438.2342	613.3146	14.4	5	6.56	y5
cysteine tRNA ligase	IFNTLTR.heavy	438.2342	497.2776	14.4	5	6.56	y4
cysteine tRNA ligase	IFNTLTR.heavy	438.2342	263.1538	14.4	5	6.56	b2
lysine tRNA ligase	VFEINR.light	389.2163	678.357	13.1	5	5.47	y5
lysine tRNA ligase	VFEINR.light	389.2163	531.2885	13.1	5	5.47	y4
lysine tRNA ligase	VFEINR.light	389.2163	402.2459	13.1	5	5.47	y3
lysine tRNA ligase	VFEINR.light	389.2163	247.1441	13.1	5	5.47	b2
lysine tRNA ligase	VFEINR.heavy	393.703	686.3332	13.1	5	5.47	y5
lysine tRNA ligase	VFEINR.heavy	393.703	538.2678	13.1	5	5.47	y4
lysine tRNA ligase	VFEINR.heavy	393.703	409.2252	13.1	5	5.47	y3
lysine tRNA ligase	VFEINR.heavy	393.703	249.1382	13.1	5	5.47	b2
valine tRNA ligase	GFLQTLAR.light	453.2638	588.3464	15.1	5	10.09	y5
valine tRNA ligase	GFLQTLAR.light	453.2638	460.2878	15.1	5	10.09	y4
valine tRNA ligase	GFLQTLAR.light	453.2638	246.1561	15.1	5	10.09	y2
valine tRNA ligase	GFLQTLAR.light	453.2638	205.0972	15.1	5	10.09	b2
valine tRNA ligase	GFLQTLAR.heavy	459.246	597.3197	15.1	5	10.09	y5
valine tRNA ligase	GFLQTLAR.heavy	459.246	467.2671	15.1	5	10.09	y4
valine tRNA ligase	GFLQTLAR.heavy	459.246	251.1412	15.1	5	10.09	y2
valine tRNA ligase	GFLQTLAR.heavy	459.246	207.0912	15.1	5	10.09	b2
RF2	IQDLTER.light	437.7351	761.3788	14.6	5	3.5	y6
RF2	IQDLTER.light	437.7351	633.3202	14.6	5	3.5	y5
RF2	IQDLTER.light	437.7351	242.1499	14.6	5	3.5	b2

Table S3 (continued)

Protein	Compound name	Precursor ion *m/z*	Product ion *m/z*	Collision energy (eV)	Cell accelerator voltage (V)	Ret time (min)	Ion name
RF2	IQDLTER.heavy	442.7202	770.3521	14.6	5	3.5	y6
RF2	IQDLTER.heavy	442.7202	640.2995	14.6	5	3.5	y5
RF2	IQDLTER.heavy	442.7202	245.141	14.6	5	3.5	b2

Abbreviation: Ret time, retention time.

Table S4: Transitions of the MS/MS measurements for Cysteine-tRNA-ligase, Initiation factor 2, Release factor 2, and Elongation factor G

Protein	Compound name	Precursor ion *m/z*	Product ion *m/z*	Collision energy (eV)	Cell accelerator voltage (V)	Ret time (min)	Ion name
CysRS	IFNTLTR.light	432.8	604.3413	14.4	5	6.94	y5
CysRS	IFNTLTR.light	432.8	490.2984	14.4	5	6.94	y4
CysRS	IFNTLTR.light	432.8	261.1598	14.4	5	6.94	b2
CysRS	IFNTLTR.heavy	438.2	613.3146	14.4	5	6.94	y5
CysRS	IFNTLTR.heavy	438.2	497.2776	14.4	5	6.94	y4
CysRS	IFNTLTR.heavy	438.2	263.1538	14.4	5	6.94	b2
CysRS	TFVAFDVVAR.light	562.8	559.3198	18.4	5	13.9	y5
CysRS	TFVAFDVVAR.light	562.8	444.2929	18.4	5	13.9	y4
CysRS	TFVAFDVVAR.light	562.8	249.1234	18.4	5	13.9	b2
CysRS	TFVAFDVVAR.light	562.8	348.1918	18.4	5	13.9	b3
CysRS	TFVAFDVVAR.heavy	569.3	567.2961	18.4	5	13.9	y5
CysRS	TFVAFDVVAR.heavy	569.3	451.2721	18.4	5	13.9	y4
CysRS	TFVAFDVVAR.heavy	569.3	251.1174	18.4	5	13.9	b2
CysRS	TFVAFDVVAR.heavy	569.3	351.1829	18.4	5	13.9	b3
CysRS	SLGNFFTVR.light	520.8	669.3719	17.1	5	13.17	y5
CysRS	SLGNFFTVR.light	520.8	522.3035	17.1	5	13.17	y4
CysRS	SLGNFFTVR.light	520.8	375.235	17.1	5	13.17	y3
CysRS	SLGNFFTVR.light	520.8	201.1234	17.1	5	13.17	b2
CysRS	SLGNFFTVR.heavy	527.3	677.3482	17.1	5	13.17	y5
CysRS	SLGNFFTVR.heavy	527.3	529.2827	17.1	5	13.17	y4
CysRS	SLGNFFTVR.heavy	527.3	381.2173	17.1	5	13.17	y3
CysRS	SLGNFFTVR.heavy	527.3	203.1174	17.1	5	13.17	b2
CysRS	SQLNYSEENLK.light	662.8	216.0979	21.5	5	6.89	b2
CysRS	SQLNYSEENLK.light	662.8	329.1819	21.5	5	6.89	b3
CysRS	SQLNYSEENLK.light	662.8	606.2882	21.5	5	6.89	b5
CysRS	SQLNYSEENLK.heavy	669.3	219.089	21.5	5	6.89	b2
CysRS	SQLNYSEENLK.heavy	669.3	333.1701	21.5	5	6.89	b3
CysRS	SQLNYSEENLK.heavy	669.3	613.2674	21.5	5	6.89	b5
CysRS	DWAAADAAR.light	473.7	503.2572	15.7	5	5.9	y5
CysRS	DWAAADAAR.light	473.7	302.1135	15.7	5	5.9	b2
CysRS	DWAAADAAR.light	473.7	373.1506	15.7	5	5.9	b3
CysRS	DWAAADAAR.heavy	480.2	511.2335	15.7	5	5.9	y5
CysRS	DWAAADAAR.heavy	480.2	305.1046	15.7	5	5.9	b2
CysRS	DWAAADAAR.heavy	480.2	377.1388	15.7	5	5.9	b3
IF2	LVQQFADAGIR.light	609.3	877.4526	19.9	5	9.61	y8
IF2	LVQQFADAGIR.light	609.3	749.3941	19.9	5	9.61	y7
IF2	LVQQFADAGIR.light	609.3	602.3257	19.9	5	9.61	y6

Table S4 (continued)

Protein	Compound name	Precursor ion *m/z*	Product ion *m/z*	Collision energy (eV)	Cell accelerator voltage (V)	Ret time (min)	Ion name
IF2	LVQQFADAGIR.light	609.3	416.2616	19.9	5	9.61	y4
IF2	LVQQFADAGIR.heavy	617.3	889.4171	19.9	5	9.61	y8
IF2	LVQQFADAGIR.heavy	617.3	759.3644	19.9	5	9.61	y7
IF2	LVQQFADAGIR.heavy	617.3	611.299	19.9	5	9.61	y6
IF2	LVQQFADAGIR.heavy	617.3	423.2408	19.9	5	9.61	y4
IF2	SVQIEVR.light	415.7	644.3726	13.9	5	5.09	y5
IF2	SVQIEVR.light	415.7	516.314	13.9	5	5.09	y4
IF2	SVQIEVR.light	415.7	403.23	13.9	5	5.09	y3
IF2	SVQIEVR.light	415.7	274.1874	13.9	5	5.09	y2
IF2	SVQIEVR.heavy	420.7	652.3489	13.9	5	5.09	y5
IF2	SVQIEVR.heavy	420.7	522.2962	13.9	5	5.09	y4
IF2	SVQIEVR.heavy	420.7	408.2151	13.9	5	5.09	y3
IF2	SVQIEVR.heavy	420.7	279.1725	13.9	5	5.09	y2
IF2	LAAEEQAQR.light	508.3	831.3955	16.8	5	1.54	y7
IF2	LAAEEQAQR.light	508.3	631.3158	16.8	5	1.54	y5
IF2	LAAEEQAQR.light	508.3	502.2732	16.8	5	1.54	y4
IF2	LAAEEQAQR.light	508.3	374.2146	16.8	5	1.54	y3
IF2	LAAEEQAQR.heavy	514.2	841.3659	16.8	5	1.54	y7
IF2	LAAEEQAQR.heavy	514.2	640.2891	16.8	5	1.54	y5
IF2	LAAEEQAQR.heavy	514.2	511.2465	16.8	5	1.54	y4
IF2	LAAEEQAQR.heavy	514.2	381.1939	16.8	5	1.54	y3
IF2	TSLLDYIR.light	490.8	792.4614	16.2	5	15	y6
IF2	TSLLDYIR.light	490.8	679.3774	16.2	5	15	y5
IF2	TSLLDYIR.light	490.8	566.2933	16.2	5	15	y4
IF2	TSLLDYIR.heavy	496.3	801.4347	16.2	5	15	y6
IF2	TSLLDYIR.heavy	496.3	687.3536	16.2	5	15	y5
IF2	TSLLDYIR.heavy	496.3	573.2725	16.2	5	15	y4
IF2	AAQVPVVVAVNK.light	597.9	924.5877	19.5	5	9.11	y9
IF2	AAQVPVVVAVNK.light	597.9	825.5193	19.5	5	9.11	y8
IF2	AAQVPVVVAVNK.light	597.9	431.2613	19.5	5	9.11	y4
IF2	AAQVPVVVAVNK.heavy	605.3	935.5551	19.5	5	9.11	y9
IF2	AAQVPVVVAVNK.heavy	605.3	835.4896	19.5	5	9.11	y8
IF2	AAQVPVVVAVNK.heavy	605.3	437.2435	19.5	5	9.11	y4
IF2	GPVATVLVR.light	456.3	757.4931	15.1	5	7.78	y7
IF2	GPVATVLVR.light	456.3	658.4246	15.1	5	7.78	y6
IF2	GPVATVLVR.light	456.3	587.3875	15.1	5	7.78	y5
IF2	GPVATVLVR.light	456.3	486.3398	15.1	5	7.78	y4
IF2	GPVATVLVR.heavy	462.3	767.4634	15.1	5	7.78	y7
IF2	GPVATVLVR.heavy	462.3	667.398	15.1	5	7.78	y6

Table S4 (continued)

Protein	Compound name	Precursor ion *m/z*	Product ion *m/z*	Collision energy (eV)	Cell accelerator voltage (V)	Ret time (min)	Ion name
IF2	GPVATVLVR.heavy	462.3	595.3638	15.1	5	7.78	y5
IF2	GPVATVLVR.heavy	462.3	493.3191	15.1	5	7.78	y4
IF2	QQIIGLAEVR.light	563.8	870.5407	18.5	5	10.78	y8
IF2	QQIIGLAEVR.light	563.8	757.4567	18.5	5	10.78	y7
IF2	QQIIGLAEVR.light	563.8	644.3726	18.5	5	10.78	y6
IF2	QQIIGLAEVR.light	563.8	474.2671	18.5	5	10.78	y4
IF2	QQIIGLAEVR.heavy	570.8	880.5111	18.5	5	10.78	y8
IF2	QQIIGLAEVR.heavy	570.8	766.43	18.5	5	10.78	y7
IF2	QQIIGLAEVR.heavy	570.8	652.3489	18.5	5	10.78	y6
IF2	QQIIGLAEVR.heavy	570.8	480.2493	18.5	5	10.78	y4
RF2	IQDLTER.light	437.7	633.3202	14.6	5	3.72	y5
RF2	IQDLTER.light	437.7	518.2933	14.6	5	3.72	y4
RF2	IQDLTER.light	437.7	405.2092	14.6	5	3.72	y3
RF2	IQDLTER.light	437.7	242.1499	14.6	5	3.72	b2
RF2	IQDLTER.heavy	442.7	640.2995	14.6	5	3.72	y5
RF2	IQDLTER.heavy	442.7	524.2755	14.6	5	3.72	y4
RF2	IQDLTER.heavy	442.7	410.1944	14.6	5	3.72	y3
RF2	IQDLTER.heavy	442.7	245.141	14.6	5	3.72	b2
RF2	GYLDYDAK.light	472.7	611.2671	15.7	5	7.01	y5
RF2	GYLDYDAK.light	472.7	218.1499	15.7	5	7.01	y2
RF2	GYLDYDAK.light	472.7	221.0921	15.7	5	7.01	b2
RF2	GYLDYDAK.heavy	477.2	617.2493	15.7	5	7.01	y5
RF2	GYLDYDAK.heavy	477.2	221.141	15.7	5	7.01	y2
RF2	GYLDYDAK.heavy	477.2	223.0861	15.7	5	7.01	b2
RF2	LAQLEFR.light	438.8	692.3726	14.6	5	8.67	y5
RF2	LAQLEFR.light	438.8	564.314	14.6	5	8.67	y4
RF2	LAQLEFR.light	438.8	451.23	14.6	5	8.67	y3
RF2	LAQLEFR.light	438.8	185.1285	14.6	5	8.67	b2
RF2	LAQLEFR.heavy	443.7	700.3489	14.6	5	8.67	y5
RF2	LAQLEFR.heavy	443.7	570.2962	14.6	5	8.67	y4
RF2	LAQLEFR.heavy	443.7	456.2151	14.6	5	8.67	y3
RF2	LAQLEFR.heavy	443.7	187.1225	14.6	5	8.67	b2
RF2	ISGDYAYGWLR.light	650.8	694.3671	21.2	5	13.66	y5
RF2	ISGDYAYGWLR.light	650.8	531.3038	21.2	5	13.66	y4
RF2	ISGDYAYGWLR.light	650.8	201.1234	21.2	5	13.66	b2
RF2	ISGDYAYGWLR.heavy	658.3	703.3404	21.2	5	13.66	y5
RF2	ISGDYAYGWLR.heavy	658.3	539.2801	21.2	5	13.66	y4
RF2	ISGDYAYGWLR.heavy	658.3	203.1174	21.2	5	13.66	b2
RF2	SYVLDDSR.light	477.7	605.2889	15.8	5	5.26	y5

Table S4 (continued)

Protein	Compound name	Precursor ion *m/z*	Product ion *m/z*	Collision energy (eV)	Cell accelerator voltage (V)	Ret time (min)	Ion name
RF2	SYVLDDSR.light	477.7	492.2049	15.8	5	5.26	y4
RF2	SYVLDDSR.light	477.7	251.1026	15.8	5	5.26	b2
RF2	SYVLDDSR.light	477.7	350.171	15.8	5	5.26	b3
RF2	SYVLDDSR.heavy	483.2	613.2652	15.8	5	5.26	y5
RF2	SYVLDDSR.heavy	483.2	499.1841	15.8	5	5.26	y4
RF2	SYVLDDSR.heavy	483.2	253.0967	15.8	5	5.26	b2
RF2	SYVLDDSR.heavy	483.2	353.1622	15.8	5	5.26	b3
EF-G	YLGGEELTEAEIK.light	726.4	460.2766	23.5	5	11.52	y4
EF-G	YLGGEELTEAEIK.light	726.4	260.1969	23.5	5	11.52	y2
EF-G	YLGGEELTEAEIK.light	726.4	277.1547	23.5	5	11.52	b2
EF-G	YLGGEELTEAEIK.light	726.4	334.1761	23.5	5	11.52	b3
EF-G	YLGGEELTEAEIK.heavy	731.4	464.2647	23.5	5	11.52	y4
EF-G	YLGGEELTEAEIK.heavy	731.4	263.188	23.5	5	11.52	y2
EF-G	YLGGEELTEAEIK.heavy	731.4	279.1487	23.5	5	11.52	b2
EF-G	YLGGEELTEAEIK.heavy	731.4	337.1672	23.5	5	11.52	b3
EF-G	IATDPFVGNLTFFR.light	799.4	1197.642	25.8	5	19.57	y10
EF-G	IATDPFVGNLTFFR.light	799.4	854.4519	25.8	5	19.57	y7
EF-G	IATDPFVGNLTFFR.light	799.4	185.1285	25.8	5	19.57	b2
EF-G	IATDPFVGNLTFFR.light	799.4	401.2031	25.8	5	19.57	b4
EF-G	IATDPFVGNLTFFR.heavy	808.4	1211.6	25.8	5	19.57	y10
EF-G	IATDPFVGNLTFFR.heavy	808.4	865.4193	25.8	5	19.57	y7
EF-G	IATDPFVGNLTFFR.heavy	808.4	187.1225	25.8	5	19.57	b2
EF-G	IATDPFVGNLTFFR.heavy	808.4	405.1912	25.8	5	19.57	b4
EF-G	AGDIAAAIGLK.light	500.3	643.4137	16.5	5	11.72	y7
EF-G	AGDIAAAIGLK.light	500.3	572.3766	16.5	5	11.72	y6
EF-G	AGDIAAAIGLK.light	500.3	129.0659	16.5	5	11.72	b2
EF-G	AGDIAAAIGLK.light	500.3	244.0928	16.5	5	11.72	b3
EF-G	AGDIAAAIGLK.heavy	506.3	651.39	16.5	5	11.72	y7
EF-G	AGDIAAAIGLK.heavy	506.3	579.3559	16.5	5	11.72	y6
EF-G	AGDIAAAIGLK.heavy	506.3	131.0599	16.5	5	11.72	b2
EF-G	AGDIAAAIGLK.heavy	506.3	247.0839	16.5	5	11.72	b3
EF-G	GIQEQLK.light	408.2	645.3566	13.7	5	3.67	y5
EF-G	GIQEQLK.light	408.2	517.298	13.7	5	3.67	y4
EF-G	GIQEQLK.light	408.2	171.1128	13.7	5	3.67	b2
EF-G	GIQEQLK.heavy	412.7	652.3359	13.7	5	3.67	y5
EF-G	GIQEQLK.heavy	412.7	522.2832	13.7	5	3.67	y4
EF-G	GIQEQLK.heavy	412.7	173.1069	13.7	5	3.67	b2
EF-G	LAASIAFK.light	410.7	636.3715	13.7	5	8.44	y6
EF-G	LAASIAFK.light	410.7	565.3344	13.7	5	8.44	y5

Table S4 (continued)

Protein	Compound name	Precursor ion *m/z*	Product ion *m/z*	Collision energy (eV)	Cell accelerator voltage (V)	Ret time (min)	Ion name
EF-G	LAASIAFK.light	410.7	365.2183	13.7	5	8.44	y3
EF-G	LAASIAFK.light	410.7	185.1285	13.7	5	8.44	b2
EF-G	LAASIAFK.heavy	415.2	643.3508	13.7	5	8.44	y6
EF-G	LAASIAFK.heavy	415.2	571.3166	13.7	5	8.44	y5
EF-G	LAASIAFK.heavy	415.2	369.2065	13.7	5	8.44	y3
EF-G	LAASIAFK.heavy	415.2	187.1225	13.7	5	8.44	b2

Abbreviation: Ret time, retention time.

Table S5: Transitions of the MS/MS measurements for FtsZ

Compound name	Precursor ion *m/z*	Product ion *m/z*	Collision energy (eV)	Cell accelerator voltage (V)	Ret time (min)	Ion name
TAVGQTIQIGSGITK.light	737.4172	1017.594	23.9	5	9.97	y10
TAVGQTIQIGSGITK.light	737.4172	916.5462	23.9	5	9.97	y9
TAVGQTIQIGSGITK.light	737.4172	803.4621	23.9	5	9.97	y8
TAVGQTIQIGSGITK.light	737.4172	675.4036	23.9	5	9.97	y7
TAVGQTIQIGSGITK.light	737.4172	562.3195	23.9	5	9.97	y6
TAVGQTIQIGSGITK.light	737.4172	173.0921	23.9	5	9.97	b2
TAVGQTIQIGSGITK.heavy	746.3905	1029.558	23.9	5	9.97	y10
TAVGQTIQIGSGITK.heavy	746.3905	927.5136	23.9	5	9.97	y9
TAVGQTIQIGSGITK.heavy	746.3905	813.4325	23.9	5	9.97	y8
TAVGQTIQIGSGITK.heavy	746.3905	683.3798	23.9	5	9.97	y7
TAVGQTIQIGSGITK.heavy	746.3905	569.2987	23.9	5	9.97	y6
TAVGQTIQIGSGITK.heavy	746.3905	175.0861	23.9	5	9.97	b2
GLGAGANPEVGR.light	549.2885	799.4057	18	5	4.81	y8
GLGAGANPEVGR.light	549.2885	671.3471	18	5	4.81	y6
GLGAGANPEVGR.light	549.2885	557.3042	18	5	4.81	y5
GLGAGANPEVGR.light	549.2885	171.1128	18	5	4.81	b2
GLGAGANPEVGR.light	549.2885	228.1343	18	5	4.81	b3
GLGAGANPEVGR.heavy	557.2648	811.3701	18	5	4.81	y8
GLGAGANPEVGR.heavy	557.2648	681.3175	18	5	4.81	y6
GLGAGANPEVGR.heavy	557.2648	565.2805	18	5	4.81	y5
GLGAGANPEVGR.heavy	557.2648	173.1069	18	5	4.81	b2
GLGAGANPEVGR.heavy	557.2648	231.1254	18	5	4.81	b3
MAFAEQGITELSK.light	712.8581	1004.526	23.1	5	11.9	y9
MAFAEQGITELSK.light	712.8581	875.4833	23.1	5	11.9	y8
MAFAEQGITELSK.light	712.8581	747.4247	23.1	5	11.9	y7
MAFAEQGITELSK.light	712.8581	203.0849	23.1	5	11.9	b2
MAFAEQGITELSK.light	712.8581	350.1533	23.1	5	11.9	b3
MAFAEQGITELSK.heavy	720.3359	1015.493	23.1	5	11.9	y9
MAFAEQGITELSK.heavy	720.3359	885.4536	23.1	5	11.9	y8
MAFAEQGITELSK.heavy	720.3359	755.401	23.1	5	11.9	y7
MAFAEQGITELSK.heavy	720.3359	205.0789	23.1	5	11.9	b2
MAFAEQGITELSK.heavy	720.3359	353.1444	23.1	5	11.9	b3
HVDSLITIPNDK.light	676.3644	1115.594	22	5	8.95	y10
HVDSLITIPNDK.light	676.3644	473.2354	22	5	8.95	y4
HVDSLITIPNDK.light	676.3644	376.1827	22	5	8.95	y3
HVDSLITIPNDK.light	676.3644	237.1346	22	5	8.95	b2
HVDSLITIPNDK.light	676.3644	439.1936	22	5	8.95	b4
HVDSLITIPNDK.heavy	684.3407	1127.559	22	5	8.95	y10

Table S5 (continued)

Compound name	Precursor ion *m/z*	Product ion *m/z*	Collision energy (eV)	Cell accelerator voltage (V)	Ret time (min)	Ion name
HVDSLITIPNDK.heavy	684.3407	479.2176	22	5	8.95	y4
HVDSLITIPNDK.heavy	684.3407	381.1678	22	5	8.95	y3
HVDSLITIPNDK.heavy	684.3407	241.1227	22	5	8.95	b2
HVDSLITIPNDK.heavy	684.3407	445.1758	22	5	8.95	b4
GISLLDAFGAANDVLK.light	802.4381	1120.563	25.9	5	20.51	y11
GISLLDAFGAANDVLK.light	802.4381	934.4993	25.9	5	20.51	y9
GISLLDAFGAANDVLK.light	802.4381	787.4308	25.9	5	20.51	y8
GISLLDAFGAANDVLK.light	802.4381	659.3723	25.9	5	20.51	y6
GISLLDAFGAANDVLK.light	802.4381	171.1128	25.9	5	20.51	b2
GISLLDAFGAANDVLK.heavy	811.4115	1133.525	25.9	5	20.51	y11
GISLLDAFGAANDVLK.heavy	811.4115	945.4666	25.9	5	20.51	y9
GISLLDAFGAANDVLK.heavy	811.4115	797.4012	25.9	5	20.51	y8
GISLLDAFGAANDVLK.heavy	811.4115	667.3485	25.9	5	20.51	y6
GISLLDAFGAANDVLK.heavy	811.4115	173.1069	25.9	5	20.51	b2
LDEFETVGNTIR.light	697.3515	889.4738	22.6	5	11.16	y8
LDEFETVGNTIR.light	697.3515	760.4312	22.6	5	11.16	y7
LDEFETVGNTIR.light	697.3515	560.3151	22.6	5	11.16	y5
LDEFETVGNTIR.light	697.3515	229.1183	22.6	5	11.16	b2
LDEFETVGNTIR.light	697.3515	358.1609	22.6	5	11.16	b3
LDEFETVGNTIR.heavy	705.3278	901.4382	22.6	5	11.16	y8
LDEFETVGNTIR.heavy	705.3278	771.3986	22.6	5	11.16	y7
LDEFETVGNTIR.heavy	705.3278	569.2884	22.6	5	11.16	y5
LDEFETVGNTIR.heavy	705.3278	231.1124	22.6	5	11.16	b2
LDEFETVGNTIR.heavy	705.3278	361.152	22.6	5	11.16	b3
VVNDNAPQTAK.light	578.8015	958.4588	18.9	5	1.29	y9
VVNDNAPQTAK.light	578.8015	729.389	18.9	5	1.29	y7
VVNDNAPQTAK.light	578.8015	544.3089	18.9	5	1.29	y5
VVNDNAPQTAK.light	578.8015	199.1441	18.9	5	1.29	b2
VVNDNAPQTAK.light	578.8015	313.187	18.9	5	1.29	b3
VVNDNAPQTAK.heavy	586.2792	971.4203	18.9	5	1.29	y9
VVNDNAPQTAK.heavy	586.2792	739.3593	18.9	5	1.29	y7
VVNDNAPQTAK.heavy	586.2792	551.2882	18.9	5	1.29	y5
VVNDNAPQTAK.heavy	586.2792	201.1382	18.9	5	1.29	b2
VVNDNAPQTAK.heavy	586.2792	317.1752	18.9	5	1.29	b3

Abbreviation: Ret time, retention time.

SUPPLEMENTARY FIGURES

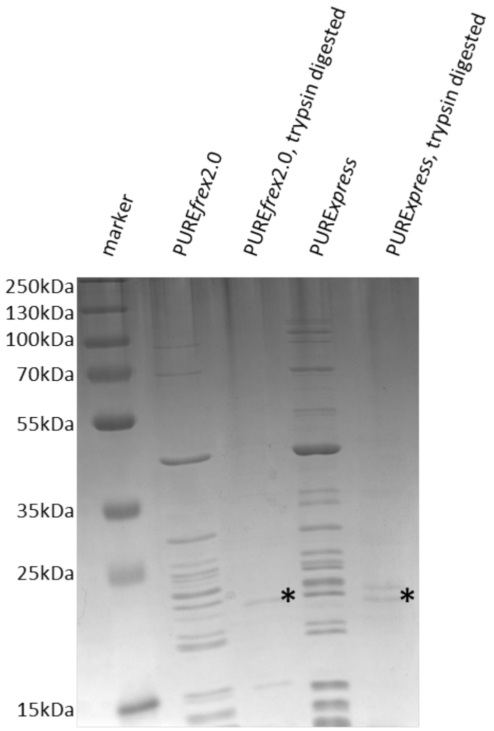

Fig. S1: SDS-PAGE analysis of trypsin digests. The asterisk denotes trypsin (23.3 kDa).

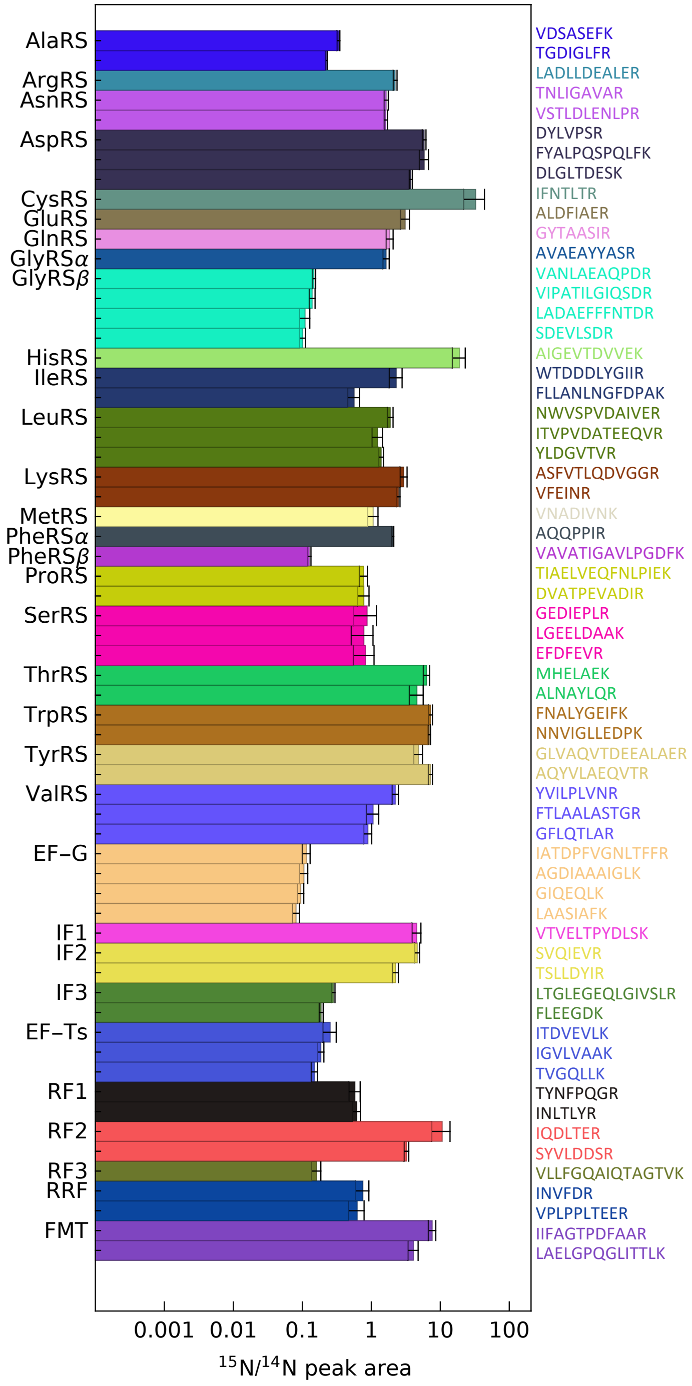

Fig. S2: Relative expression levels of proteins expressed from pTFM1 with PURE*frex*2.0 after 5 h incubation at 37 °C. Ratios of ^{15}N-labeled peptides (newly synthesized proteins) to ^{14}N peptides (original proteins) are displayed for all measured peptides; for proteins with multiple measured peptides, the peptides are ordered from N- to C-terminus (top to bottom). The amino acid sequence of the specific peptides is annotated. Error bars denote ± 1 standard deviation from triplicate experiments. A different batch of PURE*frex*2.0 was used compared to measurements reported in main text Fig. 2B.

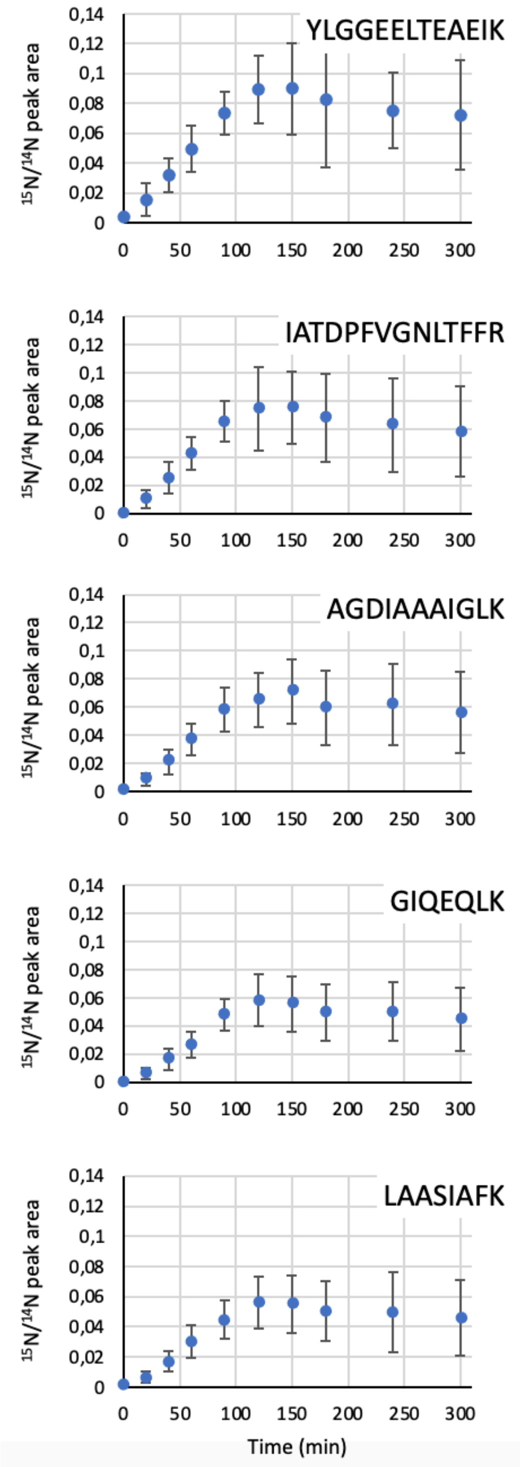

Fig. S3: Time course of $^{15}N/^{14}N$ ratio of selected peptides for translation factor EF-G. Expression of pTFM1 was performed in PURE*frex*2.0. Data from main text Fig. 3B are replotted here to show individual kinetics for the indicated peptides. From top to bottom: most N-terminal to most C-terminal peptides.

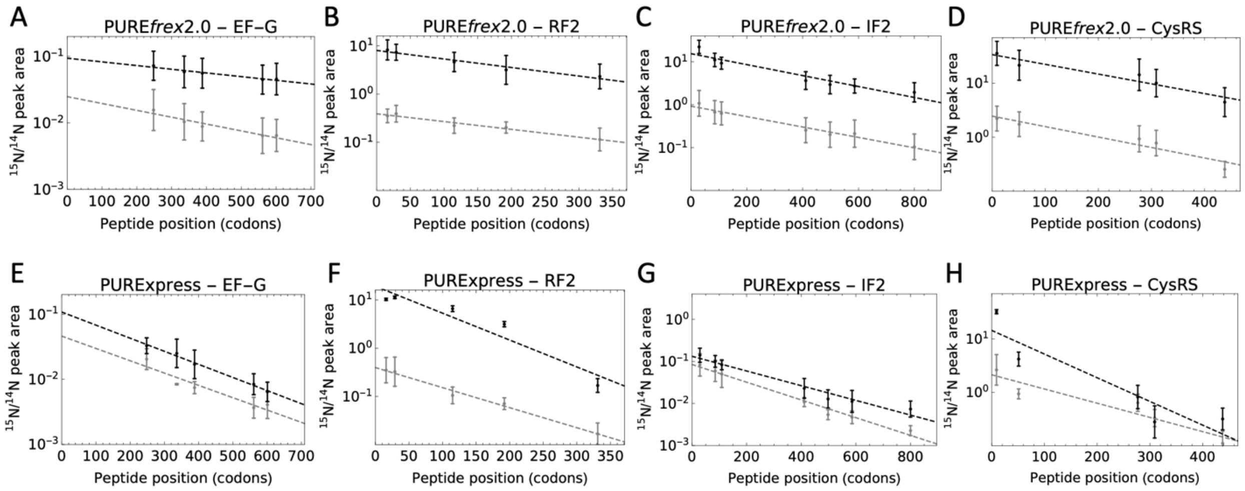

Fig. S4: ^{15}N-to-^{14}N peak area ratio as a function of the peptide position for the 20 min (gray) and 300 min (black) time points in PURE*frex*2.0 (A-D) and PURExpress (E-H) expression reactions at 37 °C. pTFM1 was used as DNA template and peptides from the annotated protein in each panel were detected. Dashed lines represent exponential fits, from which the average per-codon loss can be extracted. Values are mean ± 1 standard deviation over three experiments.

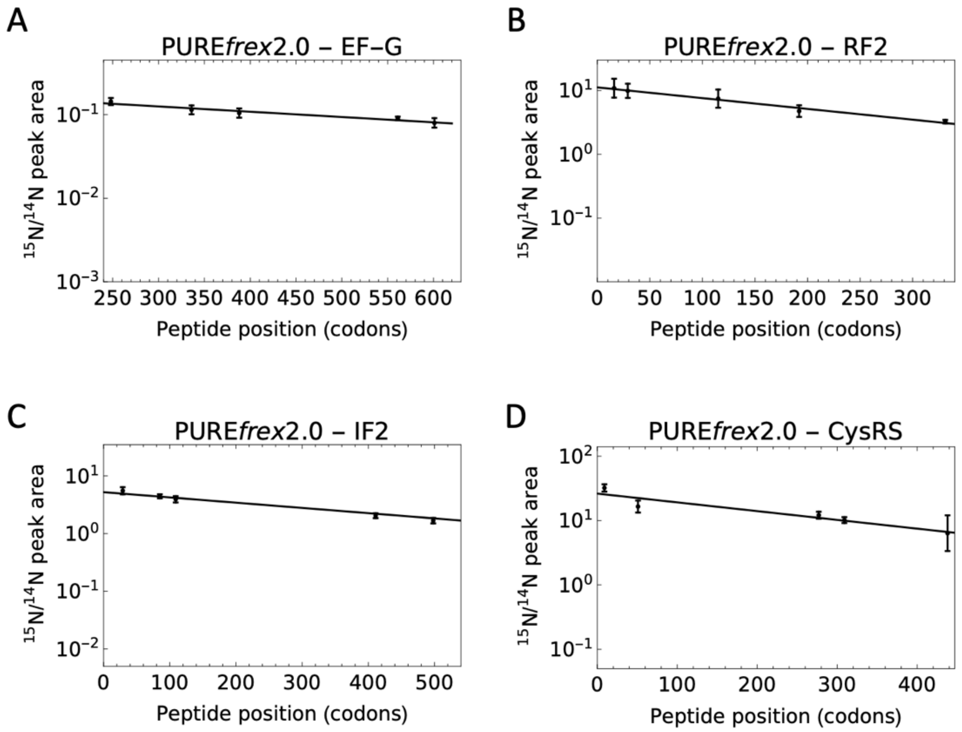

Fig. S5: Biological repeat of the experiments shown in Fig. S4 with a different batch of PURE*frex*2.0. ^{15}N-to-^{14}N peak area ratio as a function of the peptide position for 300 min time points in PURE*frex*2.0 expression reactions at 37 °C. pTFM1 was used as DNA template and peptides from the annotated protein in each panel were detected. Dashed lines represent exponential fits, from which the average per-codon loss can be extracted. Values are mean ± 1 standard deviation over three experiments.

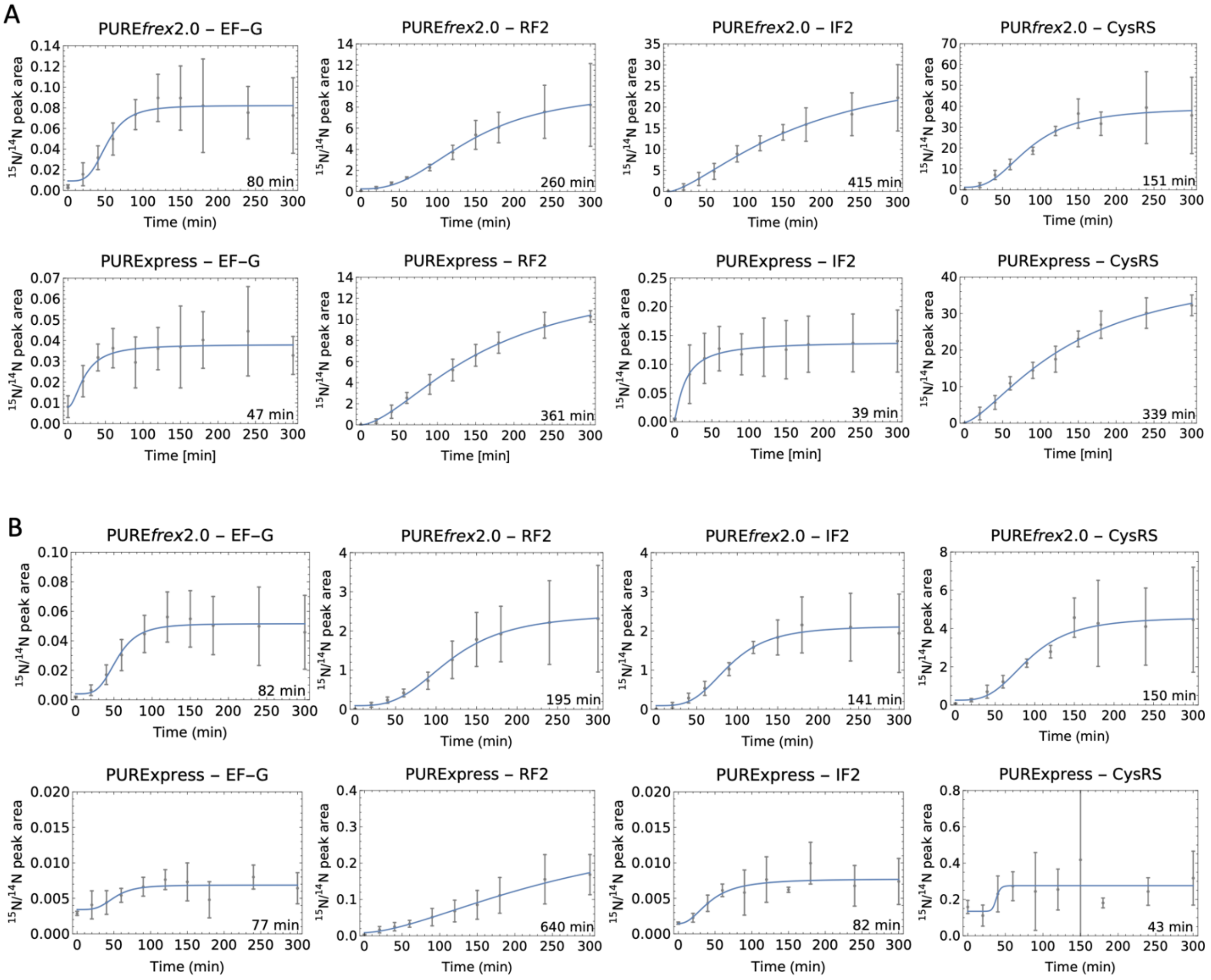

Fig. S6: Time-courses of ^{15}N-to-^{14}N peak area ratios of selected peptides for four proteins expressed from plasmid pTFM1. For each protein, the most N-terminal peptide (A), and most C-terminal peptide (B) were selected. Data were extracted from main text Fig. 3. Solid lines are fits computed from the equation: $f(t) = a + b \times t^c/(t^c + d^c)$, where t denotes time and $f(t)$ describes the peptide concentration at time t. The expression timespan (values appended in the graphs) is calculated from the fitted parameters as $2d/c + d$.

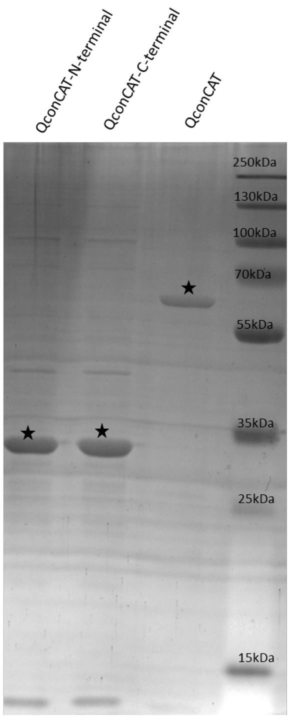

Fig. S7: Analysis of purified QconCAT proteins by gel electrophoresis. Stars indicate the protein of interest.

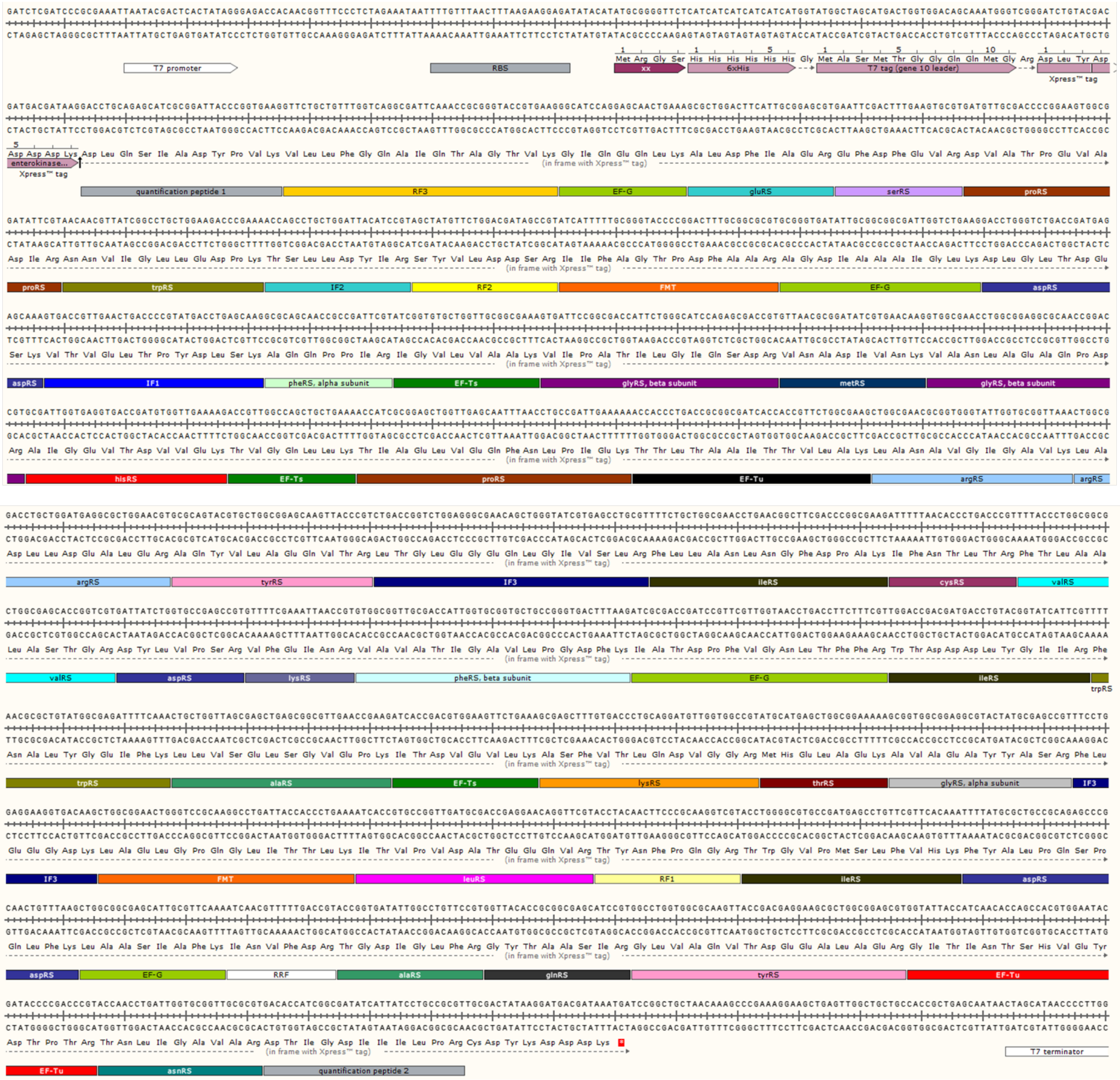

Fig. S8: Design of the QconCAT gene.

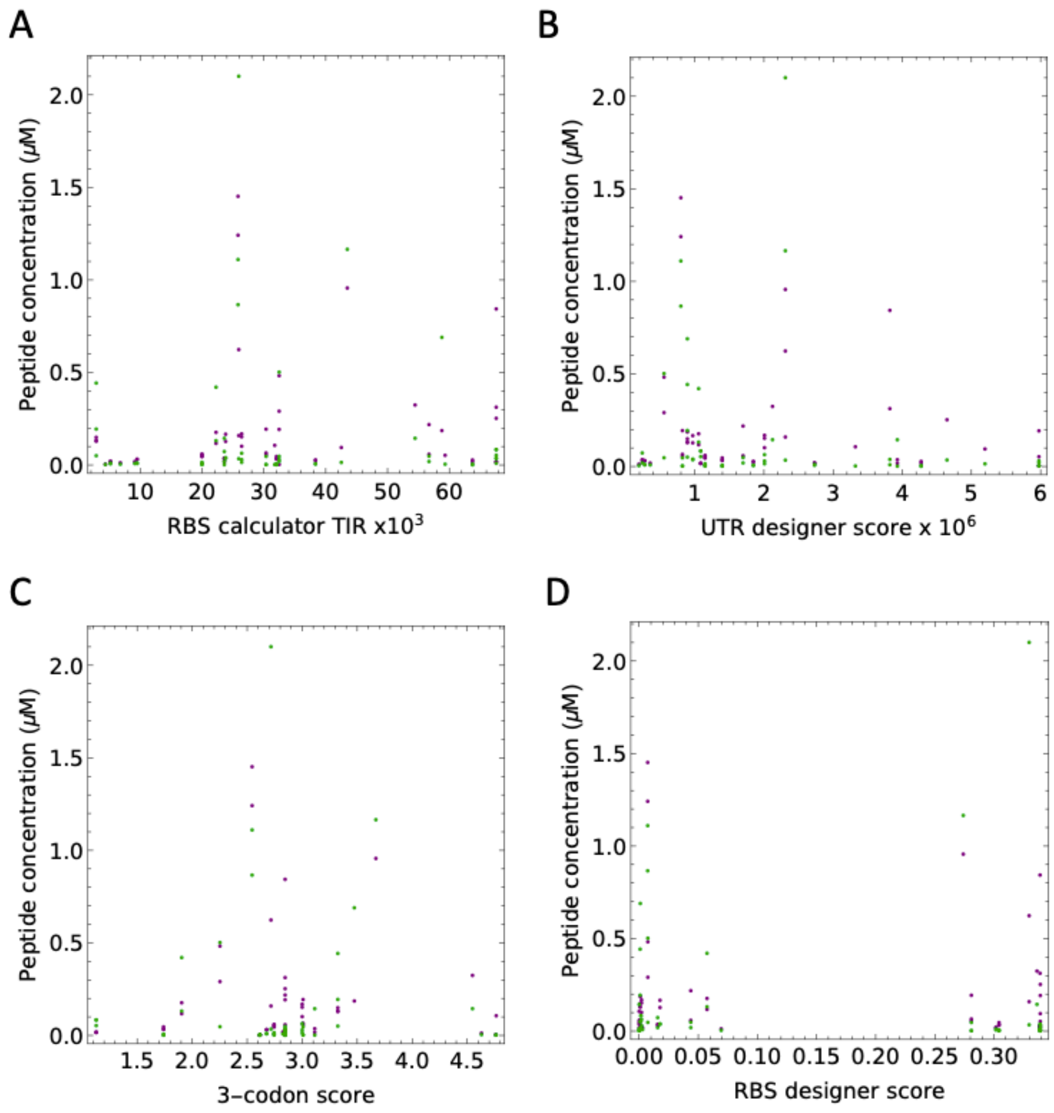

Fig. S9: Correlations of absolute peptide expression levels with the RBS calculator TIR (translation initiation rate) score (1) (A), with the UTR (untranslated region) designer score (2) (B), with a 3-codon score from (3) (C), and with the RBS designer score (4) (D). Data points for PURE*frex*2.0 and PURExpress are colored in magenta and in green, respectively. Values of Pearson correlation coefficients do not show significant correlation at the 10% level.

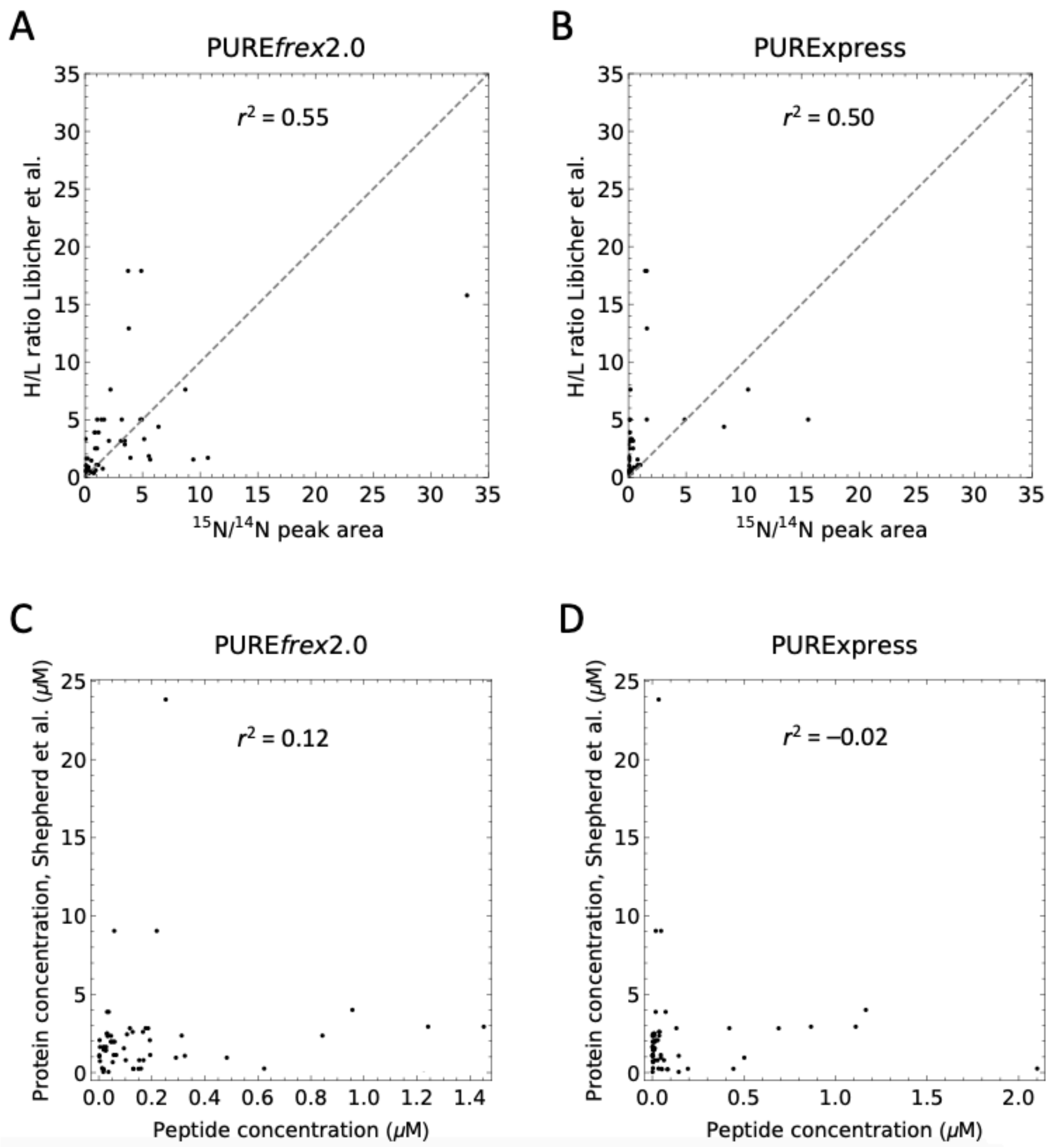

Fig. S10: Correlation analyses between our data and those published by Libicher et al. (5) (A,B) or by Shepherd et al. (6) (C,D). Values of r^2 appended to the graphs denote Pearson correlation coefficients. In refs. (5, 6), translation factors were expressed from a mix of three plasmids, either in a cell-free system (5) or in vivo (6) which could explain the rather low correlation with the expression levels observed here with cell-free expression from a single plasmid.

SUPPLEMENTARY REFERENCES

1. Salis HM, Mirsky EA, Voigt CA. Automated design of synthetic ribosome binding sites to control protein expression. *Nat Biotechnol.* 2009;27(10):946-50.
2. Seo SW, Yang J-S, Kim I, Yang J, Min BE, Kim S, et al. Predictive design of mRNA translation initiation region to control prokaryotic translation efficiency. *Metabolic Engineering*. 2013;15:67-74.
3. Verma M, Choi J, Cottrell KA, Lavagnino Z, Thomas EN, Pavlovic-Djuranovic S, et al. Short translational ramp determines efficiency of protein synthesis. bioRxiv. 2019:571059.
4. Na D, Lee D. RBSDesigner: software for designing synthetic ribosome binding sites that yields a desired level of protein expression. *Bioinformatics*. 2010;26(20):2633-4.
5. Libicher K, Hornberger R, Heymann M, Mutschler H. In vitro self-replication and multicistronic expression of large synthetic genomes. *Nature Communications*. 2020;11(1):904.
6. Shepherd TR, Du L, Liljeruhm J, Samudyata, Wang J, Sjödin MOD, et al. De novo design and synthesis of a 30-cistron translation-factor module. *Nucleic acids research*. 2017;45(18):10895-905.
